# Gelsolin's Protective Role in MASH through F‐Actin Regulation and P53 Degradation

**DOI:** 10.1002/advs.202416489

**Published:** 2025-05-20

**Authors:** Yiwei Lu, Tong Ji, Zhichao Ye, Jianing Yan, Chao Wang, Jiachen Chen, Ziyang Jin, Yongji Zhu, Xiujun Cai, Yifan Wang

**Affiliations:** ^1^ Department of General Surgery Sir Run Run Shaw Hospital Affiliated to School of Medicine Zhejiang University Hangzhou 310016 China; ^2^ National Engineering Research Center of Innovation and Application of Minimally Invasive Instruments Hangzhou 310016 China; ^3^ Zhejiang Provincial Key Laboratory of Laparoscopic Technology Sir Run Run Shaw Hospital Affiliated to School of Medicine Zhejiang University Hangzhou 310016 China

**Keywords:** F‐actin, gelsolin (GSN), metabolic‐associated steatohepatitis (MASH), P53, YAP

## Abstract

Hepatic steatosis, inflammation, and fibrosis are the hallmarks of metabolic‐associated steatohepatitis (MASH), a serious health risk. This study emphasizes how important gelsolin (GSN) is to the pathophysiology of MASH. The results show that GSN is significantly overexpressed in both MASH patients and animal models. Under MASH models, *Gsn* knockout (KO) (*Gsn* ^−/−^) mice demonstrate exacerbated hepatic steatosis, inflammation, and fibrosis, underscoring GSN's protective function. Remarkably, adeno‐associated virus (AAV)‐mediated restoration of *Gsn* substantially alleviates these pathological features, indicating its therapeutic potential. Mechanistically, the absence of GSN leads to increased F‐actin polymerization and heightened activation of Yes‐associated protein (YAP), thereby intensifying the inflammatory response. Subsequently, the experimental data identify a co‐expression relationship between GSN and MDM2, and GSN is found to facilitate the ubiquitination and subsequent degradation of P53 via MDM2, a crucial process for liver protection. These findings imply that GSN is essential for controlling important molecular pathways in MASH by encouraging P53's MDM2‐mediated degradation, which lessens the severity of hepatic steatosis. The research offers important new understandings of the molecular mechanisms of MASH and suggests GSN as a viable therapeutic target to reduce liver damage and preserve hepatic homeostasis.

## Graphical abstract

Gelsolin (GSN) maintains liver metabolic homeostasis by promoting MDM2‐mediated P53 degradation and inhibiting F‐actin/Yes‐associated protein (YAP) overactivation. Loss of GSN impairs P53 degradation, increases lipid deposition, and enhances YAP‐driven fibrosis and inflammation in metabolic‐associated steatohepatitis. These findings highlight GSN as a key regulator of lipid metabolism, cytoskeletal balance, and inflammatory signaling in liver disease.

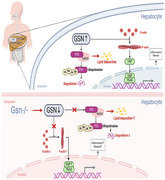

## 1 Introduction

Metabolic dysfunction‐associated fatty liver disease (MAFLD), previously known as non‐alcoholic fatty liver disease (NAFLD), is the most common chronic liver condition in Western populations.^[^ ^1^, ^2^ ^]^ It is defined as hepatic steatosis (≥5% hepatocytes with fat accumulation) in individuals with overweight/obesity, type 2 diabetes, or metabolic dysregulation, regardless of alcohol consumption or other liver diseases. It replaces the term NAFLD to emphasize the central role of metabolic dysfunction in pathogenesis. A significant portion of patients with MAFLD, ≈25%, will progress to metabolic‐dysfunction‐associated steatohepatitis, formerly termed non‐alcoholic steatohepatitis (NASH).^[^ ^3^ ^]^ Metabolic‐associated steatohepatitis (MASH) is marked by steatosis, liver inflammation, hepatocyte injury (ballooning degeneration), and fibrosis.^[^ ^4^ ^]^ This progressive stage poses a heightened risk of developing severe liver complications, including cirrhosis, hepatocellular carcinoma, and ultimately increased liver‐related mortality.^[^ ^5^ ^]^

Despite the growing prevalence of MASH worldwide, driven by rising rates of obesity and metabolic disorders, effective therapeutic options remain limited. Diagnosis typically involves histological examination through liver biopsy, though non‐invasive methods like magnetic resonance elastography and serum biomarkers are gaining use.^[^ ^6^ ^]^ Current management focuses on lifestyle interventions and controlling metabolic risk factors.^[^ ^7^ ^]^ Ongoing researches aim to elucidate the molecular mechanisms driving MASH, seeking to identify novel targets for pharmacological treatment to better address this increasingly significant health concern.

Gelsolin (GSN) is a critical actin‐regulating protein involved in cellular dynamics and pathophysiology. As a major actin‐binding protein, GSN regulates actin filament turnover and cytoskeletal rearrangements, affecting cellular motility, wound healing, and apoptosis.^[^ ^8^ ^]^ Aberrant GSN functions are correlated with tumor progression and metastasis in cancer. Prostate and breast cancers have been shown to have elevated GSN levels, which may act as biomarkers for the prognosis of the disease.^[^ ^9^, ^10^ ^]^ As for the role of GSN in the liver, a proteomics study on hepatocellular carcinoma (HCC) found that GSN expression was elevated in HCC tissues.^[^ ^11^ ^]^ Another study demonstrated that, as an important actin‐binding protein, GSN promotes HCC invasion and metastasis through its synergistic interaction with the actin‐associated molecular transfer chain(actin‐CD44‐MMPs).^[^ ^12^ ^]^ In addition, GSN dysregulation is associated with autoimmune disorders, like systemic lupus erythematosus (SLE), where it contributes to immune response abnormalities and tissue damage.^[^ ^13^ ^]^

Yes‐associated protein (YAP) plays a crucial role in cellular growth and tissue homeostasis as a transcriptional co‐activator in the Hippo signaling pathway.^[^ ^13^ ^]^ YAP interacts with cytoskeletal proteins such as F‐actin, which is essential for maintaining cell shape, motility, and mechanical stability. The interaction between YAP and F‐actin influences YAP's subcellular localization and transcriptional activity.^[^ ^13^ ^]^ Increased F‐actin polymerization enhances YAP's nuclear localization and activity, promoting cell proliferation and survival. Disruption of F‐actin dynamics affects YAP function, with implications for cancer and fibrotic diseases.^[^ ^13^ ^]^ Research has shown that altered YAP activity, due to changes in F‐actin dynamics, contributes to tumorigenesis and metastasis. Elevated YAP levels and disrupted actin cytoskeleton are common in various cancers, indicating their role in tumor progression.^[^ ^14^, ^15^ ^]^ Furthermore, YAP‐F‐actin interactions are implicated in fibrotic diseases, where abnormal YAP activation and actin remodeling contribute to excessive tissue scarring.^[^ ^16^ ^]^

As the “guardian of the genome,” the P53 tumor suppressor protein controls DNA repair, apoptosis, and the advancement of the cell cycle. MDM2, an E3 ubiquitin ligase that targets P53 for destruction, modulates its activity. The P53‐MDM2 axis is crucial for balancing cellular responses to stress.^[^ ^17^ ^]^ In MASH, P53 is activated in response to oxidative stress and lipid accumulation, regulating hepatic lipid metabolism, apoptosis, and fibrosis.^[^ ^18^, ^19^ ^]^ Dysregulation of the P53‐MDM2 interaction in MASH can exacerbate disease progression, with MDM2 overexpression linked to increased liver fibrosis.^[^ ^20^ ^]^ Studies support that P53 activation in MASH correlates with liver apoptosis and inflammation. Interventions targeting the P53‐MDM2 interaction, such as MDM2 inhibitors, show potential in mitigating liver damage in preclinical models.^[^ ^17^ ^]^ Understanding the P53‐MDM2 relationship provides insights into therapeutic strategies for MASH and highlights its broader implications in metabolic diseases.

This study sought to clarify the possible protective role of GSN in the pathophysiology of MASH. The results demonstrated that Hepatocytes exhibit an upregulation of GSN as MASH progresses. Comprehensive RNA sequencing and functional analysis revealed that *Gsn* deficiency in MASH exacerbates inflammation, fibrosis, and lipid buildup in liver tissue. Mechanistically, GSN plays a role in cytoskeletal dynamics; its absence leads to impaired F‐actin depolymerization and subsequent overactivation of F‐actin, which in turn results in excessive activation of YAP, thereby aggravating hepatic fibrosis and inflammatory responses. In addition, GSN promotes the binding of MDM2 to P53, facilitating the proper ubiquitination and proteasomal degradation of P53. The loss of GSN results in elevated P53 protein levels, contributing to severe hepatic steatosis. These findings underline GSN's potential as a therapeutic target in human MASH and provide strong support for GSN as a functional candidate gene in the control of hepatic steatosis and inflammation‐fibrosis responses.

## 2 Results

### 2.1 GSN Expression Correlates with MASH Severity in Human and Mouse Models

**Figure 1:**
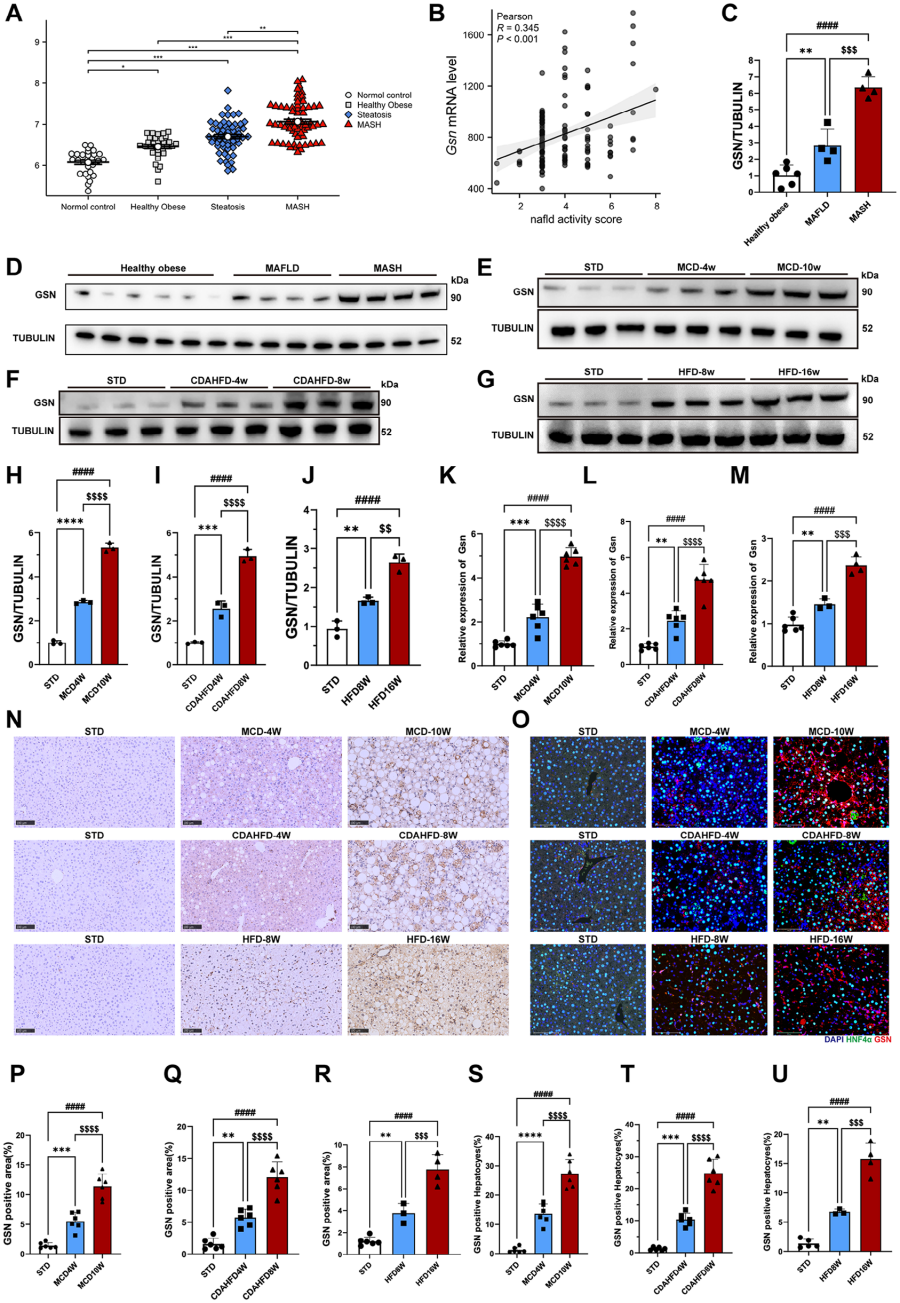
GSN expression correlates with MASH severity in MASH samples. The information of 183 patients was merged from the GEO database (GSE48452, GSE61620, GSE33814, and GSE63067), and the expression levels of *Gsn* in patients under different conditions were calculated; liver samples of 106 patients of different NAFLD activity scores were harvested and conducted transcriptome profiling. *Gsn* mRNA levels are presented as mean ± standard deviation (SD). A,B) The relationship between *Gsn* mRNA levels and NAFLD activity scores was evaluated using Pearson correlation analysis. C) Quantitative data of Western blot results from panel (D); D) Western blot analysis was performed to assess GSN protein expression in human liver tissue samples (*n* = 4–6/group); E–G) the expression levels of GSN in liver tissues were evaluated at different time points in mice fed with the methionine and choline‐deficient diet (MCD, E), the choline‐deficient, l‐amino acid‐defined, high‐fat diet (CDAHFD, F), and the high‐fat diet (HFD, G) using Western blot analysis, with mice on a standard diet (STD) serving as the control group for each dietary intervention (*n* = 3–6/group); H–J) quantitative data of Western blot results from panel (E)–(G); the expression levels of *Gsn* in liver tissues were evaluated at different time points in mice fed with K) MCD, L) CDAHFD, and M) HFD using quantitative PCR analysis, with mice on a standard diet (STD) serving as the control group for each dietary intervention (*n* = 3–6/group); N) representative immunohistochemical staining was used to evaluate the expression of GSN in liver tissue sections from mice fed with MCD, CDAHFD, and HFD diets, with mice on a STD serving as the control group for each dietary intervention (*n* = 3–6/group), Scale bars indicate 100 µm; O) representative immunofluorescence staining was performed to evaluate GSN expression in liver sections from mice fed with MCD diet, CDAHFD, and HFD, with mice on a STD serving as the control group for each dietary intervention (*n* = 3–6/group), Scale bars indicate 100 µm; P–R) quantitative analysis of GSN‐positive areas in immunohistochemistry from panel (N). S–U) Quantitative analysis of GSN‐positive hepatocytes in immunofluorescence staining from panel (O); data were expressed as the means ± standard deviation (SD). Significant differences were determined using one‐way ANOVA as appropriate. A two‐tailed *p* < 0.05 was considered statistically significant (*p < 0.05, **p < 0.01, ***p < 0.005, ****p < 0.001, n.s., no significance).

To investigate the potential role of GSN in MASH, we initially examined the transcription levels of GSN in human liver samples categorized as normal control, healthy obese, steatosis, and MASH. We utilized data from four separate microarray datasets accessible through the Gene Expression Omnibus (GEO) repository (GSE48452, GSE61620, GSE33814 and GSE63067) comprising 183 samples. The analysis demonstrated a significant elevation in *Gsn* mRNA expression in liver tissues affected by MASH relative to normal tissues (**Figure** **1**A). In addition, *Gsn* mRNA levels exhibited a positive correlation with the NAFLD activity score in the GSE193006 dataset (Figure 1B). Subsequently, in our patient tissue samples, we observed that the protein expression levels of GSN were consistent with the findings from the public database. Specifically, the hepatic GSN protein levels in patients diagnosed with MASH were more than four times higher than those in livers from healthy obese individuals. In contrast, the GSN expression levels in the livers of patients with MAFLD showed a less pronounced increase compared to normal controls but remained statistically significant (Figure 1C,D). These findings further support the differential regulation of GSN in metabolic dysfunction‐associated liver diseases and highlight its potential role in the pathophysiological progression of MASH.

To validate this phenomenon, we developed three diet‐induced MASH mouse models: methionine‐ and choline‐deficient diet (MCD), choline‐deficient l‐amino acid‐defined high‐fat diet (CDAHFD) and high‐fat diet (HFD) to thoroughly examine the role of GSN in MASH. To investigate the progression of liver pathology in different dietary models, we collected liver tissues and serum from mice fed the MCD diet at weeks 4 and 10 (Figure S1A, Supporting Information), the CDAHFD diet at weeks 4 and 8 (Figure S1D, Supporting Information), and the HFD diet at weeks 8 and 16 (Figure S1G, Supporting Information) for subsequent analyses. Hematoxylin and eosin (HE) staining and Sirius Red staining demonstrated that mice in the MCD and CDAHFD groups exhibited significant inflammation and fibrosis as early as week 4 (Figure S1A,D, Supporting Information). This severity further escalated with prolonged feeding, as evidenced by markedly elevated inflammation and fibrosis levels at week 10 in the MCD group (Figure S1A, Supporting Information) and week 8 in the CDAHFD group (Figure S1D, Supporting Information) compared to the normal diet controls. Consistently, serum levels of ALT, AST (Figure S1B,E, Supporting Information), and inflammatory cytokines, as assessed by ELISA (Figure S1J,K, Supporting Information), confirmed the severe inflammatory response in these groups. Hydroxyproline assays aligned with the Sirius Red staining results, indicating significantly increased fibrosis in the liver tissues of the MCD and CDAHFD mice (Figure S1B,E, Supporting Information).

In contrast, mice in the HFD group exhibited only mild inflammation and fibrosis after 8 weeks of feeding, in stark contrast to the MCD and CDAHFD groups at similar early time points (Figure S1G, Supporting Information). By week 16, HFD‐fed mice showed a notable increase in inflammation and fibrosis compared to normal diet controls, but these changes were still less pronounced than those observed in the MCD (week 10) and CDAHFD (week 8) groups (Figure S1G, Supporting Information). These observations were corroborated by HE staining, Sirius Red staining, and serum assays, further highlighting the distinct progression patterns of liver pathology in the HFD model (Figure S1H,L, Supporting Information).

Regarding another hallmark of MASH, hepatic steatosis, Oil Red O staining (Figure S1A,D,G, Supporting Information), liver triglyceride (TG) (Figure S1B,E,H, Supporting Information) measurements, and quantitative PCR (q‐PCR) analysis of lipid metabolism‐related genes (Figure S1C,F,I, Supporting Information) revealed significant differences among the groups. HFD‐fed mice demonstrated substantial lipid droplet accumulation as early as week 8, whereas the MCD and CDAHFD groups showed minimal changes at week 4. By week 16, HFD‐fed mice exhibited severe hepatic steatosis, with TG levels in liver tissues nearly double those observed in the MCD (week 10) and CDAHFD (week 8) groups. Furthermore, q‐PCR analysis revealed a more pronounced upregulation of lipid metabolism‐related genes in the HFD group at week 16 compared to the other models.

In conclusion, mice subjected to the three dietary regimens demonstrated clear pathological features of MAFLD (MCD at week 4, CDAHFD at week 4, and HFD at week 8) and MASH (MCD at week 10, CDAHFD at week 8, and HFD at week 16) at their respective time points, as validated through a series of comprehensive experimental analyses. These findings confirm the successful establishment of dietary‐induced mouse models representing progressive stages of metabolic dysfunction‐associated liver disease. The reproducibility and distinct pathological characteristics of these models provide a robust foundation for investigating the mechanisms underlying MAFLD and MASH and for evaluating potential therapeutic strategies.

Subsequently, we examined the expression of GSN in the three dietary models at various time points. Western blot (WB) (Figure 1E–J), q‐PCR (Figure 1K–M), immunohistochemistry (Figure 1N,P–R), and immunofluorescence analyses (Figure 1O,S–U) revealed a significant upregulation of GSN at both the protein and transcriptional levels in mice fed the MCD diet for 4 weeks and the CDAHFD diet for 4 weeks, with an approximately twofold increase compared to normal diet controls. Notably, in the livers of MCD‐fed mice at week 10 and CDAHFD‐fed mice at week 8, GSN expression was even more markedly elevated, reaching a four‐ to sixfold increase relative to controls. In contrast, the upregulation of GSN in HFD‐fed mice was less pronounced. By week 16, GSN expression levels in the livers of HFD‐fed mice increased by nearly threefold, a finding corroborated by IHC and immunofluorescence analyses.

These results collectively validate our observations from public databases and patient samples, demonstrating that GSN expression progressively increases with the severity of MASH pathology. This trend was consistent in both murine and human liver tissues, where GSN expression showed a gradual rise from MAFLD to MASH stages.

### 2.2 GSN Attenuates Lipid Accumulation and Inflammation In Vivo

**Figure 2:**
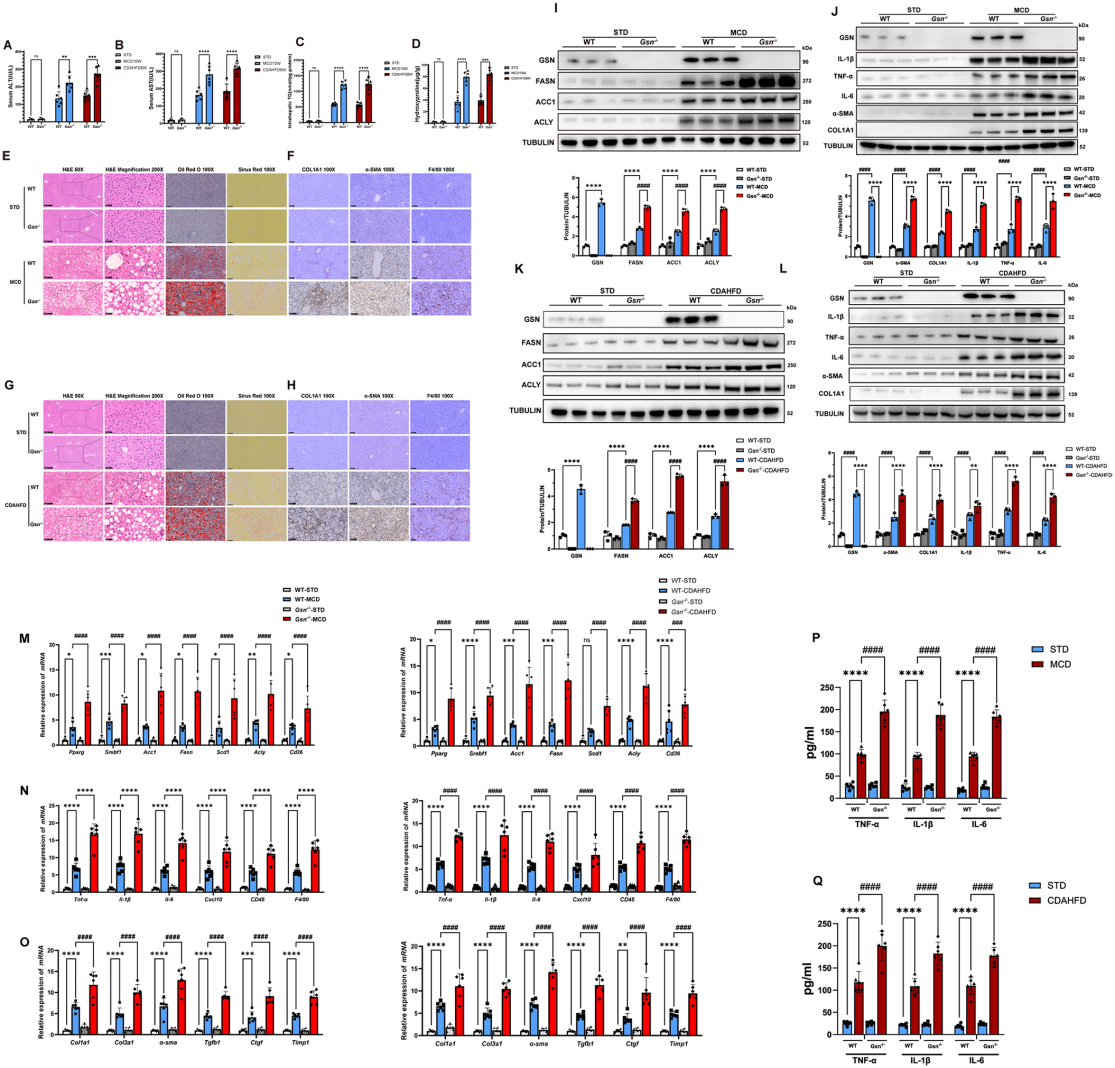
GSN alleviates the progression of MASH in in vivo mouse models. A,B) Serum levels of ALT and AST in the specified groups of mice (*n* = 6/group). C) Hepatic triglyceride levels in the liver tissues of the specified groups of mice (*n* = 6/group); D) hepatic hydroxyproline levels in the liver tissues of the specified groups of mice (*n* = 6/group); E) representative images from hematoxylin and eosin (H&E) staining (50×, Scale bars: 250 µm), H&E magnification (200×, Scale bars: 50 µm), Oil Red O staining (100×, Scale bars: 250 µm), sirius red staining (100×, Scale bars: 250 µm) were obtained for each MCD‐fed group (*n* = 6/group); F) representative immunohistochemical (IHC) staining images of α‐SMA (100×, Scale bars: 100 µm), COL1A1 (100×, Scale bars: 100 µm), F4/80 (100×, Scale bars: 100 µm) were obtained for each MCD‐fed group (*n* = 6/group); G) representative images from hematoxylin and eosin (H&E) staining (50×, Scale bars: 250 µm), H&E magnification (200×, Scale bars: 50 µm), Oil Red O staining (100×, Scale bars: 250 µm), sirius red staining (100×, Scale bars: 250 µm) were obtained for each CDAHFD‐fed group (*n* = 6/group); H) representative IHC staining images of α‐SMA (100×, Scale bars: 100 µm), COL1A1 (100×, Scale bars: 100 µm), F4/80 (100×, Scale bars: 100 µm) were obtained for each CDAHFD‐fed group (*n* = 6/group); I,J) the expression levels of GSN, FASN, ACC1, ACLY, IL‐1β, TNF‐α, IL‐6, α‐SMA, and COL1A1 in liver tissues were evaluated at different time points in mice fed with MCD using Western blot analysis for each experimental group (*n* = 3/group); K,L) the expression levels of GSN, FASN, ACC1, ACLY, IL‐1β, TNF‐α, IL‐6, α‐SMA, and COL1A1 in liver tissues were evaluated at different time points in mice fed with CDAHFD using Western blot analysis for each experimental group (*n* = 3/group); quantitative PCR analyses were performed to assess the expression of M) lipid metabolism molecules, N) inflammatory response markers, and O) fibrosis‐related markers in liver tissues for each experimental group (*n* = 6/group); P) ELISA results for inflammatory cytokines (TNF‐α, IL‐1β, IL‐6) in serum of the MCD‐fed groups of mice (*n* = 6/group); Q) ELISA results for inflammatory cytokines (TNF‐α, IL‐1β, IL‐6) in serum of the CDAHFD‐fed groups of mice (*n* = 6/group); data were expressed as the means ± standard deviation (SD). Significant differences were determined using Student's *t*‐test or one‐way ANOVA as appropriate. A two‐tailed *p* < 0.05 was considered statistically significant (*p < 0.05, **p < 0.01, ***p < 0.005, ****p < 0.001, n.s., no significance).

By utilizing three distinct dietary regimens to model liver disease, we observed that GSN expression increased only modestly in mice on a HFD compared to the other dietary models, both at the transcriptional and protein levels. To further investigate this, we generated a global *Gsn* knockout (*Gsn^−/−^*) mouse model. *Gsn^−/−^* mice, along with wild‐type (WT) controls, were subjected to MCD and CDAHFD regimens to establish MASH models. Our studies revealed that global *Gsn* deletion did not affect reproductive function, nor did it induce liver damage under standard conditions (**Figure** **2**E,G). We found that *Gsn* knockout mice exhibited higher pathology score (NAS) in both the MCD and CDAHFD groups, indicating that GSN deficiency has a significant impact on the liver during the progression of MASH (Figure S3B,H, Supporting Information).

Serum analysis revealed significantly elevated levels of alanine aminotransferase (ALT) and aspartate aminotransferase (AST) in *Gsn* knockout (*Gsn* ^−/−^) mice following MCD and CDAHFD dietary treatments, compared to wild‐type controls (Figure 2A,B). In addition, triglyceride assays of liver tissues indicated a notable increase in triglyceride accumulation in *Gsn* ^−/−^ mice, reflecting more severe hepatic lipid accumulation and steatosis (Figure 2C). Hydroxyproline assays further confirmed that *Gsn* ^−/−^ mice exhibited significantly higher levels of hepatic fibrosis compared to wild‐type MASH mice (Figure 2D). Histopathological examinations provided more direct evidence of aggravated liver pathology in *Gsn* ^−/−^ mice. H&E staining, Oil Red O staining, and Sirius Red staining (Figure 2E,G and Figure S3C–G,I–M, Supporting Information) revealed more pronounced hepatic steatosis, inflammation, and fibrosis in the liver tissues of *Gsn* ^−/−^ mice compared to wild‐type controls. Immunohistochemical (IHC) staining further corroborated these findings, showing significantly elevated levels of fibrosis markers (α‐SMA, COL1A1) and macrophage infiltration markers (F4/80) in the liver tissues of *Gsn* ^−/−^ mice (Figure 2F,H).

Collectively, these results indicate that the loss of GSN exacerbates liver injury, inflammation, and fibrosis in mice fed MCD and CDAHFD diets. The findings underscore the critical role of GSN in regulating hepatic metabolism, inflammation, and fibrogenesis, highlighting its potential as a therapeutic target for metabolic liver diseases, especially MASH.

To identify the molecular pathways regulated by GSN in the liver under metabolic stress, we conducted RNA sequencing on liver samples from WT and *Gsn* ^−/−^ mice fed an MCD diet for 10 weeks (*n* = 3 per group) (Figure S2A, Supporting Information). Unsupervised hierarchical clustering segregated the samples into two distinct clusters. Subsequent gene set enrichment, variation analysis, and heatmap visualization revealed significant alterations in pathways related to inflammation, lipid metabolism, and oxidative phosphorylation due to *Gsn* knockout (Figure S2B, Supporting Information). In addition, as an important cytoskeletal protein, actin‐related pathways were also notably impacted (Figure S2B, Supporting Information), This finding is consistent with the physiological role of GSN as a crucial cytoskeletal protein. GSN is known to regulate actin filament dynamics, which plays a pivotal role in maintaining cellular structure and function.^[^ ^21^ ^]^ We further validated the gene expression profiles in the liver tissues of both groups, confirming that GSN significantly affects genes associated with lipid metabolism, inflammation and oxidative stress (Figure S2C,D, Supporting Information). Subsequent WB and qPCR results further validated these findings. In liver tissues of *Gsn* ^−/−^ mice fed a MASH diet, we observed a significant upregulation of genes related to lipid metabolism (Figure 2I,K,M), as well as those associated with inflammation and fibrosis (Figure 2J,L,N,O), compared to wild‐type MASH mice. These changes were evident at both the transcriptional and protein expression levels, highlighting the exacerbated pathological processes in the absence of GSN. We also observed that GSN has little effect on the fatty acid β‐oxidation‐related pathways, suggesting that GSN may have a greater impact on lipid synthesis and transport processes in the liver under metabolic stress in the MASH environment (Figure S3N,O, Supporting Information).

Furthermore, ELISA results demonstrated increased serum levels of inflammatory cytokines in *Gsn* ^−/−^ mice, suggesting an intensified inflammatory response associated with GSN deficiency (Figure 2P,Q).

Collectively, these findings indicate that the absence of GSN results in a heightened pathological response under dietary conditions that induce MASH, highlights the essential function of GSN in mitigating liver damage and maintaining hepatic homeostasis during the progression of MASH.

### 2.3 Restoration of GSN Alleviates Hepatic Injury in MCD‐Fed *Gsn* ^−/−^ Mice

**Figure 3:**
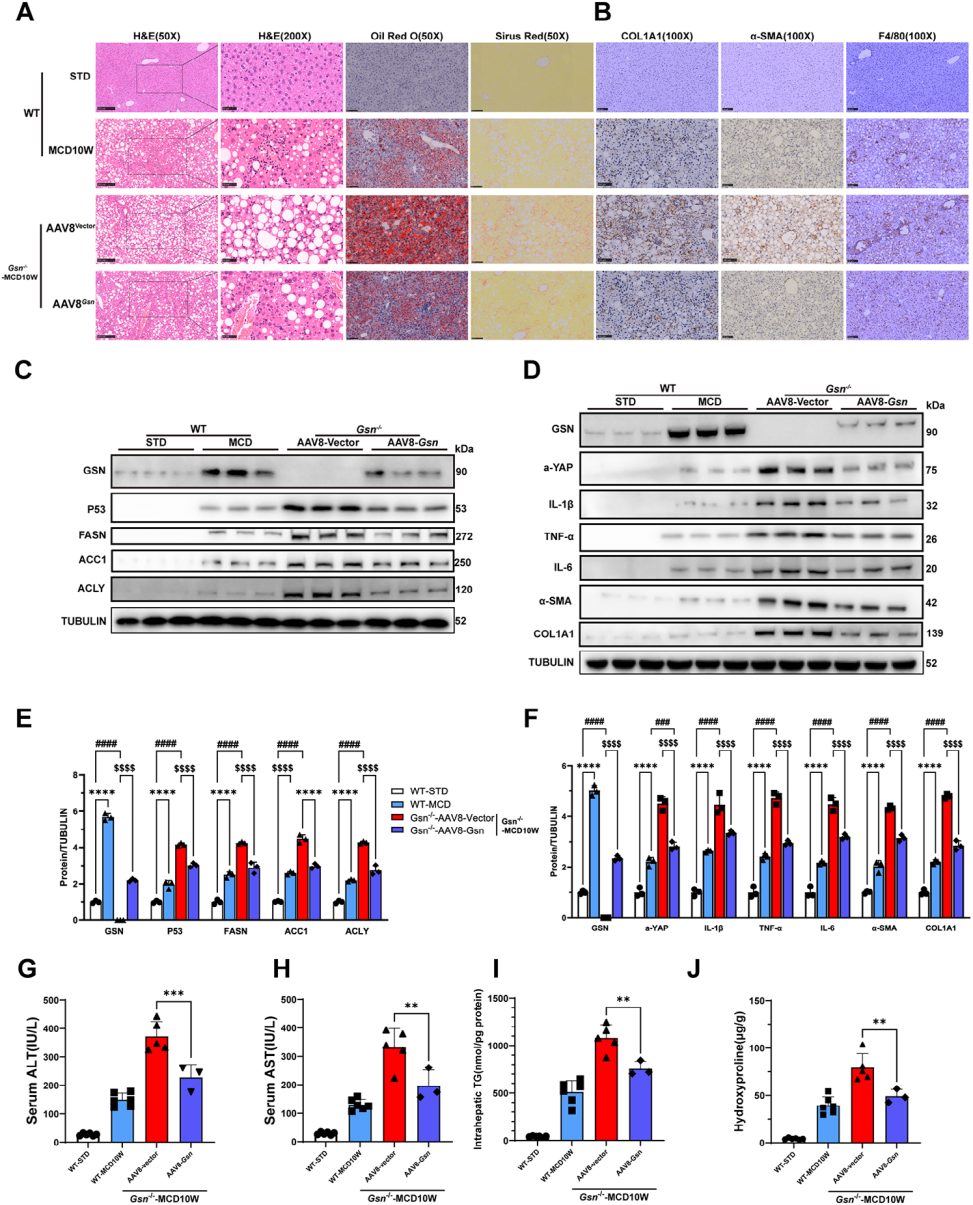
Restoration of GSN alleviates hepatic injury in MCD‐fed *Gsn* ^−/−^ mice. A) Representative images from hematoxylin and eosin (H&E) staining (50×, Scale bars: 250 µm), H&E magnification (200×, Scale bars: 50 µm), Oil Red O staining (50×, Scale bars: 250 µm), sirius red staining (50×, Scale bars: 250 µm) were obtained for each experimental group (*n* = 3–6/group); B) representative immunohistochemical (IHC) staining images of α‐SMA (100×, Scale bars: 100 µm), COL1A1 (100×, Scale bars: 100 µm), F4/80 (100×, Scale bars: 100 µm) were obtained for each experimental group (*n* = 3–6/group); C–F) the expression levels of GSN, FASN, ACC1, ACLY, IL‐1β, TNF‐α, IL‐6, α‐SMA, and COL1A1 in liver tissues were evaluated at different time points in mice fed with MCD using Western blot analysis for each experimental group (*n* = 3/group); G,H) serum levels of ALT and AST in the specified groups of mice (*n* = 3–6/group). I) Hepatic triglyceride levels in the liver tissues of the specified groups of mice (*n* = 3–6/group); J) hepatic hydroxyproline levels in the liver tissues of the specified groups of mice (*n* = 3–6/group); data were expressed as the means ± standard deviation (SD). Significant differences were determined using Student's *t*‐test or one‐way ANOVA as appropriate. A two‐tailed *p* < 0.05 was considered statistically significant (*p < 0.05, **p < 0.01, ***p < 0.005, ****p < 0.001, n.s., no significance).

To advance our understanding of GSN's role in the development of MASH, we constructed an adeno‐associated virus serotype 8 (AAV8) vector to overexpress GSN. This vector was administered via tail vein injection to *Gsn* knockout (*Gsn* ^−/−^) mice to restore GSN expression in the absence of the gene, with AAV8 empty vector‐injected mice serving as controls. Both groups of mice were then subjected to a 10‐week MCD diet (Figure S4A, Supporting Information). WB analyses confirmed successful restoration of GSN expression in the livers of *Gsn* ^−/−^ mice. Compared to the AAV8 empty vector group, the GSN expression in the liver of the *Gsn*‐supplemented mice was elevated to approximately half the level observed in wild‐type mice on the MCD diet (**Figure** **3**C–F).

**Figure 4:**
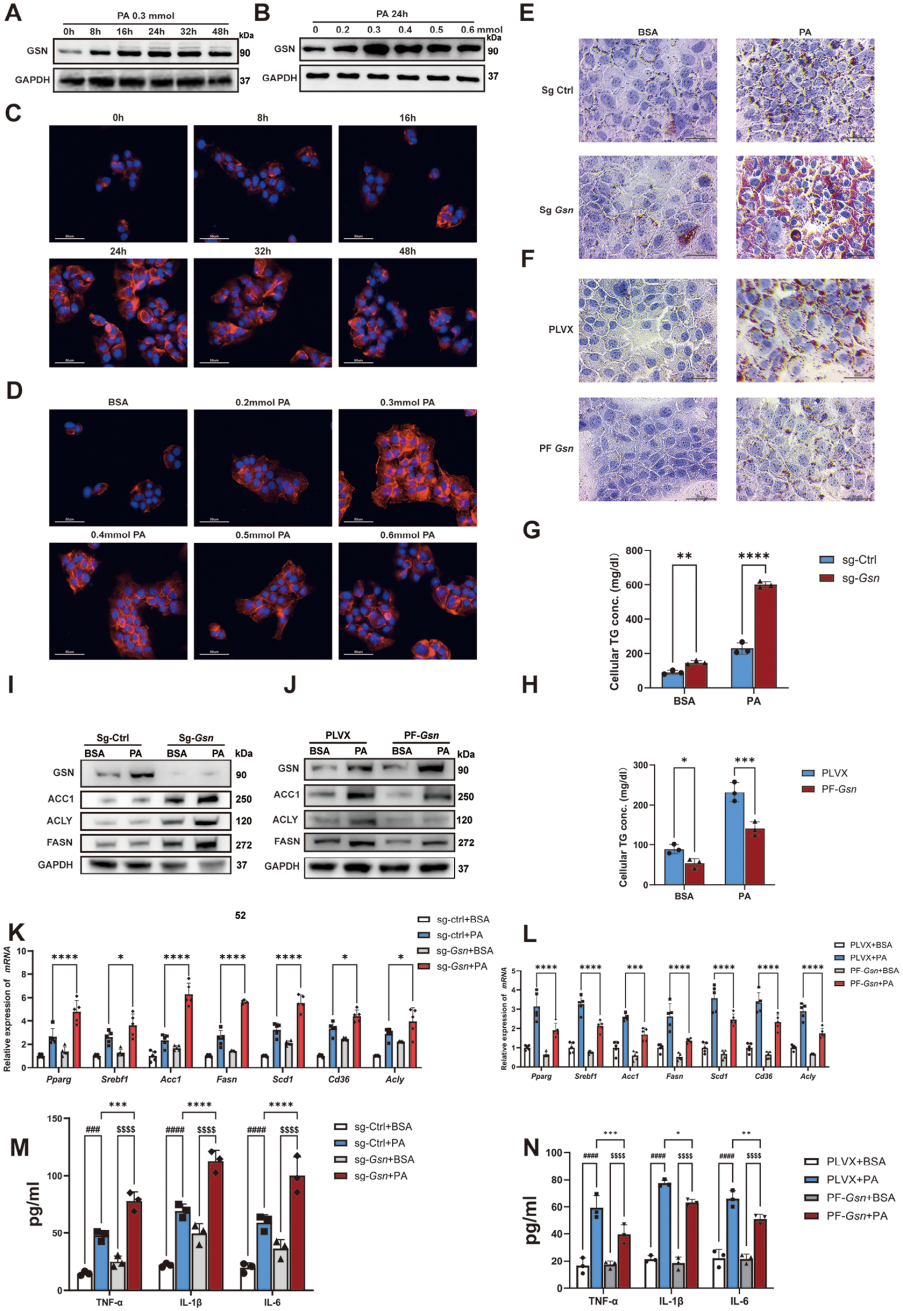
A) GSN Inhibits inflammatory responses and lipid accumulation in AML12 cells. B) Western blots analyses were performed to evaluate the expression of GSN in AML12 cells treated with 0.3 × 10^−3^ m palmitic acid (PA) at different time points, as well as in cells treated with varying concentrations of PA for 24 h; C,D) immunofluorescence analyses were performed to evaluate the expression of GSN in AML12 cells treated with 0.3 × 10^−3^ m palmitic aciPA at different time points, as well as in cells treated with varying concentrations of PA for 24 h; E,F) representative images of Oil Red O staining levels in *Gsn* knockout cells, overexpressing cells, and corresponding control cells treated with BSA or PA for 24 h; G,H) triglyceride levels in *Gsn* knockdown cells, overexpressing cells, and corresponding control cells treated with BSA or PA for 24 h. Data are from three independent experiments. Scale bars, 20 and 25 µm; I,J) protein expression levels of lipid metabolism genes in *Gsn* knockout and overexpressing cells under PA treatment. K,L) Relative mRNA levels of lipid metabolism genes in *Gsn* knockout and overexpressing cells under PA treatment (*n* = 3 independent experiments, mRNA expression levels were normalized to β‐actin). M,N) ELISA results for inflammatory cytokines (TNF‐α, IL‐1β, IL‐6) in the cell supernatants of *Gsn* knockout and overexpressing cells under BSA or PA treatment (*n* = 3 independent experiments); data were expressed as the means ± standard deviation (SD). Significant differences were determined using Student's *t*‐test or one‐way ANOVA as appropriate. A two‐tailed *p* < 0.05 was considered statistically significant (*p < 0.05, **p < 0.01, ***p < 0.005, ****p < 0.001, n.s., no significance).

Subsequent histological analyses, including H&E staining, Sirius Red staining, Oil Red O staining (Figure 3A), as well as IHC for fibrosis markers (α‐SMA, COL1A1) and infiltrating macrophage markers F4/80 (Figure 3B and Figure S4B, Supporting Information), demonstrated significant improvements in hepatic steatosis, inflammation, and fibrosis in the GSN‐supplemented group compared to the AAV8 empty vector group. Further molecular analyses by Western blot and qPCR confirmed these results, showing a reduction in the expression of genes related to lipid metabolism, inflammation, and fibrosis at both the transcriptional and protein levels in the GSN‐restored group (Figure 3C–F and Figure S4C–E, Supporting Information). These findings were also validated by serum assays, including ALT (**Figure** **4**G), AST (Figure 3H), and ELISA (Figure S4F–H, Supporting Information) to measure key inflammatory cytokines (TNF‐α, IL‐1β, IL‐6). In addition, triglyceride content (Figure 3I) and hydroxyproline levels (Figure 3J) in liver tissues provided further quantification supporting the reversal of hepatic injury and fibrosis. These results collectively indicate that GSN plays a crucial protective role in maintaining liver health, particularly in the context of MASH.

Mechanistically, overexpression of GSN facilitated the normal functioning of metabolic pathways, mitigating the adverse effects of lipid overload in the liver. These findings underscore the pivotal role of GSN in regulating hepatic metabolism and inflammation, especially in the pathogenesis of MASH. The restoration of GSN expression, therefore, suggests a potential therapeutic approach for mitigating liver injury in metabolic disorders such as MASH.

### 2.4 GSN Inhibits Inflammatory Responses and Lipid Accumulation in Hepatocytes

Furthermore, by treating the mouse hepatocyte cell line AML12 with palmitic acid (PA), we observed a proportional increase in GSN expression with higher PA concentrations and longer treatment durations (Figure 4A,B). Our study demonstrated that GSN expression peaked when treated with 0.3 mmol of PA for 24 h. Immunofluorescence analysis confirmed these findings, further supporting the notion that metabolic stress enhances GSN expression in vitro (Figure 4C,D). The quantitative results from both Western blot and immunofluorescence analyses further precisely validated this finding (Figure S5A–D, Supporting Information). Next, we infected AML12 cells with lentiviruses carrying two different sgRNA sequences and found that the first lentivirus achieved more than 90% knockdown of GSN expression in AML12 cells at both transcription and protein levels (Figure S5E–G, Supporting Information). Therefore, we selected this lentivirus for constructing a stable GSN knockdown cell line model for subsequent experiments. In addition, we verified the knockdown efficiency of three different si‐*Gsn* sequences and found that the first si‐*Gsn* exhibited the most prominent knockdown effect, with more than 70% reduction at both transcription and protein levels (Figure S5H–J, Supporting Information). Subsequently, we constructed a stable *Gsn* overexpressing hepatocyte cell line by infecting AML12 cells with lentivirus carrying the PLVX‐FLAG‐*Gsn* plasmid (Figure S5K–M, Supporting Information).

Based on our previous results, we selected AML12 cells as our research model, using 0.3 mmol of PA as the working concentration and a 24‐h incubation time to simulate the metabolic stress environment of MASH in hepatocytes in vitro, with an equal concentration of bovine serum albumin (BSA) as the control. Oil Red O staining results revealed a significant increase in lipid accumulation in sg‐*Gsn*‐treated hepatocytes (Figure 4E and Figure S6A, Supporting Information), while GSN overexpressing hepatocytes showed a marked reduction in lipid accumulation (Figure 4F and Figure S6B, Supporting Information). Furthermore, total cellular triglyceride measurements corroborated these findings (Figure 4G,H).

WB and qPCR analyses confirmed the changes in gene and protein expression related to lipid metabolism. Specifically, sg‐*Gsn* cells exhibited higher levels of genes and proteins associated with lipid accumulation (Figure 4I,K and Figure S6C, Supporting Information), while GSN overexpressing cells showed a downregulation of these markers (Figure 4J,L and Figure S6D, Supporting Information). Moreover, ELISA results showed that the expression of inflammatory cytokines was significantly elevated in sg‐*Gsn* hepatocytes (Figure 4M). In GSN overexpressing cells, the levels of these inflammatory cytokines were reduced, although not as dramatically as in the knockdown group (Figure 4N).

To further validate these findings, we conducted additional experiments in AML12 cells. First, stable *Gsn* knockout AML12 cells transduced with sg‐*Gsn* lentivirus and their control cells (sg‐Ctrl AML12) were transfected with either an empty vector or a GSN overexpression plasmid. Western blot analysis confirmed the stable overexpression of GSN (Figure S7D,E, Supporting Information). The cells were then treated with BSA or PA at the previously described concentrations for 24 h. Oil Red O staining results demonstrated that the lipid accumulation induced by *Gsn* knockout in AML12 cells was significantly alleviated by the restoration of GSN expression (Figure S7A,B, Supporting Information). ELISA analysis of inflammatory factors in cell culture supernatants confirmed that GSN restoration in *Gsn* knockout AML12 cells not only reduced lipid accumulation but also alleviated hepatocyte inflammation (Figure S7C, Supporting Information). Western blot (Figure S7D,E, Supporting Information) and q‐PCR (Figure S7F, Supporting Information) analyses further showed increased expression of lipid metabolism‐related molecules in *Gsn* knockout cells upon PA treatment. However, these levels were markedly reduced at both the transcriptional and protein levels following GSN restoration. Collectively, these in vitro findings further corroborated and supplemented the conclusions derived from the in vivo experimental models.

In contrast, in GSN overexpressing AML12 cells, the introduction of si‐*Gsn* led to opposite effects. We observed that in GSN overexpressing AML12 cells, si‐*Gsn* reversed the reduction in lipid metabolism‐related molecules previously caused by the increase in GSN expression (Figure S8D–F, Supporting Information). Oil Red O staining and quantitative results also confirmed these observations (Figure S8A,B, Supporting Information), and the levels of inflammatory cytokines in the cell supernatant exhibited a corresponding trend (Figure S8C, Supporting Information).

In primary hepatocytes, GSN expression also exhibited a concentration‐ and time‐dependent upregulation upon PA stimulation, with maximal induction observed at 0.2 × 10^−3^ m PA for 24 h (Figure S10A–D, Supporting Information). GSN deficiency aggravated PA‐triggered inflammatory responses, with heightened secretion of TNF‐α, IL‐6, and IL‐1β (Figure S9E, Supporting Information), coupled with increased lipid droplet formation (Figure S10F,H, Supporting Information) and elevated triglyceride (TG) content (Figure S10G, Supporting Information). Both WB and qPCR analyses revealed consistent patterns of gene and protein expression related to lipid metabolism (Figure S10I–K, Supporting Information). These findings in primary hepatocytes align with AML12 cell data, confirming GSN's dual role in mitigating lipotoxicity through coordinated regulation of lipid metabolism, highlighting its adaptive function against metabolic stress.

In conclusion, these experimental results provide strong evidence supporting the critical role of GSN in regulating lipid metabolism and reducing inflammation in an in vitro model that simulates the metabolic stress environment induced by MASH. The ability of GSN to modulate lipid metabolism and inflammatory responses underscores its potential therapeutic value in mitigating the pathological effects of metabolic liver diseases, particularly in the context of MASH.

### 2.5 GSN Deficiency‐Induced F‐Actin Depolymerization Abnormalities Lead to YAP Hyperactivation and Exacerbate Liver Fibrosis and Inflammation in MASH

GSN is a cytoskeletal protein crucial for maintaining cellular morphology and function through its regulation of the dynamic remodeling of F‐actin filaments. The activation of GSN influences the polymerization and depolymerization of F‐actin within cells, which in turn affects cell morphology and signal transduction.^[^ ^22^ ^]^ Recent studies have highlighted that the dynamic remodeling of F‐actin regulates the activation of YAP, a key transcriptional coactivator involved in various biological processes, including its pivotal role in the progression of MASH.^[^ ^23^, ^24^ ^]^ Therefore, we hypothesize that GSN activation promotes F‐actin depolymerization, thereby inhibiting YAP nuclear translocation and activation, ultimately affecting cell proliferation and transcriptional regulation.^[^ ^25^, ^26^ ^]^

**Figure 5:**
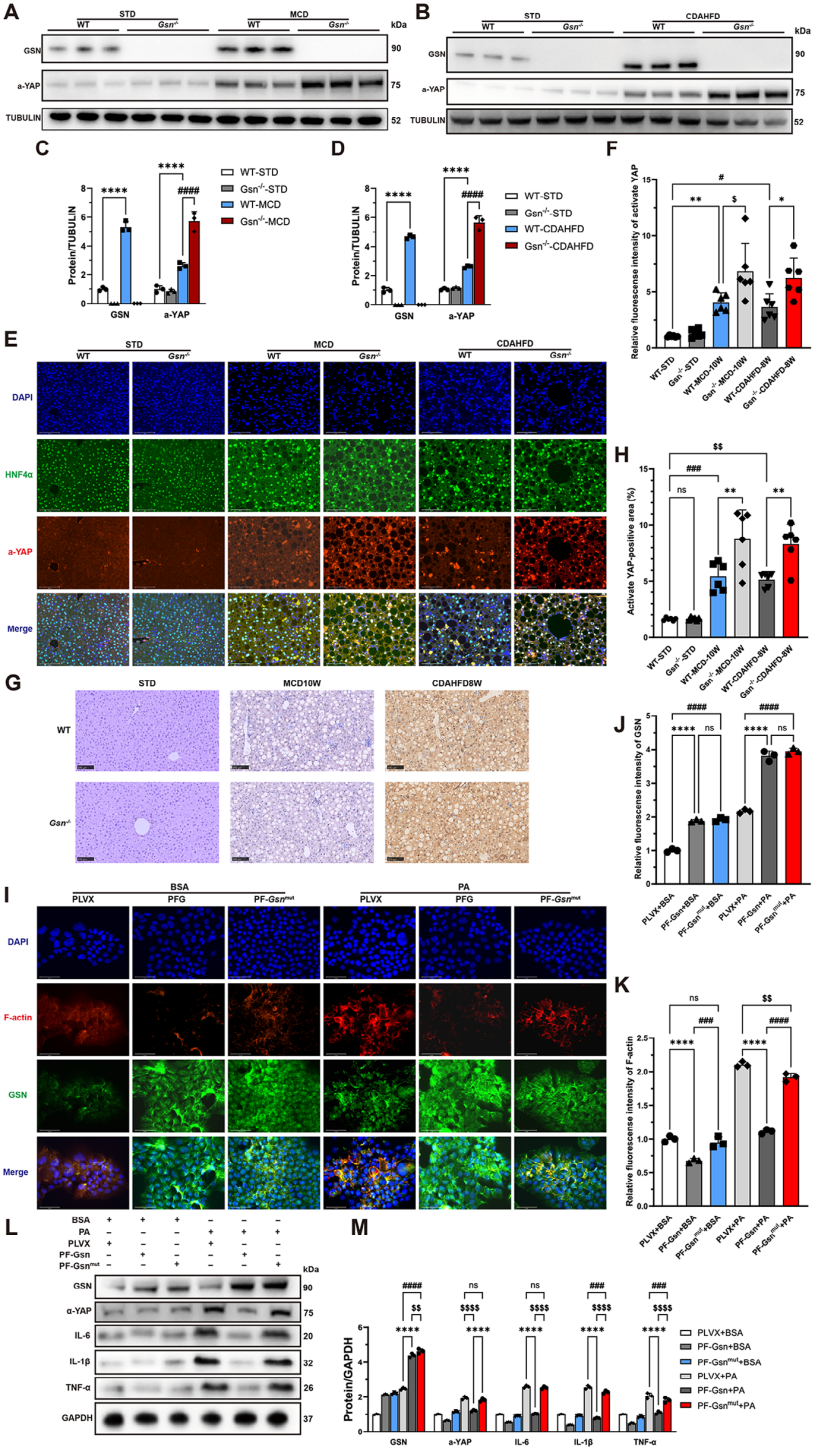
*Gsn* deficiency‐induced F‐actin depolymerization abnormalities lead to YAP hyperactivation and exacerbate liver fibrosis and inflammation in MASH. B) Western blots results of the expression of activate‐YAP in in liver tissues of WT/*Gsn* ^−/−^ mice fed with MCD or CDAHFD diets, with mice on a standard diet (STD) serving as the control group for each dietary intervention (*n* = 3/group); C,D) quantitative data of Western blot results from panels (A) and (B); representative immunofluorescence images of liver tissues from mice fed with MCD or CDAHFD diets, with mice on a STD serving as the control group (*n* = 6/group, Red: activated‐YAP, Green: HNF4α, Blue: DAPI); quantitative analysis of immunofluorescence staining from panel (E); (G) representative immunohistochemical staining images of activated‐YAP from mouse liver tissues of mice fed with MCD or CDAHFD diets, with mice on a STD serving as the control group (*n* = 6/group). H) Quantitative analysis of IHC staining from panel (G). I) Representative immunofluorescence staining images of GSN and F‐actin in AML12 cells treated with either BSA or PA across different experimental groups (*n* = 3/group); J) quantitative analysis of immunofluorescence staining of GSN from panel (I); K) quantitative analysis of immunofluorescence staining of F‐actin from panel (I); the expression levels of GSN, activate‐YAP, IL‐1β, TNF‐α, and IL‐6 in AML12 cells treated with either BSA or PA across different experimental groups (*n* = 3/group); quantitative data of Western blot results from panel (L). Data were expressed as the means ± standard deviation (SD). Significant differences were determined using one‐way ANOVA as appropriate. A two‐tailed *p* < 0.05 was considered statistically significant (*p < 0.05, **p < 0.01, ***p < 0.005, ****p < 0.001, n.s., no significance).

To test this hypothesis, we first conducted WB experiments. Here, we used a specific antibody targeting activated YAP (ab205270), many previous studies have also confirmed the reliability of this antibody for detecting activated YAP.^[^ ^27^, ^28^, ^29^ ^]^ We observed a significant upregulation of activated YAP(a‐YAP) expression in liver tissues from *Gsn* knockout (*Gsn* ^−/−^) mice fed MCD and CDAHFD diets (**Figure** **5**A–D). Immunofluorescence staining and IHC analysis of liver tissues from MASH model mice further revealed prominent YAP nuclear localization (Figure 5E–H), indicating that the loss of GSN expression leads to significant YAP activation.

In addition, in AML12 cells treated with PA and BSA, we observed a significant increase in activate YAP protein levels in sg‐*Gsn* AML12 cells following PA induction (Figure S9A,B, Supporting Information). In contrast, GSN overexpression leads to a decrease in the protein expression of activate YAP (Figure S9C,D, Supporting Information). This finding suggests that GSN plays an essential role in regulating YAP activation in vitro.

To further investigate GSN's role in cytoskeletal regulation, we constructed a *Gsn* mutant plasmid that lacked the ability to regulate F‐actin. We transfected this mutant plasmid, a wild‐type *Gsn* overexpression plasmid, and a PLVX control plasmid into AML12 cells to create stable transfected cell lines. Immunofluorescence staining revealed that, compared to the BSA treatment group, PA treatment led to increased F‐actin accumulation in AML12 cells, while GSN overexpression significantly reduced this accumulation. However, overexpression of the *Gsn* mutant plasmid failed to reverse F‐actin accumulation (Figure 5I–K). WB analysis also showed that, compared to the PLVX group, activate YAP levels were significantly downregulated in GSN‐overexpressing cell lines, but this downregulation was absent in cells overexpressing the *Gsn* mutant (Figure 5L,M). ELISA experiments were conducted to assess the levels of inflammatory cytokines in the culture supernatants of AML12 cells treated with different conditions. The results revealed that overexpression of GSN in AML12 cells led to a reduction in inflammatory cytokine levels, whereas overexpression of the mutant form of GSN failed to exhibit this effect (Figure S11A–C, Supporting Information). These findings suggest that GSN, as an important cytoskeletal protein, plays a critical role in preventing excessive accumulation of F‐actin, thereby averting the hyperactivation of proteins such as YAP and the subsequent damage to liver tissue.

### 2.6 Cytochalasin B Reversed the Enhancement of YAP Activation Caused by GSN Deficiency

**Figure 6:**
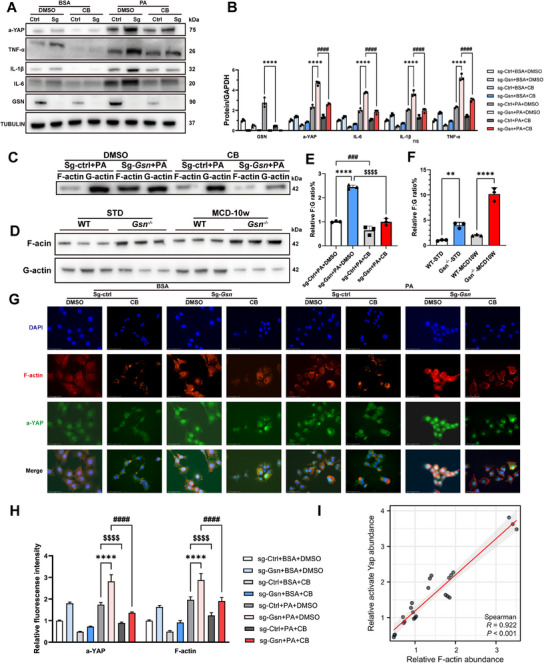
Cytochalasin B reversed the enhancement of YAP activation caused by *Gsn* deficiency. A) The protein expression levels of GSN, activate‐YAP, TNF‐α, IL‐1β, IL‐6 in AML12 cells treated with either BSA or PA across different experimental groups (*n* = 3/group); B) quantitative data of Western blot results from panel (A); C–F) the ratio of F‐actin to G‐actin was measured in cells subjected to various treatments and different groups of mouse liver tissues; G) representative immunofluorescence staining images of a‐YAP and F‐actin in AML12 cells treated with either BSA or PA across different experimental groups (*n* = 3/group); H) quantitative analysis of immunofluorescence staining from panel (G); I) correlation analysis of activate‐YAP and F‐actin in AML12 cells. Data were expressed as the means ± standard deviation (SD). Significant differences were determined using one‐way ANOVA as appropriate. A two‐tailed *p* < 0.05 was considered statistically significant (*p < 0.05, **p < 0.01, ***p < 0.005, ****p < 0.001, n.s., no significance).

Next, we treated sg‐ctrl and sg‐*Gsn* AML12 cells with cytochalasin B (CB), a drug that inhibits F‐actin polymerization and promotes depolymerization. Cells were also treated with PA to simulate a metabolic environment conducive to lipid accumulation. CB treatment significantly disrupted F‐actin accumulation and YAP activation induced by *Gsn* knockout, leading to a reduction in inflammatory factor expression, as confirmed by western blots and ELISA and experiments (**Figures** **6**A,B and S11D–F, Supporting Information).

Subsequently, AML12 cells (sg‐Ctrl, sg‐*Gsn*) were treated with PA for 12 h, followed by the addition of an appropriate concentration of cytochalasin B or DMSO for an additional 12 h. Total cellular proteins were then extracted, and F‐actin and G‐actin were separated as described in the methodology, followed by Western blot analysis (Figure 6C,E). We observed that under PA treatment, *Gsn* knockout significantly increased the F‐actin/G‐actin ratio, which was markedly reduced upon cytochalasin B treatment. In contrast, this difference was less pronounced in the control group. These findings indicate that *Gsn* knockout induces abnormal degradation of F‐actin, leading to its aberrant accumulation, a phenomenon that can be mitigated by the F‐actin‐specific depolymerizing agent CB. To further validate these findings, we extracted liver tissues from mice and used a kit to isolate F‐actin and G‐actin from liver tissue. Immunoblotting showed that the F‐actin to G‐actin ratio was highest in the liver tissues of *Gsn* ^−/−^ mice on the MCD diet (Figure 6D,F), indicating that F‐actin is activated and accumulated in GSN‐deficient livers under MASH diet conditions.

Subsequent multiplex immunofluorescence staining confirmed that *Gsn* knockout led to hyperactivation of F‐actin and significantly increased YAP nuclear translocation, and the addition of cytochalasin B markedly disrupted these effects (Figure 6G,H). Finally, we performed a quantitative analysis of fluorescence intensity and correlation analysis, which revealed a strong positive correlation between YAP activation levels and F‐actin fluorescence intensity (Figure 6I). This result is consistent with prior studies and further confirms our experimental conclusions.

### 2.7 In Vivo Verteporfin Injection Alleviates Inflammation and Fibrosis in the Liver Tissues of MASH‐Model *Gsn* ^−/−^ Mice

To determine whether YAP activation is a downstream effect of F‐actin accumulation caused by GSN deficiency, we performed a series of targeted experiments. *Gsn* ^−/−^ mice were treated with verteporfin (100 mg kg^−1^), a YAP inhibitor, or corn oil (10 mL kg^−1^) every two days for 14 d, followed by an additional four weeks of MCD diet (Figure S12A, Supporting Information). After the four‐week treatment period, we analyzed liver tissues from the different treatment groups. Our results showed that verteporfin treatment significantly reduced activate YAP expression in *Gsn* ^−/−^ mouse liver tissues (Figure S12C,F, Supporting Information). This activate YAP suppression was associated with a marked alleviation of fibrosis and inflammation, as indicated by relevant biomarkers (Figure S12B–D, Supporting Information). These findings strongly suggest that YAP activation in GSN‐deficient liver tissue is a critical factor driving liver fibrosis and inflammation, independent of hepatic steatosis. Serum measurements of ALT and AST demonstrated that inhibition of YAP activation alleviated liver damage in MASH mice (Figure S12G,H, Supporting Information). In addition, hepatic hydroxyproline assays confirmed that suppressing YAP activation mitigated the degree of liver fibrosis during the progression of MASH (Figure S12I, Supporting Information). Finally, ELISA results revealed a reduction in serum inflammatory cytokine levels in mice following decreased YAP activation (Figure S12J–L, Supporting Information).

In summary, these findings demonstrate that GSN regulates YAP activation through its influence on F‐actin dynamics. In the absence of GSN, F‐actin accumulation leads to YAP activation, driving severe inflammation and fibrosis in liver tissues. GSN activation promotes F‐actin depolymerization, thereby inhibiting YAP nuclear translocation and activation, which in turn affects cell proliferation and transcriptional regulation. These results underscore the critical role of GSN in maintaining cellular functions and suggest its potential as a therapeutic target for MASH treatment.

### 2.8 GSN and MDM2 are Co‐Expressed and Facilitate the Ubiquitination of P53

**Figure 7:**
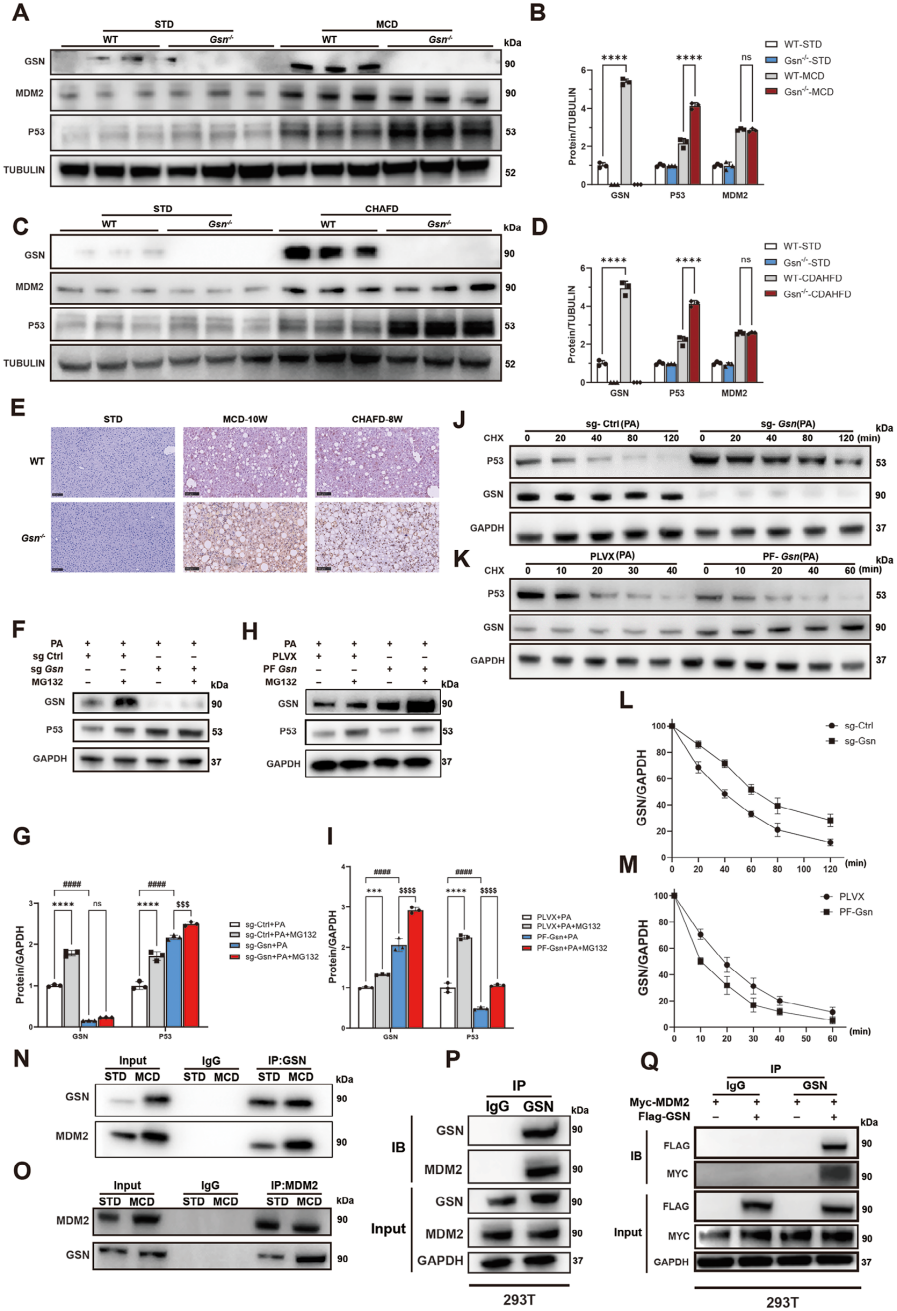
GSN and MDM2 are co‐expressed and facilitate the ubiquitination of P53. A–C) Western blots results of the expression of P53 and MDM2 in liver tissues of WT/*Gsn* ^−/−^ mice fed with MCD or CDAHFD diets, with mice on a standard diet (STD) serving as the control group for each dietary intervention (*n* = 3/group); E) representative immunohistochemical (IHC) staining images of P53 in liver tissues of mice fed with MCD or CDAHFD diets, with mice on a STD serving as the control group (*n* = 6/group). F–I) Western blot analysis showing GSN and P53 protein levels, with GAPDH as a loading control. F,G) Cells under conditions: palmitic acid (PA), control sgRNA (sg‐Ctrl), *Gsn* sgRNA (sg‐*Gsn*), and MG132. H,I) Cells under conditions: PA, PLVX vector, *Gsn* overexpression (PF‐*Gsn*), and MG132. J–M) Cycloheximide (CHX) chase assay showing the stability of P53 and GSN proteins over time, with GAPDH as a loading control. J,L) Cells with control sgRNA (sg‐Ctrl) and *Gsn* sgRNA (sg‐*Gsn*) at various time points (0, 20, 40, 80, 120 min). K,M) Cells with PLVX vector and *Gsn* overexpression (PF‐*Gsn*) at various time points (0, 10, 20, 30, 40, 60 min). O) Western blot analysis showing the effects of various treatments on P53, MDM2, and GSN protein levels, with GAPDH as a loading control. Cells were treated with palmitic acid (PA), PLVX vector, *Gsn* overexpression (PF‐*Gsn*), control siRNA (si‐Ctrl), and *Mdm2* siRNA (si‐*Mdm2*). P,Q) Co‐immunoprecipitation analysis demonstrating the interaction between GSN and MDM2 under STD and MCD treatment conditions. P) Immunoprecipitation with anti‐GSN antibody showing associated MDM2 levels. Q) Immunoprecipitation with anti‐MDM2 antibody showing associated GSN levels. Input and IgG controls are included for comparison. N,O) In vivo immunoprecipitation experiments revealed co‐expression of MDM2 and GSN. Input and IgG controls are included for comparison. S) In vitro immunoprecipitation assays conducted in 293T cells observed co‐expression of MDM2 and GSN. Input samples demonstrate protein expression levels, with GAPDH as a loading control. Data were expressed as the means ± standard deviation (SD). Significant differences were determined using one‐way ANOVA as appropriate. A two‐tailed *p* < 0.05 was considered statistically significant (*p < 0.05, **p < 0.01, ***p < 0.005, ****p < 0.001, n.s., no significance).

Notably, lipid accumulation in the liver was significantly increased in GSN‐deficient (*Gsn* ^−/−^) mice. Since YAP showed no significant effect on hepatic steatosis during MASH progression, this suggests the involvement of other downstream effectors. Previous studies reported that GSN binds to P53 in HepG2 cells, inhibiting its nuclear translocation and suppressing its activity.^[^ ^30^ ^]^ In addition, increased hepatic P53 expression has been linked to exacerbated steatosis in the liver, prompting us to focus on P53 in subsequent experiments. We observed a significant increase in total P53 protein levels in the liver tissues of *Gsn* ^−/−^ mice under MCD and CDAHFD conditions (**Figure** **7**A–D), despite no differences at the transcriptional level (Figure S13A, Supporting Information). The IHC staining results for P53 were consistent with those observed in the western blot analysis (Figure 7 and Figure S13B, Supporting Information). In the livers of mice fed two types of MASH diets, *Gsn* knockout not only led to an increase in P53‐positive staining areas but also revealed a pronounced nuclear translocation of P53 in hepatocytes. This observation aligns with findings reported in previous studies. These findings support our hypothesis that P53 may act as another downstream target of GSN, influencing hepatic steatosis during MASH progression.

To further investigate how GSN affects P53 protein levels, we conducted experiments in the AML12 cell line. Results showed that neither GSN knockdown nor overexpression altered P53 mRNA levels (Figure S13C,D, Supporting Information). However, *Gsn* knockout upregulated P53 protein expression (Figure S9A,B, Supporting Information), whereas GSN overexpression suppressed it (Figure S9C,D, Supporting Information). These cellular results were consistent with the in vivo findings. Since P53 transcription levels remained unchanged, while changes in GSN expression significantly impacted P53 protein levels, we hypothesized that GSN may regulate P53 ubiquitination, leading to discrepancies between its transcriptional and protein expression levels. To investigate this, subsequent experiments showed that treating cells with the proteasome inhibitor MG132 slowed P53 degradation, indicating that GSN may reduce P53 stability via the proteasomal pathway (Figure 7F–I). We further treated AML12 cells with cycloheximide (CHX), a protein synthesis inhibitor commonly used to study protein stability and degradation mechanisms. Under PA treatment, P53 stability significantly increased in sg‐*Gsn* AML12 cells (Figure 7J,L), while the opposite effect was observed in *Gsn*‐overexpressing cells (Figure 7K,M). These results suggest that GSN influences P53 stability, playing a critical role in MASH progression.

By using the protein interaction database BioGRID,^[^ ^31^ ^]^ we identified a potential interaction between GSN and the E3 ubiquitin ligase MDM2. MDM2 is known to regulate P53 protein levels by mediating its ubiquitination and degradation.^[^ ^32^, ^33^ ^]^ We hypothesized that GSN enhances MDM2‐P53 interactions, promoting P53 ubiquitination and subsequent degradation.

To validate this hypothesis, we used siRNA to suppress MDM2 expression. siRNA screening results in AML12 cells were conducted (Figure S14A–C, Supporting Information). The results showed that inhibiting MDM2 expression abolished the suppressive effect of GSN overexpression on P53 protein levels (Figure S14D,E, Supporting Information). Meanwhile, we also found that GSN overexpression had no effect on the protein expression levels of MDM2 (Figure S14D, Supporting Information), a finding that was validated at both the cellular (Figure S9, Supporting Information) and tissue levels (Figure 7A–D). This finding confirmed that GSN modulates P53 degradation via the MDM2‐mediated pathway.

To investigate whether GSN directly interacts with MDM2, we performed co‐immunoprecipitation (Co‐IP) assays and detected a stable GSN‐MDM2 protein complex in both in vivo (Figure 7N,O) and in vitro experiments (Figure 7P,Q). Moreover, the interaction between endogenous GSN and MDM2 was increased in MCD‐fed mice compared to those on a standard diet (Figure 7N,O).

### 2.9 GSN Enhances MDM2‐Mediated P53 Ubiquitination Thereby Reducing Hepatic Steatosis

**Figure 8:**
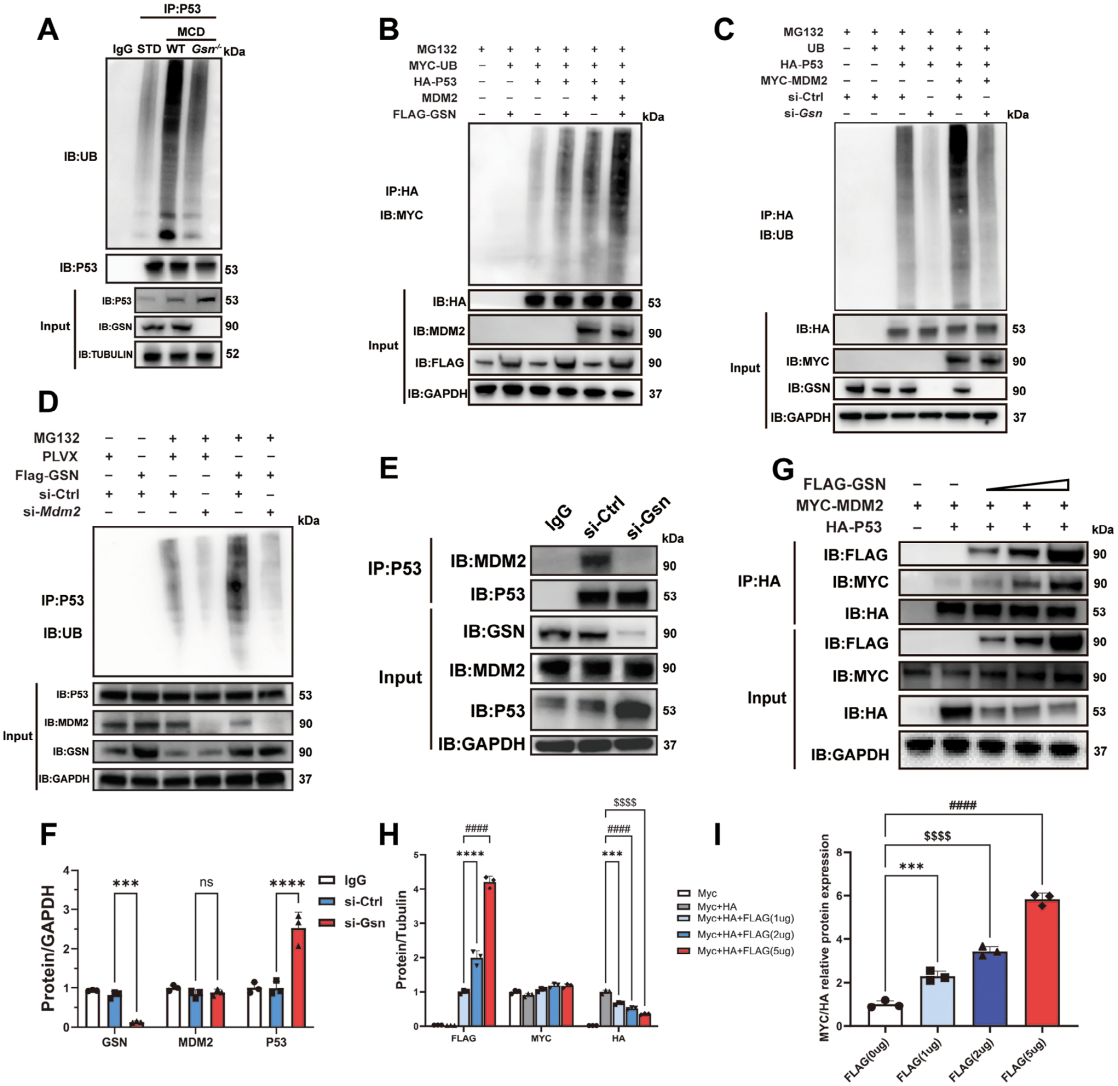
GSN enhances MDM2‐mediated P53 ubiquitination thereby reducing hepatic steatosis. A) In vivo immunoprecipitation (IP) and ubiquitination assays of P53 in liver tissues of WT/*Gsn* ^−/−^ mice fed with MCD or CDAHFD diets, with mice on a standard diet (STD) serving as the control group for each dietary intervention (*n* = 3/group); B,C) the immunoprecipitation and ubiquitination assays of P53 in 293T cells with *Gsn* overexpression or knockdown. Cells were treated with MG132 to inhibit proteasomal degradation. IP was performed using HA‐tagged P53, followed by immunoblotting (IB) for MYC‐tagged ubiquitin (MYC‐UB) or ubiquitin. D) The immunoprecipitation and ubiquitination assays of P53 in 293T cells with MDM2 knockdown. Cells were treated with MG132 to inhibit proteasomal degradation. IP was performed using P53, followed by IB for ubiquitin. E,F) IP of P53 followed by IB to detect associated proteins MDM2 and GSN. G–I) Analysis of the effect of increasing FLAG‐GSN expression on the interaction between MYC‐MDM2 and HA‐P53. IP was performed for P53, with IB detecting FLAG, MYC, and HA tags. Data were expressed as the means ± standard deviation (SD). Significant differences were determined using one‐way ANOVA as appropriate. A two‐tailed *p* < 0.05 was considered statistically significant (*p < 0.05, **p < 0.01, ***p < 0.005, ****p < 0.001, n.s., no significance).

MDM2 is a well‐known E3 ubiquitin ligase that plays a critical role in the ubiquitination and degradation of P53. Based on the results above, we hypothesize that GSN may interact with MDM2 to enhance its ubiquitination effects on P53, leading to the cytoplasmic retention and ubiquitin‐mediated degradation of P53, thereby preventing its nuclear translocation and functional activation. To validate this hypothesis, we then examined the effect of GSN on MDM2‐driven P53 ubiquitination. Immunoprecipitation experiments demonstrated a marked reduction in P53 ubiquitination in the livers of *Gsn* ^−/−^ mice under MCD conditions (**Figure** **8**A), indicating impaired P53 ubiquitination in the absence of GSN in vivo, leading to its stabilization and accumulation. In 293T cells co‐expressing MDM2 and P53, strong ubiquitinated P53 (Ub‐P53) bands were observed. However, these bands were significantly enhanced in GSN‐overexpressed cells (Figure 8B). Conversely, GSN knockdown attenuated P53 ubiquitination (Figure 8C). Notably, siRNA‐mediated MDM2 suppression in 293T cells abolished the GSN overexpression‐induced increase in endogenous P53 ubiquitination (Figure 8D). In addition, GSN knockdown reduced MDM2‐P53 interactions without affecting MDM2 expression levels (Figure 8E,F). Quantitative IP analysis revealed that GSN promoted MDM2‐P53 interactions in a dose‐dependent manner (Figure 8G–I). These findings indicate that GSN facilitates MDM2‐driven P53 ubiquitination and degradation by interacting with MDM2.

### 2.10 Inhibition of P53 Slowed the Process of Lipid Accumulation in Hepatocytes

To confirm that reduced P53 ubiquitination and its abnormal accumulation contribute to GSN deficiency‐induced hepatic steatosis, we treated *Gsn* ^−/−^mice with the P53 inhibitor PFT via intraperitoneal injection (Figure S15A, Supporting Information). IHC (Figure S15D,E, Supporting Information) and WB (Figure S15F,G, Supporting Information) analyses revealed that PFT treatment significantly suppressed elevated P53 protein levels in *Gsn* ^−/−^ mice. As predicted, P53 inhibition significantly ameliorated hepatic steatosis in *Gsn* ^−/−^ mice. Hepatic triglyceride levels significantly declined following P53 inhibition, further supporting these observations (Figure S15B, Supporting Information). Histological analyses showed a marked reduction in lipid droplet vacuoles in HE and Oil Red O staining, indicating decreased intracellular lipid accumulation (Figure S15C, Supporting Information). The expression of various lipid metabolism‐related molecules decreased significantly at both protein levels (Figure S15F,G, Supporting Information) and transcriptional (Figure S15H, Supporting Information) after P53 inhibition.

These results suggest that reduced P53 ubiquitination and its accumulation may be a key factor in GSN deficiency‐induced hepatic steatosis. Similar findings were observed in vitro, where PFT inhibition of P53 expression in AML12 cells reduced lipid accumulation in sg‐*Gsn* cells under both BSA and PA conditions, as confirmed by WB (Figure S165A,B, Supporting Information), cellular TG analyses (Figure S16C, Supporting Information) and q‐PCR (Figure S16D, Supporting Information) analyses. This comprehensive study underscores the role of P53 in GSN deficiency‐induced hepatic steatosis.

In summary, our study highlights the critical role of GSN in regulating P53 through MDM2‐mediated ubiquitination and degradation. GSN promotes MDM2‐P53 interactions, enhancing P53 ubiquitination and degradation. This mechanism explains the elevated P53 protein levels observed in GSN‐deficient mice, contributing to hepatic steatosis and MASH progression. Understanding this pathway provides valuable insights into potential therapeutic targets for MASH and related metabolic disorders.

### 2.11 GSN Expression Is Upregulated by the Transcription Factor ATF3 during MASH Progression

Next, we explored the potential molecular mechanisms underlying the upregulation of GSN in MASH. A review of prior studies revealed that activating transcription factor 3 (ATF3) was identified as an upstream transcriptional regulator of GSN in bladder cancer, modulating its transcription.^[^ ^34^ ^]^ ATF3 is a critical transcription factor involved in liver metabolic diseases. It is induced by oxidative stress and inflammation and regulates immune responses by modulating Toll‐like receptor (TLR) signaling in macrophages.^[^ ^35^, ^36^ ^]^ In liver diseases like MAFLD and MASH, ATF3 expression is upregulated, contributing to hepatic steatosis, insulin resistance, and the progression to type 2 diabetes (T2D).^[^ ^37^ ^]^ ATF3 regulates key molecules such as FOXO1 and CD36, which are essential for lipid metabolism and glucose homeostasis.^[^ ^37^ ^]^ Furthermore, ATF3 promotes a shift in hepatocyte death from apoptosis to necroptosis, particularly under conditions of severe hepatic steatosis. This shift is mediated by the induction of RIPK3, a necroptosis regulator, contributing to liver injury and fibrosis.^[^ ^38^ ^]^ As a modulator of lipid metabolism and liver injury ATF3 has emerged as a potential therapeutic target for managing liver diseases like MASH, MAFLD, and T2D.

**Figure 9:**
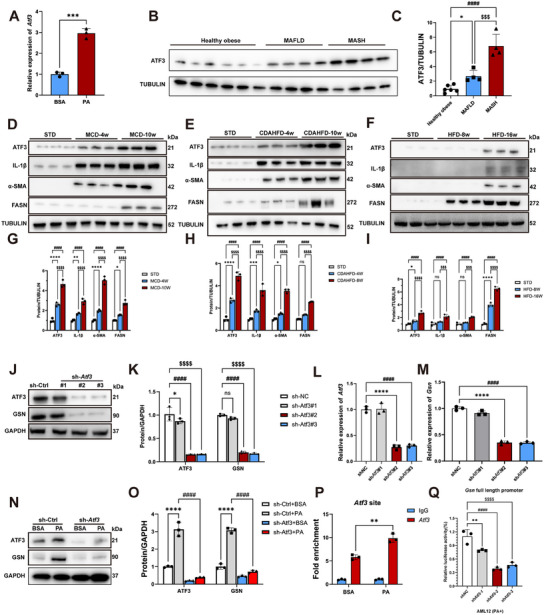
GSN expression is upregulated by the transcription factor ATF3 during MASH progression. A) Q‐PCR analysis of mRNA expression of *Atf3* in AML12 cells subjected to BSA or PA for 24 h; B) Western blot analysis was performed to assess ATF3 protein expression in human liver tissue samples (*n* = 4–6/group); C) quantitative data of Western blot results from panel (B); D–I) the expression levels and quantitative data of ATF3 and other molecules in liver tissues were evaluated at different time points in mice fed with the methionine and choline‐deficient diet (MCD, D,G), the choline‐deficient, l‐amino acid‐defined, high‐fat diet (CDAHFD, H), and the high‐fat diet (HFD, I) using Western blot analysis, with mice on a standard diet (STD) serving as the control group for each dietary intervention (*n* = 3/group); J) Western blot analysis showing ATF3 and GSN protein levels, with GAPDH as a loading control. Cells under conditions: control shRNA (sh‐Ctrl), *Atf3*‐shRNA#1 (sh‐*Atf3#1*), *Atf3*‐shRNA#2 (sh‐*Atf3#2*), *Atf3*‐shRNA#3 (sh‐*Atf3#3*); K) quantitative data of Western blot results from panel (J); L,M) Q‐PCR analysis showing *Atf3*(L) and *Gsn*(M) transcription levels. Cells under conditions: control shRNA (sh‐Ctrl), *Atf3*‐shRNA#1 (sh‐*Atf3#1*), *Atf3*‐shRNA#2 (sh‐*Atf3#2*), *Atf3*‐shRNA#3 (sh‐*Atf3#3*); N) Western blot analysis showing ATF3 and GSN protein levels, with GAPDH as a loading control. Cells under conditions: BSA or PA, control shRNA (sh‐Ctrl), *Atf3*‐shRNA(sh‐*Atf3*); O) quantitative data of Western blot results from panel (N); P) ChIP analysis of the enrichment of ATF3 protein in the *Gsn* promoter in AML12 cells subjected to BSA or PA for 24 h. IgG was the negative control; Q) dual luciferase reporter assay of *Gsn* promoter activity in shNC or sh*Atf3*‐infected AML12 cells subjected to PA for 24 h. Luciferase activity was normalized with the internal Renilla luciferase control (*n* = 3). Data were expressed as the means ± standard deviation (SD). Significant differences were determined using one‐way ANOVA as appropriate. A two‐tailed *p* < 0.05 was considered statistically significant (*p < 0.05, **p < 0.01, ***p < 0.005, ****p < 0.001, n.s., no significance).

Our initial experiments revealed that PA stimulation significantly upregulated Atf3 expression at the transcriptional level in AML12 cells (**Figure** **9**A). Western blot analysis of human liver tissue samples further showed a progressive increase in ATF3 protein expression across groups: from healthy obese individuals to those with metabolic‐associated fatty liver disease (MAFLD), and finally to MASH patients (Figure 9B,C). Specifically, ATF3 protein levels were nearly threefold higher in MASH patients compared to healthy obese individuals (Figure 9B,C). Similarly, in three MASH mouse models, hepatic ATF3 protein expression was markedly elevated in mice fed MCD and CDAHFD compared to those on a HFD (Figure 9D–I). Consistent with our earlier findings, inflammation‐ and fibrosis‐related molecular markers were less prominently upregulated in HFD‐fed mice compared to the MCD and CDAHFD groups (Figure S1, Supporting Information). These findings are consistent with the previously observed expression pattern of GSN in MASH, suggesting a strong correlation between the expression of both factors during the progression of MASH.

To further validate the role of ATF3 in regulating GSN expression, we used shRNA to successfully knock down ATF3 expression in AML12 cells (Figure 9J,K). Both Western blot (Figure 9J,K) and q‐PCR (Figure 9L,M) analyses confirmed that reduced ATF3 expression significantly decreased GSN levels, supporting our hypothesis. Next, sh‐Ctrl AML12 cells and sh‐*Atf3* AML12 cells were cultured with either BSA or PA. The results showed that PA treatment induced an increase in the protein expression levels of GSN and ATF3. Notably, knockdown of ATF3 resulted in a reduction of GSN expression under both BSA and PA culture conditions (Figure 9N,O).

**Figure 10:**
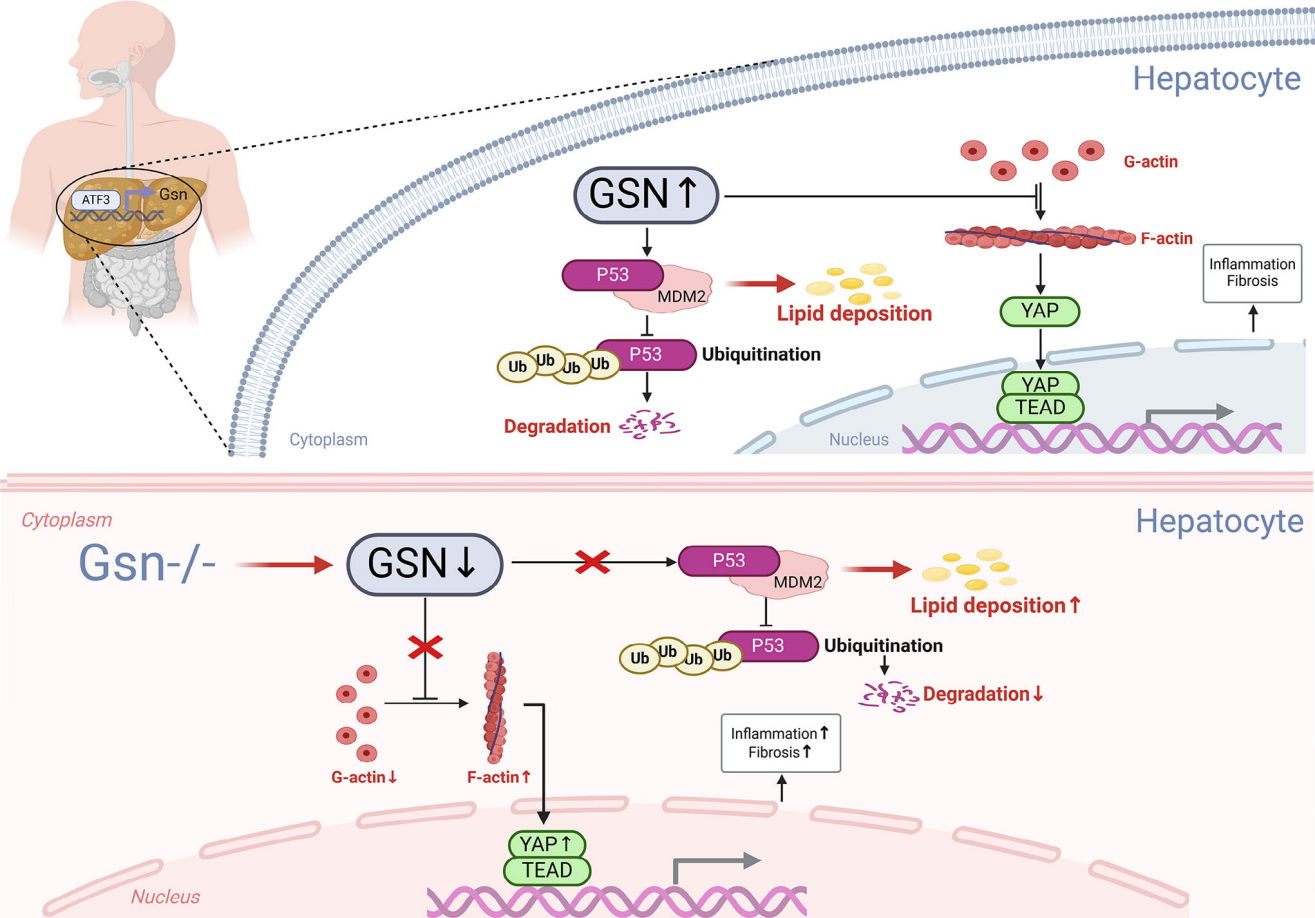
Schematic overview of the mechanistic pathways investigated in this study. GSN promotes the ubiquitination and degradation of P53, thereby inhibiting lipid deposition. Under normal GSN levels, GSN interacts with MDM2 in the liver to enhance MDM2‐mediated P53 ubiquitination and degradation, leading to reduced P53 expression and preventing its effective nuclear translocation. This impacts liver lipid metabolism during MASH progression, reducing lipid accumulation and alleviating hepatic steatosis. However, in the absence of GSN (*Gsn* ^−/−^), the MDM2‐mediated P53 degradation is impaired, leading to increased lipid deposition and exacerbating hepatic steatosis and metabolic dysfunction. In addition to regulating P53, GSN functions as a key cytoskeletal protein by dynamically adjusting the ratio of F‐actin to G‐actin, ensuring excess F‐actin is degraded into G‐actin. This prevents excessive F‐actin accumulation, maintaining cellular stability. This regulation of F‐actin indirectly inhibits overactivation of YAP, a key effector of the Hippo pathway. In *Gsn* ^−/−^ hepatocytes, the imbalance between F‐actin and G‐actin prevents the timely disassembly of excess F‐actin into G‐actin monomers. Pathological accumulation of F‐actin results in YAP increased activation before regulation by the Hippo pathway. This contributes to enhanced liver fibrosis and activation of inflammatory signaling pathways during MASH progression, further aggravating liver damage. By regulating F‐actin, YAP activity, and promoting P53 degradation through MDM2, GSN plays a central role in maintaining liver metabolic homeostasis, regulating lipid metabolism, and controlling inflammation. These findings underscore the multifaceted roles of GSN in liver function and offer potential therapeutic targets for MASH treatment.

Next, we identified potential Atf3‐binding sites within the *Gsn* promoter region and performed chromatin immunoprecipitation (ChIP) assays to confirm *Atf3*’s transcriptional activation of *Gsn* under PA stimulation. ChIP‐qPCR results demonstrated a significant increase in Atf3 binding to the *Gsn* promoter in PA‐treated AML12 cells, providing strong evidence that PA induces *Gsn* expression through Atf3 (Figure 9P). To further confirm this mechanism, we constructed a *Gsn*‐responsive luciferase reporter system to evaluate the effect of *Atf3* knockdown on *Gsn* promoter activity. Silencing *Atf3* effectively suppressed the activity of the pGL3‐basic‐*Gsn* promoter, indicating that *Atf3* directly regulates *Gsn* transcription through promoter interaction (Figure 9Q). Collectively, these results demonstrate that *Gsn* transcriptional upregulation in MASH is dependent on *Atf3* activation, resulting in its pathological overexpression in hepatic tissues, where it exerts a protective role in liver pathophysiology (**Figure** **10**).

## 3 Discussion

Our study investigates the pivotal role of GSN in the development of MASH. Results demonstrated a significant upregulation of GSN expression in both MASH patients and murine models, suggesting that GSN may play a critical role in MASH progression. Further studies revealed that *Gsn* deficiency exacerbates hepatic steatosis and inflammatory fibrosis, whereas AAV‐mediated *Gsn* supplementation effectively ameliorates these pathological changes. This protective effect was also validated in cellular models, indicating the broad applicability of GSN in liver disease.

As an actin‐binding protein, GSN exerts multiple functions by severing and capping actin filaments, thereby modulating cytoskeletal remodeling and cell migration.^[^ ^39^ ^]^ In the context of MASH, our findings revealed that GSN confers hepatoprotective effects by mitigating hepatic steatosis and fibrosis. Elevated GSN expression in MASH patients and murine models may represent a compensatory response to liver injury. The protective role of GSN was underscored by the exacerbation of hepatic steatosis and inflammation in *Gsn*‐deficient mice, while AAV‐mediated Gsn overexpression ameliorated these pathological alterations. These findings suggest that GSN is crucial for maintaining hepatic homeostasis under MASH‐related stress.

Interestingly, our study showed that GSN deficiency led to increased F‐actin accumulation and enhanced activation of YAP in hepatic tissues and liver cell lines. As an actin‐binding protein, GSN primarily functions to regulate the dynamic remodeling of the cytoskeleton by severing and reorganizing actin filaments.^[^ ^21^ ^]^ The balance between filamentous actin (F‐actin) and globular actin (G‐actin) is essential for maintaining cytoskeletal integrity and function.^[^ ^40^ ^]^ Through its actin‐severing activity, GSN promotes the depolymerization of F‐actin into G‐actin, preserving cytoskeletal dynamics.^[^ ^41^, ^42^ ^]^ In our study, GSN deficiency caused excessive intracellular F‐actin accumulation, which disrupted normal cytoskeletal function and was associated with dysregulation of several cellular signaling pathways. Specifically, GSN deficiency significantly enhanced YAP activity in hepatocytes. Under normal conditions, reduced F‐actin polymerization inhibits YAP activity. However, the excessive F‐actin accumulation caused by GSN deficiency diminished this inhibition, leading to YAP hyperactivation, which has important pathological implications in the liver. Aberrant YAP activity is linked to various liver diseases, including fibrosis and hepatocellular carcinoma. Sustained YAP activation promotes abnormal hepatocyte proliferation and resistance to apoptosis, contributing to pathological changes in liver tissue.^[^ ^43^, ^44^ ^]^ Furthermore, the interaction between GSN and F‐actin not only influences YAP activity but may also impact other signaling pathways. Dynamic changes in F‐actin are closely associated with cellular mechanosensing, signal transduction, and gene expression. By regulating F‐actin polymerization and depolymerization, GSN influences various cellular functions and signaling pathways, emphasizing its critical role in cytoskeletal regulation and cellular signaling.

Previous reports suggest that YAP activation predominantly affects liver fibrosis and inflammation but has limited effects on hepatic steatosis. Our findings further support this perspective.^[^ ^45^, ^46^ ^]^ Specifically, in MCD‐fed mice treated with verteporfin to inhibit YAP activity, the results were consistent with expectations. YAP inhibition significantly reduced liver fibrosis and inflammatory responses but had minimal impact on hepatic lipid droplet accumulation. This suggests that although GSN alleviates fibrosis and inflammation partly by suppressing YAP activity, its effects on steatosis may be mediated through independent signaling pathways.

Prior studies have reported that in HepG2 cells, GSN inhibits P53 nuclear translocation by binding to P53, thereby suppressing its activity.^[^ ^30^ ^]^ Recent researches indicate that P53 plays a key role not only in tumorigenesis but also in metabolic regulation and liver pathology, particularly in MASH. P53 activation is closely associated with increased hepatic steatosis and fibrosis.^[^ ^19^, ^47^ ^]^ In MASH, P53 activation promotes adipocyte differentiation and hepatic steatosis.^[^ ^47^ ^]^ Thus, modulating P53 activity is critical for mitigating MASH‐related pathological changes. We hypothesized that GSN might influence hepatic steatosis in MASH by regulating P53 expression. Subsequent experiments revealed a co‐expression relationship between GSN and MDM2, an important E3 ubiquitin ligase that regulates cell cycle and apoptosis by promoting P53 ubiquitination and subsequent degradation. Our findings demonstrated that GSN enhances the binding between P53 and MDM2, facilitating P53 ubiquitination and proteasomal degradation, thereby reducing P53 levels and alleviating its deleterious effects on the liver. Notably, changes in GSN expression did not affect MDM2 expression levels in vivo or in vitro. This indicates that GSN plays a critical role not only in maintaining cytoskeletal integrity but also in regulating P53 degradation through its interaction with MDM2. Further experiments revealed that GSN deficiency significantly increased P53 protein levels, exacerbating hepatic steatosis. Conversely, GSN overexpression markedly reduced P53 levels and ameliorated MASH‐associated pathological changes. This underscores the protective role of GSN in MASH. In addition, the co‐expression and co‐localization of GSN and MDM2 further support this regulatory mechanism. Our findings suggest that GSN enhances MDM2 function, promoting P53 ubiquitination and degradation, thereby exerting protective effects in MASH. Notably, the interaction among GSN, MDM2, and P53 forms a critical regulatory axis with pathological significance in MASH and potentially other metabolic and liver diseases. For example, P53 activation and dysregulation are also closely associated with disease progression in cirrhosis and hepatocellular carcinoma. Thus, modulating the interaction between GSN, MDM2, and P53 could provide novel therapeutic targets and strategies for these diseases.

In this study, we found that GSN expression was aberrantly increased in the livers of three different MASH diet (MCD, CDAHFD, HFD)‐fed mouse models, MASH patients, and PA‐stimulated AML12 hepatocytes. Subsequent investigations further confirmed that the pathological elevation of GSN in hepatocytes plays a significant regulatory role in the development and progression of MASH. However, whether GSN expression is increased in response to specific transcription factors during the pathological process of MASH remains to be explored. A previous study reporting that ATF3 regulates GSN expression as an upstream factor in bladder cancer.^[^ ^34^ ^]^ Further research showed that following PA stimulation in AML12 cells, ATF3 expression was upregulated, and similar increases in ATF3 expression were observed in liver tissues of MASH patients, as well as in the livers of mice fed with three different high‐fat diets. Notably, we identified potential binding sites for ATF3 in the *Gsn* promoter, suggesting that ATF3 and the *Gsn* promoter mutually regulate each other at the transcriptional level in response to MASH progression. Previous studies have also shown that ATF3 plays a crucial role in regulating lipid metabolism and hepatic steatosis, consistent with our findings.^[^ ^36^, ^37^, ^48^ ^]^ These findings provide valuable insight into the source of GSN induction in hepatocytes during MASH and hold significant implications.

Our study provides important insights into the role of GSN in MASH, but certain limitations must be acknowledged. First, the mechanisms underlying the upregulation of GSN expression during MASH progression remain incompletely understood. Although our in vitro and in vivo experiments confirmed that ATF3 enhances GSN transcriptional activity in MASH, the specific regulatory mechanisms between the two genes require further investigation. Experimental data suggest that GSN primarily acts as a protective factor, emphasizing its critical role in safeguarding liver health under pathological conditions. Elucidating these mechanisms is essential for comprehensively understanding MASH pathogenesis, with significant implications for advancing research and developing targeted therapies. Second, although we observed the effects of GSN on macrophage activation and infiltration, these aspects were not explored in depth due to experimental limitations. Since macrophages play a crucial role in hepatic inflammation and fibrosis, further research is needed to clarify how GSN influences macrophage activity. In addition, the GSN‐MDM2‐P53 ternary complex suggests a complex regulatory mechanism that requires further experimental validation. Detailed structural studies will be critical for identifying specific binding sites and interactions among these proteins. Finally, the absence of liver‐specific GSN knockout mice in our study represents a limitation. Employing such models in future research will provide more precise insights into the role of GSN in liver biology and MASH pathology.

In summary, our findings not only deepen the understanding of the protective mechanisms of GSN in MASH but also provide a valuable reference for the development of GSN‐based diagnostic and therapeutic strategies. These discoveries hold significant implications for advancing MASH research and treatment.

## 4 Experimental Section

### Human Liver Samples

Human liver tissue samples used in this study were collected from patients undergoing liver biopsy, liver surgery, or liver transplantation for clinical purposes. Pathological diagnoses were performed by experienced pathologists to ensure the reliability and accuracy of the study. To minimize potential confounding factors, samples were excluded from patients with viral hepatitis (e.g., HBV or HCV infection), those with a history of excessive alcohol consumption (defined as >140 g week^−1^ for men or >70 g week^−1^ for women), and individuals with a known history of drug‐induced liver injury (DILI). Prior to the commencement of the study, all participants, including patients and healthy volunteers, provided written informed consent. The study adhered strictly to the ethical principles outlined in the 1975 Declaration of Helsinki. Ethical approval for the study was granted by the Ethics Committee of Sir Run Run Shaw Hospital, Zhejiang University, ensuring that all procedures were conducted in compliance with ethical guidelines and standards.

### Animals

Eight‐week‐old wild‐type mice were provided by Shanghai SLAC Laboratory Animal Co., Ltd. The construction and identification of *Gsn* KO mice were conducted using PCR genotyping and sequencing methods. Two specific primers, *Gsn*‐F (TTGATTCTCCTCTAGCACAGG) and *Gsn*‐R (AAGGCCTGGTGTAGAGAGTACTC), were designed to amplify WT and KO alleles. The PCR conditions were as follows: initial denaturation at 94 °C for 2 min, 35 cycles of 98 °C for 10 s, 60 °C for 30 s, and 68 °C for 45 s, with a final extension at 68 °C for 10 min. The reaction utilized KOD FX polymerase. PCR products were analyzed on a 1.5% agarose gel, revealing the following genotypes: WT produced a single 405 bp band, KO produced a 407 bp band, and heterozygotes showed both 405 and 407 bp bands. Genotypes were further confirmed by sequencing. WT sequences displayed a single peak matching the reference sequence, KO sequences contained a 2 bp insertion with a single peak, and heterozygotes showed double peaks representing both WT and mutant sequences, the specific sequence results are provided in Table S1 (Supporting Information).

All mice were kept in pathogen‐free environments with unrestricted access to food and water. They were fed a standard chow diet (STD, D12450B), a high‐fat diet (HFD, D12492), a methionine‐choline deficient diet (MCD, TP3005G), or a choline‐deficient, L‐amino acid‐defined, high‐fat diet (CDAHFD, A06071302). Environmental conditions were maintained at 40%–50% humidity and 20 ± 3 °C, under a 12‐h light/dark regimen. To inhibit YAP expression, intraperitoneal injections of verteporfin (VP; MCE, HY‐B0146 ,100 mg kg^−1^) or corn oil (MCE, HY‐Y1888 10 mL kg^−1^) were administered every other day to *Gsn* knockout mice for a total duration of 14 d.^[^ ^49^ ^]^ To inhibit P53 expression, mice received intraperitoneal injections of the P53 inhibitor pifithrin‐α (PFT; MCE, HY‐15484,2.2 mg kg^−1^, in physiological saline) or control vehicle dimethyl sulfoxide (DMSO; MCE, HY‐Y0320, in physiological saline) three times per week for 4 weeks.^[^ ^50^ ^]^ After a predetermined feeding period, liver samples from each group were collected and either stored at −80 °C or fixed in 10% buffered formalin. All animal procedures adhered to the Guidelines for Care and Use of Laboratory Animals of Zhejiang University. The study received approval from the Experimental Animal Welfare Ethics Review Committee at Zhejiang University (AP Code: NO. ZJU20240923).

### Administration of Adeno‐Associated Viral Vectors

Mouse *Gsn* gene‐encoding AAV8 vectors were acquired from OBiO Biotechnology (Shanghai, China). Following a period of adaptation and acclimatization, male C57BL/6J mice were split into two groups at random. One bolus injection of either AAV8 encoding pADV‐EF1‐mScarlet‐CMV‐MCS‐3xFLAG or AAV8 encoding *Gsn* was given to each animal via the tail vein (1 × 10¹^2^ vg mL^−1^, 100 µL per mouse). GSN expression in the mice's liver tissues was measured four weeks after injection using immunoblot and Q‐PCR. Every step of the process followed strict experimental guidelines and was carried out to guarantee the precision and dependability of the outcomes.

### Histopathological Analysis

Liver samples were initially fixed with 4% formaldehyde overnight to ensure optimal tissue preservation and structural stability. After fixation, the samples were processed using different methods depending on the intended analysis. Paraffin was used to embed some of the samples, making it easier to prepare thin, uniform tissue sections for close inspection. After being embedded in paraffin, these samples were sectioned into 3–5 µm slices and stained with H&E, a standard method in histology. Concurrently, in order to prepare other samples as fixed sections, they were imbedded in optimal cutting temperature (OCT) compound. These OCT‐embedded liver samples were sectioned at 3–8 µm and subjected to Oil Red O staining (Solarbio, Cat#G1261). Oil Red O staining is particularly useful for visualizing lipid accumulation within the liver tissue, a critical indicator of steatosis. This staining method involves incubating the fixed sections in a 60% Oil Red O solution for 10–15 min, followed by counterstaining with hematoxylin to provide contrast. The red coloration from Oil Red O highlights lipid droplets, allowing for the purpose to determine the extent of steatosis in the liver. To evaluate liver fibrosis, Sirius Red staining (PSR; Solarbio, Cat#G1472) was used. Sirius Red specifically binds to collagen fibers, staining them red and thereby facilitating the visualization of fibrotic areas. This method involves incubating the liver sections in Sirius Red solution for 1–2 h, followed by washing and dehydration steps.^[^ ^51^ ^]^ After completing all staining procedures, histological images of the tissue sections were acquired using a high‐quality light microscope.

### Immunohistochemistry

Liver tissues were fixed in 4% paraformaldehyde (PFA) and embedded in paraffin. After dewaxing, sections were heated at 95 °C for 20 min in 0.01 m sodium citrate buffer for antigen retrieval. Endogenous peroxidase activity was blocked with 3% hydrogen peroxide for 10 min. Nonspecific binding was prevented using 5% goat serum for 30 min. Sections were incubated overnight at 4 °C with primary antibodies, then washed with PBST and treated with secondary antibodies for 2 h at room temperature. Finally, sections were washed again and examined under a light microscope at 20× magnification.

### Immunofluorescence Staining

Primary antibodies were incubated at 4 °C for a whole night to perform immunofluorescence staining on liver tissue slices embedded in paraffin. Sections were photographed using a fluorescent microscope (DX51, Olympus) after primary antibody incubation and treated with fluorophore‐conjugated secondary antibodies to visualize antigen–antibody complexes.

To do cellular immunofluorescence, primary antibodies that target particular proteins were incubated overnight on cover slips. After the primary antibody was incubated, the cells were counter‐stained with DAPI and stained with secondary antibodies conjugated to fluorophores.

### Analysis of Publicly Accessible Data from the GEO Repository

*Gsn* transcript levels in human liver samples from normal, healthy obese, steatosis, and NASH categories were analyzed. Four microarray datasets (GSE48452, GSE61620, GSE33814, GSE63067) from the GEO database, encompassing 244 samples were used. *Gsn* mRNA expression was shown to be significantly higher in NASH liver tissues than in normal tissues, and there was a positive connection found in dataset GSE193006 between *Gsn* mRNA levels and the NAFLD activity score.

### RNA Sequencing (RNA‐Seq)

Six‐week‐old WT and *Gsn* KO mice were simultaneously fed with an MCD diet for 10 weeks. After confirming the development of MASH pathological phenotypes in both groups, liver tissues were collected from three mice in each group. Total RNA was extracted from the liver tissues using TRIzol reagent and subsequently purified to ensure high‐quality RNA for downstream analyses. High‐throughput sequencing was performed on the Illumina Hiseq X‐ten platform to obtain high‐quality sequencing data. All experimental procedures, including extended protocol details and data analysis, were conducted and supported by Beijing Tsingke Biotech Co., Ltd., ensuring the accuracy and reliability of the results.

### KEGG Pathway Enrichment Analysis

The KEGG pathway enrichment analysis was performed using the Database for Annotation, Visualization and Integrated Discovery (DAVID), an online tool designed for functional annotation and data interpretation. Pathways with a *p*‐value of less than 0.05 were defined as significantly enriched, indicating their potential biological relevance. In the analysis of RNA sequencing data, the input for KEGG pathway enrichment consisted of downregulated differentially expressed genes (DEGs), which were identified as key candidates for further exploration of associated signaling pathways.

### Gene Set Enrichment Analysis

Gene set enrichment analysis (GSEA) was conducted utilizing the Java GSEA platform. For each KEGG biological pathway, the associated genes were categorized into a defined gene set. A ranked list of genes was then generated based on their expression levels, and “gene set” permutations were performed to assess statistical significance. Gene sets with a p‐value of less than 0.05 and a false discovery rate (FDR) below 0.25 were deemed statistically significant. These significant gene sets suggest potential involvement in critical biological processes, offering meaningful insights into the molecular pathways underlying the observed data. This method effectively combines gene expression profiles with pathway analysis, facilitating the identification of biologically relevant functional gene sets.

### Gene Set Variation Analysis

Gene Set Variation Analysis (GSVA) was conducted utilizing the GSVA R package to investigate functional variations in gene expression data. Single‐sample GSEA scores were computed for each KEGG pathway containing a minimum of ten genes, leveraging the associated gene expression matrix to assess pathway activity. The significance of these enrichment scores was rigorously evaluated using the limma R package, with pathways achieving a *p*‐value of <0.05 being identified as significantly differentially expressed gene sets. To provide a clear visualization of the variations in pathway activity under different conditions, heatmaps displaying the GSVA scores for these differentially expressed gene sets were generated using the Pheatmap R package.

### Biochemical Serum Analysis

Blood samples from mice were collected and placed in non‐anticoagulant tubes. The samples were centrifuged at 2000–3000 rpm/4 °C for 10–15 min to extract serum from cellular constituents after the blood had had time to coagulate. After the serum was thoroughly aspirated, it was either examined right away or kept in storage at −80 °C. The Cobas C111 biochemical analyzer (Roche) was used to measure the ALT and AST levels in accordance with the manufacturer's instructions.

### ELISA

The concentrations of inflammatory cytokines in mouse serum and cell culture supernatants such as TNF‐α (Proteintech, Cat No. KE10002), IL‐6 (Proteintech, Cat No. KE10007), and IL‐1β (Proteintech, Cat No. KE10003) were measured in accordance with the manufacturer's instructions using ELISA kits. All assays were performed following the provided protocols to ensure accuracy and reliability of the results.

### Cell Lines and Treatment

The Chinese Academy of Sciences Cell Bank (Shanghai, China) offered the HEK293T, and AML12 (mouse normal liver cell line) cells. To ensure there was no mycoplasma contamination, these cells were grown in DMEM supplemented with 10% fetal bovine serum (FBS) and 1% penicillin‐streptomycin (Gibco). The cells were kept at 37 °C in a humidified environment with 5% CO2 (Thermo Fisher Scientific, Waltham, MA). For transfection experiments, Lipofectamine 3000 was used as the transfection reagent to deliver various plasmids or small interfering RNAs (siRNAs) into the cells. Negative control siRNA or empty vector was used as controls. Post‐transfection, cells were cultured under the aforementioned conditions to ensure smooth conduct of experiments and reliability of results. These experiments enable in‐depth investigation into the functions and mechanisms of different genes and proteins in these cell lines. To simulate hepatic steatosis in vitro, AML12 cells were treated with specified concentrations of PA (Sigma‐P0500) for 24 h and subjected to 0.3 × 10^−3^ m PA. As a control, BSA (Sigma Aldrich) devoid of fatty acids was utilized. For the cytochalasin experiment, first, select well‐conditioned cells and culture them to the desired density. After reaching the appropriate density, induce the cells with PA. After 12 h of PA treatment, add 10 × 10^−6^ m CB (MCE, HY‐16928; diluted in DMSO), with DMSO used as a control. Continue to culture the cells under these conditions for an additional 12 h to ensure adequate treatment response. Following this incubation period, proceed with subsequent experimental analyses to evaluate the effects of cytochalasin on the cells. For the in vitro P53 inhibition experiments, cells were cultured with 20 × 10^−6^ m of the P53 inhibitor Pifithrin‐α (PFT; MCE, HY‐15484) for 2 h, with PBS used as the control. Following stimulation with either BSA or PA, the cells were collected by centrifugation for subsequent experimental analysis. The experimental design allowed for the evaluation of P53 inhibition effects in response to different stimuli, and the collected samples were subjected to further assays to assess changes in P53 activity and related cellular responses.

Si‐*Gsn*‐1: 5′ GGCUUCAACUGGCUCAAAU 3′; Si‐*Gsn*‐1: 3′ UUUGAGCCAGUUGAAGCCG5;

Si‐*Gsn*‐2: 5′ GGAUCAAGGCUACUGGAUU 3′; Si‐*Gsn*‐2: 3′ UCCAGUAGCCUUGAUCCTT5;

Si‐*Gsn*‐3: 5′ GGAAGUGCUGCUGGAUGAUU 3′; Si‐*Gsn*‐3: 3′ UCAUCCAGCAGCACUUCCG;

Si‐*Mdm2*‐1: **5′** GCCAGUAUAUUAUGACUAA **3′;** Si‐*Mdm2*‐1: **3′** UUAGUCAUAAUAUACUGGC **5′;**

Si‐*Mdm2*‐2: **5′** CGCCACAAAUCUGAUAGUA **3′;** Si‐*Mdm2*‐2: **3′** UACUAUCAGAUUUGUGGCG **5′;**

Si‐*Mdm2*‐3: **5′** GCCUGCUUUACAUGUGCAA **3′;** Si‐*Mdm2*‐3: **3′** UUGCACAUGUAAAGCAGGC **5′**

### Isolation and Culture of Primary Hepatocytes from Mice

Mice were anesthetized with pentobarbital, and the liver was exposed by incising the diaphragm. The inferior vena cava was cannulated, and the liver was perfused with Liver Perfusion Solution I (IMP‐MK001B, IMMOCELL) at 5 mL min^−1^ until it turned waxy yellow, followed by Liver Perfusion Solution II (IMP‐MK001C) at 3 mL min^−1^ with rhythmic portal vein compression for 5–15 min. The liver was excised, and hepatocytes were released by gentle agitation in DMEM with 10% FBS. The cell suspension was filtered through a 70 µm mesh (BS‐70‐XBS, Biosharp), centrifuged at 50 *g* for 5 min, and resuspended in DMEM with 10% FBS. Hepatocytes were plated on Type I rat tail collagen (354236, Corning)‐coated dishes after washing with PBS.

### Cellular Oil Red O Staining

PA was used to treat AML12 cells to cause lipid buildup. To maintain cellular architecture, cells were fixed in 4% paraformaldehyde for 15 min after treatment. A typical technique for identifying neutral lipids in cells, 60% Oil Red O solution (Solarbio, G1262) was used to visualize lipid accumulation. To guarantee sufficient lipid staining, the cells were incubated in the staining solution for 15–20 min. After that, the stained cells were seen using an Olympus light microscope in Tokyo, Japan, to determine the degree of lipid buildup.

### Construction of the Lentivirus Vector

HEK293T cells were first grown in DMEM media with 10% fetal bovine serum added until the cell density was suitable. Next, either Lipofectamine 3000 or polyethyleneimine (PEI) was used to cotransfect the cells. The virus‐containing supernatant was collected 48 hours after infection and filtered using a 0.45 µm filter (Millipore, Burlington, USA) to get rid of extraneous contaminants and cell debris.

Before infecting AML12 cells, they were raised until they reached the logarithmic growth phase in DMEM media supplemented with 10% fetal bovine serum. The filtered viral supernatant was then directly added to the culture dish, exposing AML12 cells to the viral particles. To enhance infection efficiency, polybrene or other enhancers were typically added during the infection process. After 24 h of infection, new culture medium was added to the medium, and the cells were continued to be cultured. Within the next 72 h, the cells were selected using puromycin‐containing medium (30 µg mL^−1^) to ensure that only cells with the integrated target gene survived. After selection, these cells were expanded and seeded for subsequent experiments.

*Gsn*‐sgRNA‐1: **5′** GACTTCATGCTGGGCTTACC **3′;**

*Gsn*‐sgRNA‐2: **5′** CTGGTAGCAGTCTGTTGACC **3′;**

*Atf3*‐shRNA‐1: **5′** CCGG‐CTTCATCGGCCCACGTGTATT‐CTCGAG‐AATACACGTGGGCCGATGAAG‐TTTTTT **3′;**

*Atf3*‐shRNA‐2:**5′** CCGG‐ACGAGAAGCAGCATTTGATAT‐CTCGAG‐ATATCAAATGCTTCTCGT‐TTTTTT **3′;**

*Atf3*‐shRNA‐3: **5′** CCGG‐AGATGAGAGAAACCTCTTTAT‐CTCGAG‐ATAAAGAGGTTTCTCTCATCT‐TTTTTT **3′;**

### Plasmids

The construction methods for FLAG‐*Gsn*, MYC‐*Mdm2*, and HA‐*Ubiquitin* plasmids followed the procedures described in previous literature.^[^ ^52^ ^]^ Specifically, the target gene sequences were cloned into the corresponding plasmid vectors, and high‐purity recombinant plasmids were obtained through bacterial transformation and screening. Subsequently, high‐quality plasmid DNA was extracted using both small‐scale and large‐scale plasmid extraction methods for cell transfection. The interference efficiency was assessed by Western blot, which detects changes in protein levels on the target gene.

### Construction of *Gsn* Mutant Plasmid

The construction of *Gsn* mutant plasmids was achieved using site‐directed mutagenesis by aligning the sequences and designing specific mutation strategies for the target amino acid residues. Specifically, AAA at position 100 in the first 740 bp was mutated to DDD, and RLK at position 210 was mutated to AAA. The designed mutation sequences were GACGACGAC (for DDD) and GCGGCCGCC (for AAA). Using a plasmid containing the wild‐type *Gsn* gene as a template, site‐specific primers were employed to introduce mutations via PCR amplification. The amplified products were ligated, transformed into *E. coli*, and positive clones were screened. The mutated regions were confirmed by sequencing, which showed that the nucleotide substitutions at the target sites were correct and consistent with the designed mutation sequences. The successfully constructed plasmids are suitable for subsequent studies on the functions of *Gsn* mutants and their roles in cellular processes, the specific sequence results are provided in Table S2 (Supporting Information).

### Total Triglycerides Extraction and Quantification

We extracted total triglycerides (TG) from mouse liver tissues or AML12 cells. Specifically, mouse liver tissues or AML12 cells were extracted and then centrifuged at 3000 rpm for ≈15 min at 4 °C. After centrifugation, the lower organic phase was collected and washed with 0.9% sodium chloride solution to remove any water‐soluble impurities. The washed organic phase was subjected to rotary evaporation to remove the solvent, leaving behind the lipid components. The remaining lipids were then dissolved in an appropriate amount of isopropanol to ensure complete dissolution for subsequent assays. After extracting and dissolving the lipids, we utilized commercial kits, following the manufacturer's instructions to measure the TG (PPLYGEN, E1025‐105) contents in the cells or tissues.

### Hepatic Hydroxyproline Content

The collagen content in liver tissue was assessed by determining the hydroxyproline levels. This was performed using the Hydroxyproline Detection Kit (Cat# BC0250, Solarbio) in accordance with the manufacturer's instructions.

### Isolation and Detection of G‐Actin and F‐Actin

Cytoskeleton Inc.’s G‐Actin/F‐Actin In Vivo Assay Kit (#BK037) was used to measure the levels of F‐actin and G‐actin. In order to separate soluble G‐actin from insoluble F‐actin, liver tissue or cells were homogenized in F‐actin stabilizing buffer and centrifuged. To change F‐actin into G‐actin, the pellet was resuspended in F‐actin destabilizing buffer after the supernatant, which contained G‐actin, was transferred. After that, proteins were examined using anti‐actin antibodies in a Western blot. G‐actin and F‐actin can be distinguished and quantified using this technique, providing information about cytoskeletal movements.

### F‐Actin Staining

After culturing cells to 70%–80% confluence, they were fixed with 4% PFA in PBS for 15 min to preserve cellular structural integrity. Subsequently, the cells were permeabilized with 0.1% Triton X‐100 for 10 min to facilitate dye penetration into the cellular interior. Next, nonspecific binding was blocked using 5% BSA in PBS for 1 h. Following these preparatory steps, cells were incubated overnight with primary antibodies as described earlier. The next day, secondary antibodies conjugated to fluorophores of different wavelengths were applied to label the primary antibodies. Subsequently, the cells were stained with F‐actin dye (AAT Bioquest, catalog #22660) at a 1:100 dilution for 1 h to visualize the cytoskeletal F‐actin structure. After staining, the samples were gently rinsed several times with PBST buffer (PBS containing 0.1% Tween‐20) to remove unbound dye. The nuclei were counterstained with DAPI at a concentration of 1 µg mL^−1^ to facilitate nuclear visualization. Fluorescent images were captured using a fluorescence microscope. Image analysis was performed to assess the distribution of F‐actin and evaluate cellular morphology.

### Quantitative PCR Analysis and Western Blots

Total RNA was extracted using TRIzol reagent (T9424; Sigma‐Aldrich, St. Louis, MO), a widely used method for efficiently isolating RNA from cellular samples. TRIzol reagent uses a phenol‐chloroform mixture to lyse cells and separate RNA from other cellular components. Samples were mixed thoroughly with TRIzol and subjected to centrifugation, resulting in an aqueous phase containing RNA. After assessing the quality and concentration of the extracted RNA, qPCR was performed using a Real‐Time PCR System (LightCycler 480 Instrument II, Roche Diagnostics Inc., Basel, BS, Switzerland). Standard procedures were followed to ensure the reproducibility and accuracy of results. Gene expression levels were normalized to the reference gene β‐actin. Primer sequences were meticulously designed to ensure accuracy and reliability of the experiments.

Protease and phosphatase inhibitors were added to RIPA (beyotime, China) solution before tissues and cells were lysed. After centrifuging the lysates, the BCA assay was used to determine the protein content. After being separated by 10% SDS‐PAGE, equal amounts of protein were transferred to PVDF membranes. Membranes were blocked using 5% skim milk in TBST and then incubated with primary antibodies and HRP‐conjugated secondary antibodies for a whole night at 4 °C. ECL was used to identify protein signals, which the ChemiDoc MP Imaging System then displayed. Accurate results were ensured by using specific antibodies specified in Table S5 (Supporting Information) and according to established guidelines during the experimental procedures.

### Immunoprecipitation Assays

Immunoprecipitation (IP) and co‐immunoprecipitation (co‐IP) assays were performed as follows: 293T cells were co‐transfected with designated plasmids and incubated for 24–48 h. Cells were lysed with IP buffer (20 × 10^−3^ m Tris‐HCl, 150 × 10^−3^ m NaCl, 1 × 10^−3^ m EDTA, 1% NP‐40) containing a protease inhibitor cocktail. The lysate was centrifuged, and the supernatant was incubated with protein G agarose beads and specific antibodies overnight at 4 °C. After washed with NaCl buffer, beads were resuspended in 5× SDS loading buffer and heated. Proteins were dissociated and analyzed by SDS‐PAGE followed by Western blotting. For endogenous IP, cells or liver tissues were treated similarly to the co‐IP procedure, but without the introduction of plasmids.

### Cycloheximide Assay

In the CHX assay, CHX (20 µg mL^−1^), a potent inhibitor of protein synthesis, was added to sg‐Ctrl AML12, sg‐*Gsn* AML12, PLVX AML12, and PF‐*Gsn* AML12 cells. Protein lysates were collected at the indicated time points and analyzed by immunoblotting.

### Ubiquitination Assays

To investigate the ubiquitination of exogenous P53, 293T cells were transfected with the plasmids of interest and subsequently treated with 20 × 10^−6^ m MG132 for 6 h to inhibit the 26S proteasome and block proteasomal degradation. After the treatment, cells were harvested and lysed in a suitable lysis buffer. The protein lysates were subsequently incubated with magnetic beads conjugated to FLAG, MYC, or HA tag antibodies separately at 4 °C for 8 h, allowing for the immunoprecipitation of ubiquitinated P53 complexes. After incubation, the beads were washed thoroughly with lysis buffer to remove nonspecifically bound proteins. The purified proteins were then analyzed by immunoblotting to detect the ubiquitinated P53. For endogenous P53 ubiquitination, cells were treated similarly with MG132 and lysed using IP lysis buffer, which preserves protein integrity while allowing efficient protein extraction. The expression levels of P53 were quantified by immunoblotting, and the P53 protein was subsequently immunoprecipitated using a P53‐specific antibody (Proteintech, USA). The immunoprecipitated complexes were then subjected to Western blotting to analyze ubiquitination.

### ChIP‐qPCR

The ChIP assay was conducted using a commercial advanced kit (CST, catalog #9003) according to the manufacturer's standard protocol. The detailed steps are as follows: First, chromatin in AML12 cells was cross‐linked using 1% formaldehyde to stabilize protein–DNA complexes. Cells were then lysed to isolate nuclei, and the chromatin was released using a nuclear extraction buffer. The chromatin was subsequently fragmented via ultrasonic shearing, yielding DNA fragments ranging from 200 to 1000 bp. The sonication conditions were carefully optimized to ensure consistent and appropriate fragment sizes. Next, the soluble chromatin was incubated with an anti‐*Atf3* antibody or IgG (negative control) to perform immunoprecipitation, selectively enriching DNA fragments bound to *Atf3*. RNase A and proteinase K were then added to digest RNA and proteins in the de‐crosslinking step. Following this, the DNA was extracted using phenol/chloroform to ensure high purity. The purified DNA was precipitated with ethanol and subsequently analyzed using agarose gel electrophoresis to confirm its integrity and quality. Finally, PCR amplification was carried out using specific primers to verify the binding of *Atf3* to the promoter region of the target gene. The primer pair sequences used for amplifying the PCR product containing the *Atf3* site are listed below:

Forward primer: 5′‐AGTGGGAGTCCCTGAGGTCT‐3′

Reverse primer: 5′‐ATAGCAAGCAACCAGCACAG‐3′

### Luciferase Reporter Assay

After transfecting sh*Atf3* plasmids into AML12 cells, each well was transfected with the pGL3 vector or pGL3‐Gsn promoter reporter plasmid, along with the Renilla luciferase plasmid pRL‐TK. Forty‐eight hours post‐transfection, cells were treated with PA for 24 additional hours, followed by lysis using passive lysis buffer. Luciferase activity was then measured according to the manufacturer's protocol using the Dualucif Firefly & Renilla Assay Kit System (F6075S, UElandy, Suzhou, China).

### Statistical Analysis

Data are presented as mean ± SEM and analyzed using GraphPad Prism 9.0. Statistical significance between two groups was assessed with a two‐tailed Student's *t*‐test, while multiple groups were compared using one‐way or two‐way ANOVA. Tukey's or Dunnett's post‐tests were used for further analysis. Two‐way ANOVA was used for experiments with two independent variables. Statistical methods, *P*‐values, and sample sizes are detailed in the figure legends. No data were excluded, and a *P*‐value < 0.05 was considered significant.

## Conflict of Interest

The authors declare no conflict of interest.

## Author Contributions

Y.L., T.J., Z.Y., and J.Y. contributed equally to this work. Y.L.: conceptualization, formal analysis, methodology, data curation, investigation, visualization, and writing original draft. T.J.: data curation, formal analysis, visualization, and writing original draft. J.Y.: data curation, formal analysis. Z.Y.: data curation and formal analysis. J.C. and Z.J.: data curation and formal analysis. Y.Z.: visualization. X.C.: conceptualization, resources, methodology, and validation. Y.W.: funding acquisition, supervision, and writing review & editing.

## Supporting information



Supporting Information

## Data Availability

The data that support the findings of this study are available on request from the corresponding author. The data are not publicly available due to privacy or ethical restrictions.
