# Supplementary material for: Gelsolin's Protective Role in MASH through F‐Actin Regulation and P53 Degradation
Source: Adv Sci (Weinh). 2025 May 20;12(23):2416489. doi: 10.1002/advs.202416489 (PMC12199436; doi:10.1002/advs.202416489)
Supplement: Supplementary file 1 — Supporting Information [file ADVS-12-2416489-s001.docx]

**Gelsolin's Protective Role in MASH through F-actin Regulation and P53 Degradation**

Yiwei Lu^1,2,3,#^, Tong Ji^1,2,3,#^, Zhichao Ye^1,2,3,#^, Jianing Yan^1,2,3,#^, Chao Wang^1,2,3,^,Jiachen Chen^1,2,3，^Ziyang Jin^1,2,3^, Yongji Zhu^1,2,3^, Xiujun Cai^1,2,3,^*,Yifan Wang^1,2,3,^*

^1^Department of General Surgery, Sir Run Run Shaw Hospital Affiliated to School of Medicine, Zhejiang University, Hangzhou 310016, China.

^2^National Engineering Research Center of Innovation and Application of Minimally Invasive Instruments, Hangzhou 310016, China.

^3^Zhejiang Provincial Key Laboratory of Laparoscopic Technology, Sir Run Run Shaw Hospital Affiliated to School of Medicine, Zhejiang University, Hangzhou 310016, China.

**^#^These authors contribute equally.**

***Corresponding authors:** srrsh_cxj@zju.edu.cn and anwyf@zju.edu.cn.

**Supplementary figures**

**Figure S1**

**
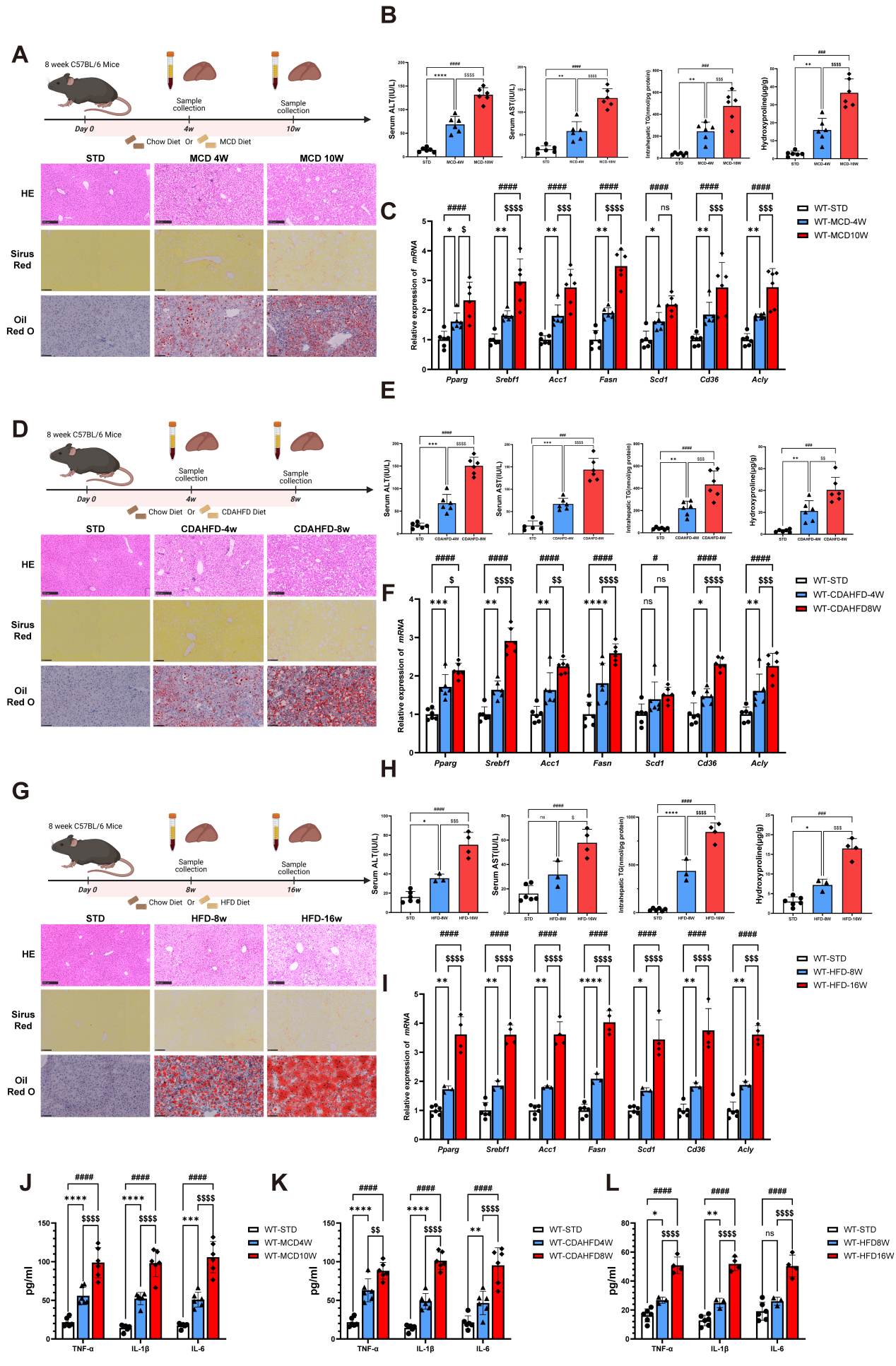
**

**Figure S1. Construction and validation of three diet-induced MASH mouse models.**

A. Representative images from hematoxylin and eosin (H&E) staining (50x, Scale bars: 250 µm), sirius red staining (100x, Scale bars: 250 µm) and Oil Red O staining (100x, Scale bars: 250 µm) were obtained for each experimental group from MCD-fed mice (n=6/group);B. Quantification of serum ALT, AST, hepatic triglyceride and Hydroxyproline (HYP) content in the liver tissues in each experimental group from MCD-fed mice (n=6/group);C. Quantitative PCR analyses of the expression of lipid metabolism molecules in liver tissues from MCD-fed mice (n=6/group);D. Representative images from H&E staining (50x, Scale bars: 250 µm), sirius red staining (50x, Scale bars: 250 µm) and Oil Red O staining (50x, Scale bars: 250 µm) were obtained for each experimental group from CDAHFD-fed mice (n=6/group);E. Quantification of serum ALT, AST, hepatic triglyceride and Hydroxyproline (HYP) content in the liver tissues in each experimental group from CDAHFD-fed mice (n=6/group);F. Quantitative PCR analyses of the expression of lipid metabolism molecules in liver tissues from CDAHFD-fed mice (n=6/group);G. Representative images from H&E staining (50x, Scale bars: 250 µm), sirius red staining (100x, Scale bars: 250 µm) and Oil Red O staining (100x, Scale bars: 250 µm) were obtained for each experimental group from HFD-fed mice (n=3-6/group);

H. Quantification of serum ALT, AST, hepatic triglyceride and Hydroxyproline (HYP) content in the liver tissues in each experimental group from HFD-fed mice (n=3-6/group);

I. Quantitative PCR analyses of the expression of lipid metabolism molecules in liver tissues from HFD-fed mice (n=3-6/group);J-L. ELISA results for inflammatory cytokines (TNF-α，IL-1β，IL-6) in Serum from MCD-fed mice(J), CDAHFD-fed mice(K) and HFD-fed mice(L), (n=3-6/group);Data were expressed as the means ± standard deviation (SD). Significant differences were determined using one-way ANOVA as appropriate. A 2-tailed p < 0.05 was considered statistically significant. (*p<0.05, **p <0.01, ***p <0.005, ****p <0.001, n.s., no signiﬁcance).

**Figure S2**

**
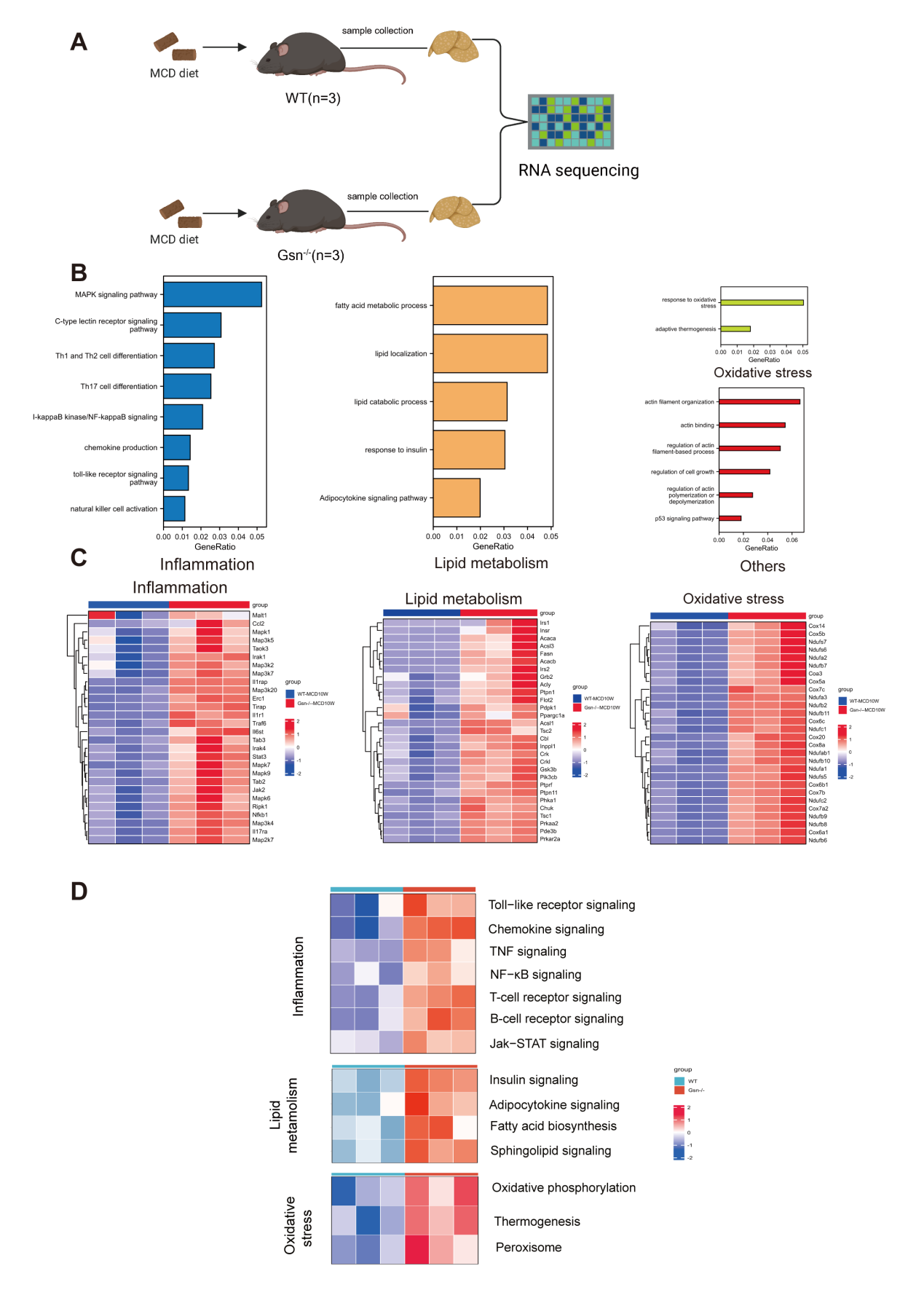
**

**Figure S2**. **RNA Sequencing of Mouse Liver Tissues Reveals Significant Impact on Multiple NASH-Related Pathways Following *Gsn* Knockout**

A. Flowchart for RNA Sequencing of Mouse Liver Tissues;B. KEGG analysis showing the significantly altered pathways related to inflammation, lipid metabolism and oxidative phosphorylation based on the RNA-Seq data sets;C. GSEA analysis showing the significantly altered genes related to inflammation, lipid metabolism and oxidative phosphorylation from RNA-seq datasets;D. GSVA analysis showing the significantly altered pathways related to inflammation, lipid metabolism and oxidative phosphorylation based on the RNA-Seq data sets.

**Figure S3**

**
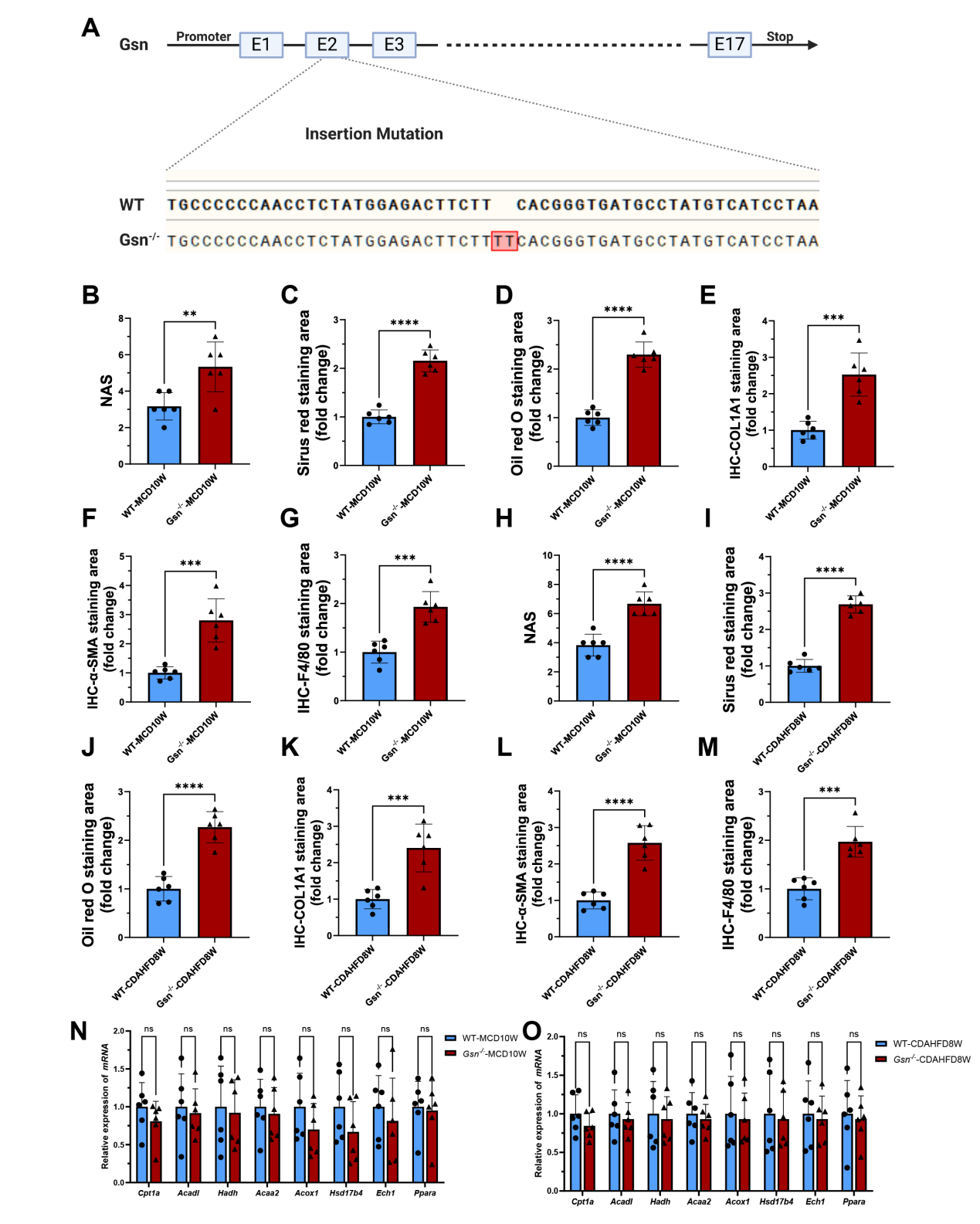
**

**Figure S3. GSN deficiency led to more severe liver inflammation, fibrosis, and steatosis in both diet-induced MASH mouse models.**

A. Schematics and sequencing validation for insertion mutation in *Gsn* gene. Created in BioRender.com;B. Quantification of NAS score of liver tissues in each experimental group from WT/*Gsn*^-/-^ mice fed with MCD (n=6/group);C. Quantification of Sirus red staining area of liver tissues in each experimental group from WT/*Gsn*^-/-^ mice fed with MCD (n=6/group);D. Quantification of Oil red O staining area of liver tissues in each experimental group from WT/*Gsn*^-/-^ mice fed with MCD (n=6/group);E. Quantification of COL1A1 histological staining area of liver tissues in each experimental group from WT/*Gsn*^-/-^ mice fed with MCD (n=6/group);F. Quantification of α-SMA histological staining area of liver tissues in each experimental group from WT/*Gsn*^-/-^ mice fed with MCD (n=6/group);G. Quantification of F4/80 histological staining area of liver tissues in each experimental group from WT/*Gsn*^-/-^ mice fed with MCD (n=6/group);H. Quantification of NAS score of liver tissues in each experimental group from WT/*Gsn*^-/-^ mice fed with CDAHFD (n=6/group);I. Quantification of Sirus red staining area of liver tissues in each experimental group from WT/*Gsn*^-/-^ mice fed with CDAHFD (n=6/group);J. Quantification of Oil red O staining area of liver tissues in each experimental group from WT/*Gsn*^-/-^ mice fed with CDAHFD (n=6/group);K.Quantification of COL1A1 histological staining area of liver tissues in each experimental group from WT/*Gsn*^-/-^ mice fed with CDAHFD (n=6/group);L.Quantification of α-SMA histological staining area of liver tissues in each experimental group from WT/*Gsn*^-/-^ mice fed with CDAHFD (n=6/group);M.Quantification of F4/80 histological staining area of liver tissues in each experimental group from WT/*Gsn*^-/-^ mice fed with CDAHFD (n=6/group);N.Quantitative PCR analyses were performed to assess the expression of fatty acid beta-oxidation-related markers in liver tissues of WT/*Gsn*^-/-^ mice fed with MCD (n=6/group);O. Quantitative PCR analyses were performed to assess the expression of fatty acid beta-oxidation-related markers in liver tissues of WT/*Gsn*^-/-^ mice fed with CDAHFD (n=6/group).Significant differences were determined using Student’s t test. A 2-tailed p < 0.05 was considered statistically significant. (*p<0.05, **p <0.01, ***p <0.005, ****p <0.001, n.s., no signiﬁcance).

**Figure S4**.


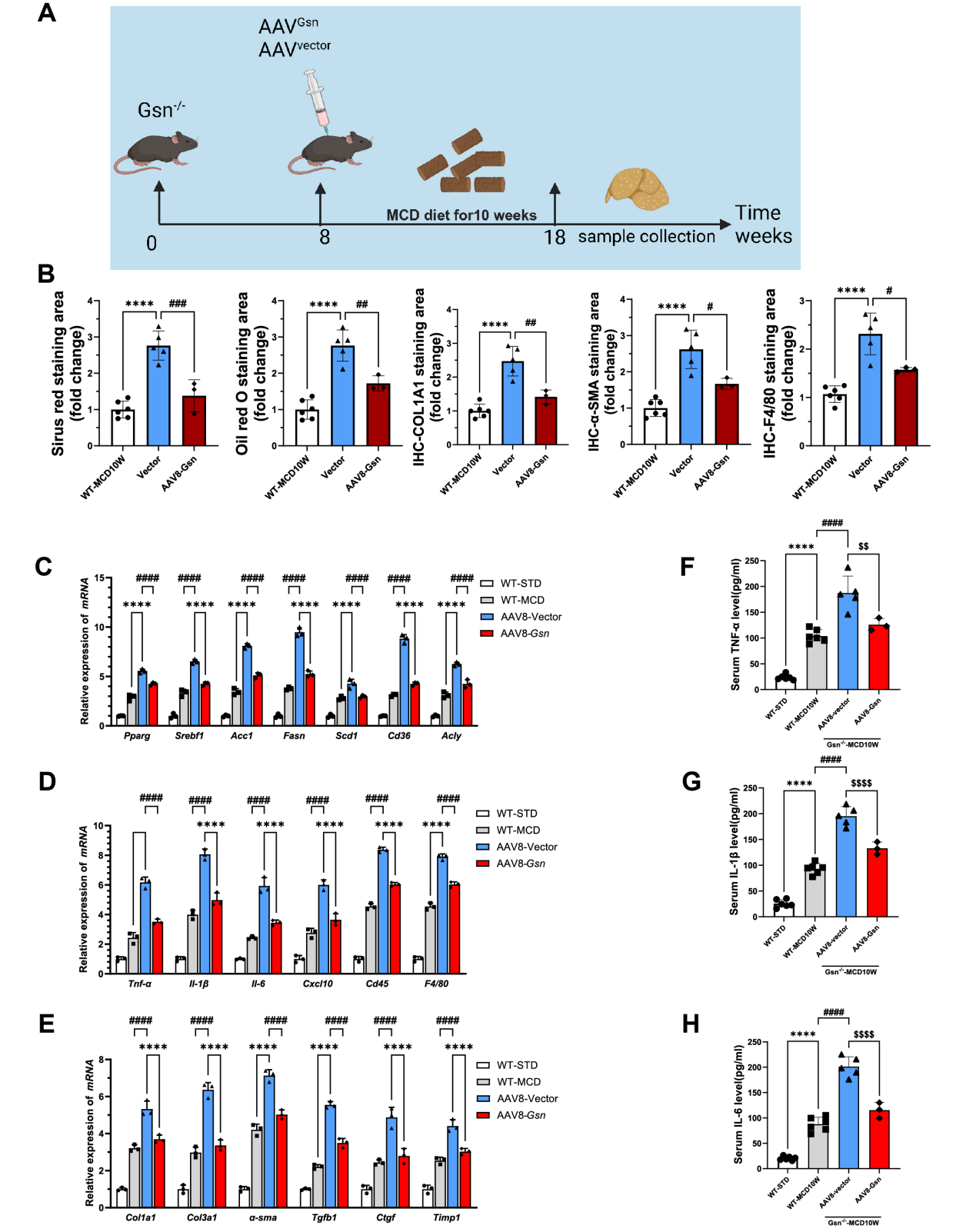


**Figure S4**. **Restoration of *Gsn* Alleviates Hepatic injury in MCD-fed *Gsn*^-/-^ mice.**

A.Experimental Design Workflow Diagram;B.The statistical analysis results of the staining images in Fig. 4A-B.C-E. Quantitative PCR analyses were performed to assess the expression of lipid metabolism molecules (C), inflammatory response markers (D) and fibrosis-related markers (E) in liver tissues for each experimental group (n=3/group); F-H. ELISA results for inflammatory cytokines (TNF-α，IL-1β，IL-6) in serum of the specified groups of mice (n=3-6/group);Data were expressed as the means ± standard deviation (SD). Significant differences were determined using one-way ANOVA as appropriate. A 2-tailed p < 0.05 was considered statistically significant. (*p<0.05, **p <0.01, ***p <0.005, ****p <0.001, n.s., no signiﬁcance).

**Figure S5**

**
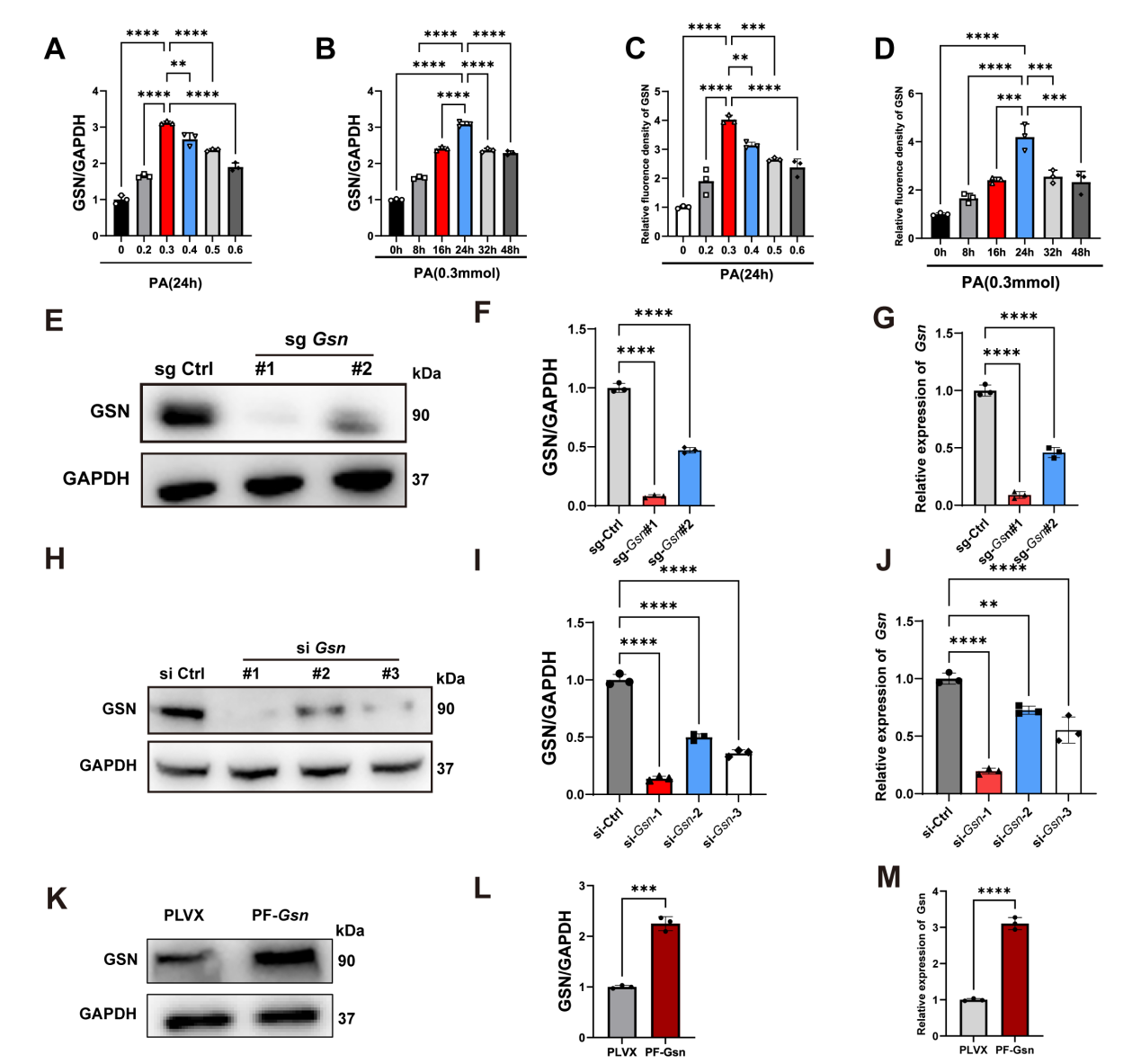
**

**Figure S5. Effects of *Gsn* Knockdown and Overexpression in Different Conditions in AML12 cells.**A-B. Quantitative data of Western blot results from Figure 4A-B;C-D. Quantitative data of immunofluorescence staining results from Figure 4C-D;E-F. Western blot analysis was used to assess the knockout efficiency of two *Gsn*-targeting sgRNAs in AML12 cells;G. Quantitative PCR analyses was used to assess the knockout efficiency of two *Gsn*-targeting sgRNAs in AML12 cells;H-I. Western blot analysis was used to assess the knockdown efficiency of three *Gsn*-targeting siRNAs in AML12 cells;J. Quantitative PCR analyses were used to assess the knocdown efficiency of three *Gsn*-targeting siRNAs in AML12 cells;K-L. Western blot (WB) analysis was performed to evaluate the effect of the PLVX-FLAG-*Gsn* overexpression plasmid；M. Quantitative PCR analysis was performed to evaluate the effect of the PLVX-FLAG-*Gsn* overexpression plasmid.Data were expressed as the means ± standard deviation (SD). Significant differences were determined using one-way ANOVA as appropriate. A 2-tailed p < 0.05 was considered statistically significant. (*p<0.05, **p <0.01, ***p <0.005, ****p <0.001, n.s., no signiﬁcance).

**Figure S6**

**
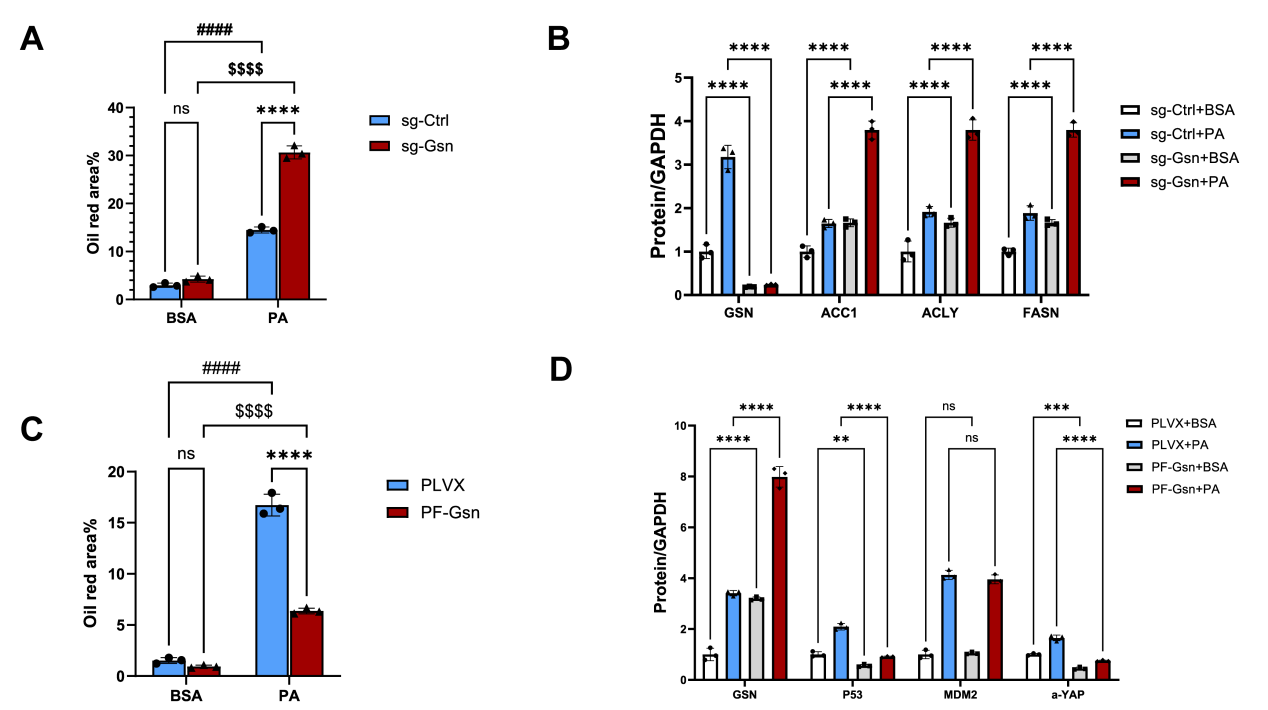
**

**Figure S6. GSN Inhibits Inflammatory Responses and lipid Accumulation in AML12 cells.**A-B. Quantitative analysis of Oil Red O staining results in differently treated AML12 cells in Figure 4E-F.C-D. Quantitative data of Western blot results from Figure 4I-J;Data were expressed as the means ± standard deviation (SD). Significant differences were determined using one-way ANOVA as appropriate. A 2-tailed p < 0.05 was considered statistically significant. (*p<0.05, **p <0.01, ***p <0.005, ****p <0.001, n.s., no signiﬁcance).

**Figure S7**

**
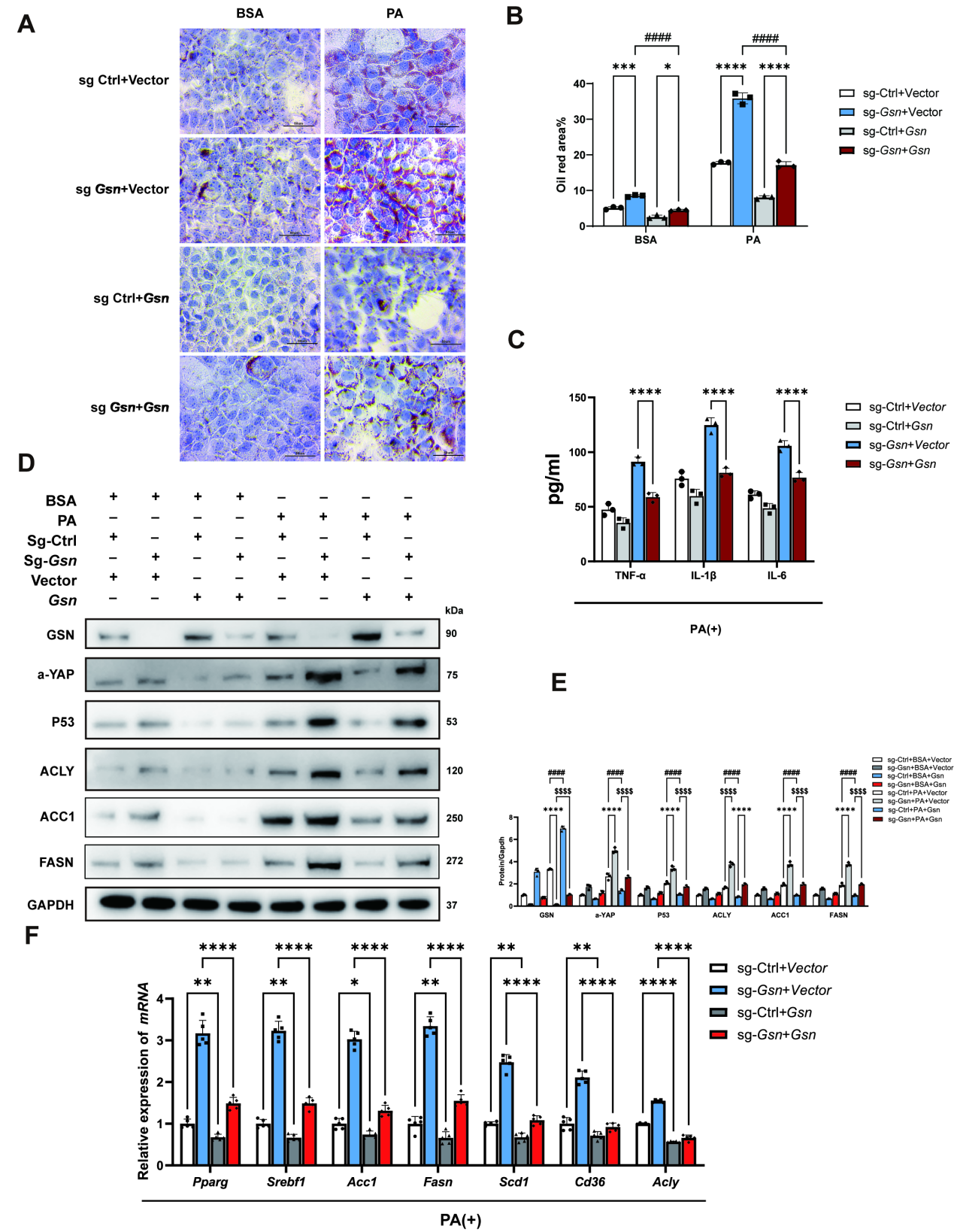
**

**Figure S7. Restoration of GSN expression in sg-*Gsn* AML12 cells alleviated PA-induced lipid accumulation and elevated inflammation levels.**A-B. Representative images of Oil Red O staining in AML12 cells from different treatment groups (sg-Ctrl+Vector, sg-*Gsn*+Vector, sg-Ctrl+*Gsn*, sg-*Gsn*+*Gsn*) after 24 hours of BSA or PA exposure;C. ELISA results of the expression of specified inflammatory Cytokines (TNF-α，IL-1β，IL-6) in the supernatants of AML12 (sg-Ctrl+Vector, sg-*Gsn*+Vector, sg-Ctrl+*Gsn*, sg-*Gsn*+*Gsn*) cells treated with PA;D-E. Western blot results of the protein expression of the specified molecules in AML12 (sg-Ctrl+Vector, sg-*Gsn*+Vector, sg-Ctrl+*Gsn*, sg-*Gsn*+*Gsn*) cells treated with BSA or PA;F. Quantitative PCR results of the mRNA expression of the specified molecules in AML12 (sg-Ctrl+Vector, sg-*Gsn*+Vector, sg-Ctrl+*Gsn*, sg-Gsn+*Gsn*) cells treated with PA;Data were expressed as the means ± standard deviation (SD). Significant differences were determined using one-way ANOVA as appropriate. A 2-tailed p < 0.05 was considered statistically significant. (*p<0.05, **p <0.01, ***p <0.005, ****p <0.001, n.s., no signiﬁcance).

**Figure S8**

**
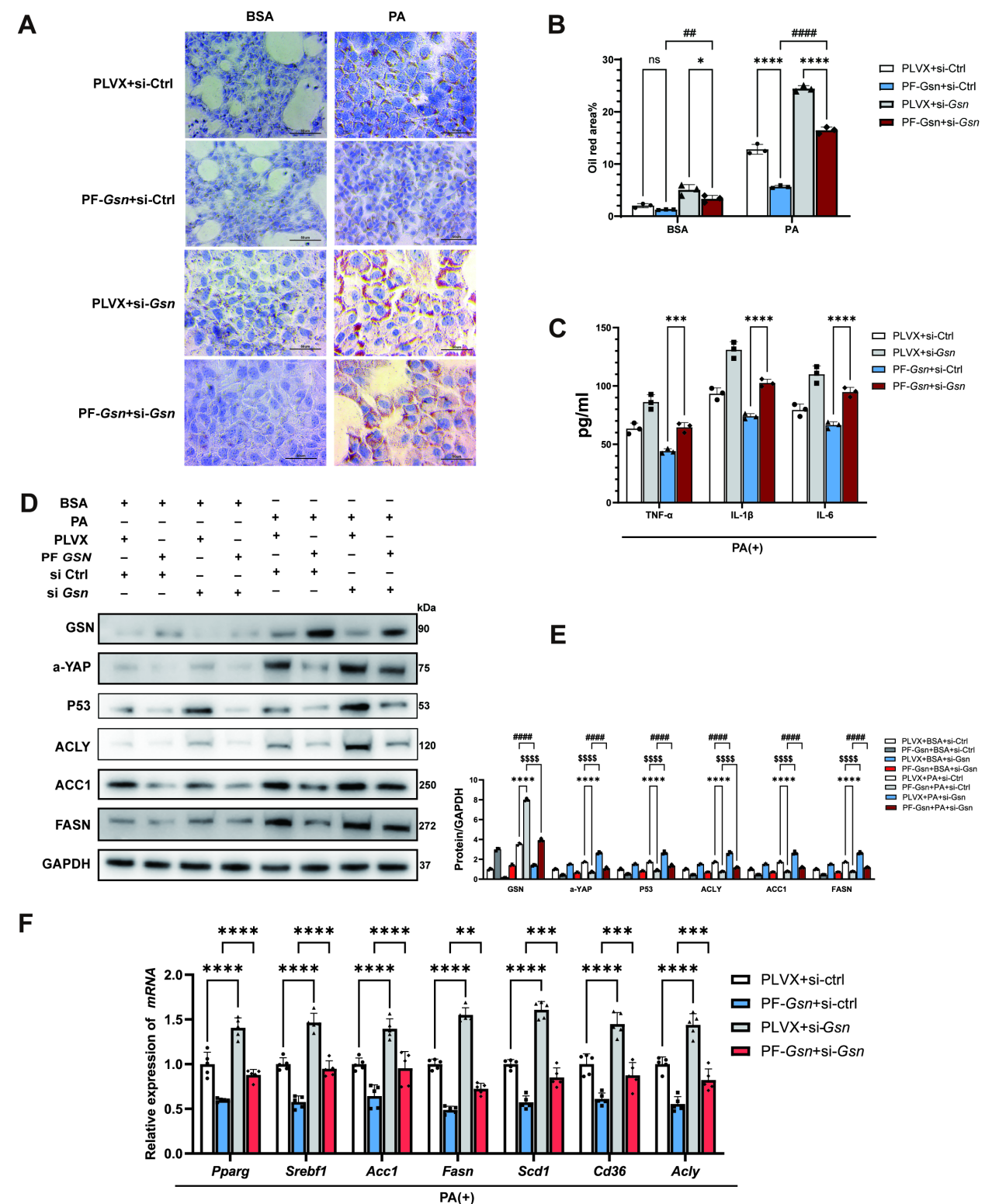
**

**Figure S8. Si-*Gsn* negated the alleviation of PA-induced lipid accumulation and inflammation in AML12 cells by *GSN* overexpression.**A-B. Representative images of Oil Red O staining in AML12 cells from different treatment groups (PLVX+si-Ctrl, PF-*Gsn*+si-Ctrl, PLVX+si-*Gsn*, PF-*Gsn*+si-*Gsn*) after 24 hours of BSA or PA exposure;C. ELISA results of the expression of specified inflammatory Cytokines (TNF-α，IL-1β，IL-6) in the supernatants of AML12 (PLVX+si-Ctrl, PF-*Gsn*+si-Ctrl, PLVX+si-*Gsn*, PF-*Gsn*+si-*Gsn*) cells treated with PA;D-E.Western blot results of the protein expression of the specified molecules in AML12 (PLVX+si-Ctrl, PF-*Gsn*+si-Ctrl, PLVX+si-*Gsn*, PF-Gsn+si-*Gsn*) cells treated with BSA or PA;F. Quantitative PCR results of the mRNA expression of the specified molecules in AML12 (PLVX+si-Ctrl, PF-*Gsn*+si-Ctrl, PLVX+si-*Gsn*, PF-GSN+si-*Gsn*) cells treated with PA;Data were expressed as the means ± standard deviation (SD). Significant differences were determined using one-way ANOVA as appropriate. A 2-tailed p < 0.05 was considered statistically significant. (*p<0.05, **p <0.01, ***p <0.005, ****p <0.001, n.s., no signiﬁcance).

**Figure S9**

**
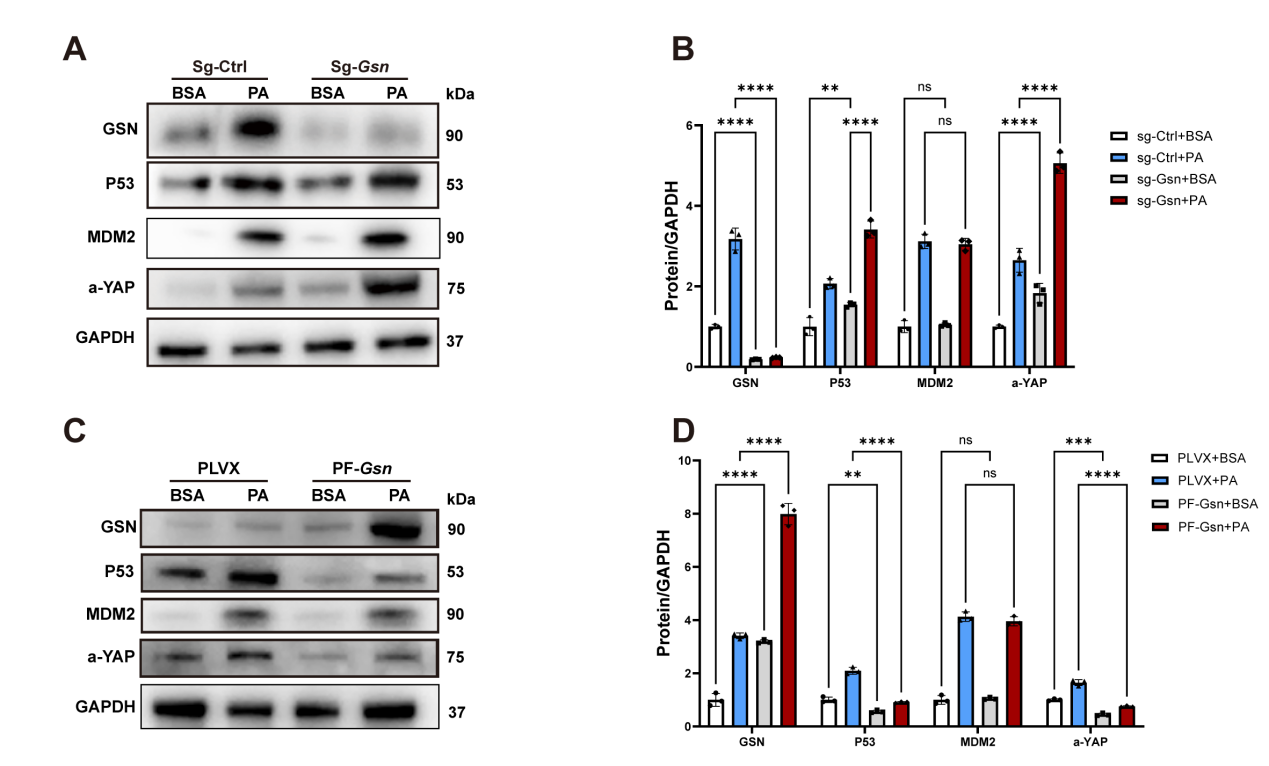
**

**Figure S9. Effects of *Gsn* knockdown and overexpression on key effector molecules.**A-B. The protein expression levels of GSN, a-YAP, P53, MDM2 in AML12 cells treated with PA across different experimental groups (sg-Ctrl, sg-*Gsn*) treated with BSA or PA (n=3/group);C-D. The protein expression levels of GSN, a-YAP, P53, MDM2 in AML12 cells treated with PA across different experimental groups (PLVX, PF-*Gsn*) treated with BSA or PA (n=3/group).Data were expressed as the means ± standard deviation (SD). Significant differences were determined using one-way ANOVA as appropriate. A 2-tailed p < 0.05 was considered statistically significant. (*p<0.05, **p <0.01, ***p <0.005, ****p <0.001, n.s., no signiﬁcance).

**Figure S10**


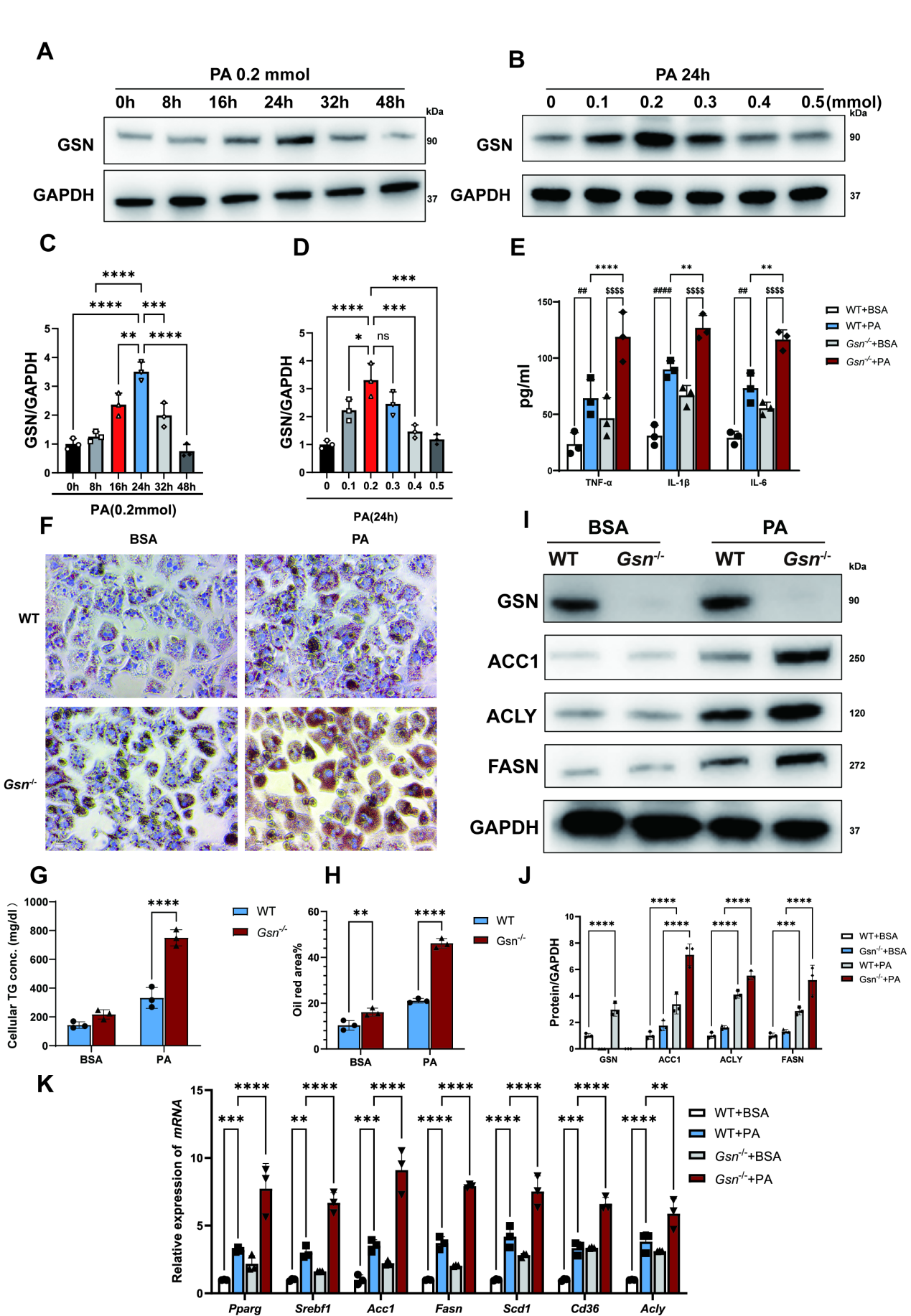


**Figure S10. Effects of PA on primary hepatocytes from WT/*Gsn*^-/-^ Mices** A. Western blot analysis of GSN expression in primary hepatocytes after treatment with PA at varying incubation times (0-48 hours, 0.2mmol PA). GAPDH was used as a loading control;B. Western blot analysis of GSN expression in primary hepatocytes after treatment with PA at varying concentrations (0-0.5 mmol PA, 24h). GAPDH was used as a loading control;

1. Quantification of GSN expression relative to GAPDH in primary hepatocytes treated with PA at varying incubation times (0-48 hours, 0.2mmol PA, n=3/group);D. Quantification of GSN expression relative to GAPDH in primary hepatocytes treated with PA at varying concentrations (0-0.5 mmol PA, 24h, n=3/group);E. ELISA analysis showing the secretion levels of inflammatory cytokines (TNF-α，IL-1β，IL-6) in primary hepatocytes from WT/*Gsn*^-/-^ mices treated with PA or BSA as control (n=3/group);F. Oil red O staining of primary hepatocytes from WT/*Gsn*^-/-^ mices treated with BSA or PA for 24 hours, showing lipid accumulation;G. Quantification of cellular triglyceride (TG) concentrations in primary hepatocytes from WT/*Gsn*^-/-^ mices treated with PA or BSA (n=3/group);H. Quantification of Oil Red O-stained areas in primary hepatocytes from WT/*Gsn*^-/-^ mices treated with PA or BSA (n=3/group);I. Western blot analysis of lipid metabolism markers (ACC1, ACLY, FASN) in primary hepatocytes from WT/*Gsn*^-/-^ mices treated with PA or BSA;J. Relative protein expression of GSN, ACC1, ACLY, FASN in primary hepatocytes from WT/*Gsn*^-/-^ mices treated with PA or BSA (n=3/group);K. Quantification of relative mRNA expression levels of genes involved in lipid metabolism (*Pparg, Srebf1, Acc1, Fasn, Scd1, Cd36, Acly*) in primary hepatocytes from WT/*Gsn*^-/-^ mices treated with PA or BSA (n=3/group);Significant differences were determined using Student’s t test or one-way ANOVA as appropriate. A 2-tailed p < 0.05 was considered statistically significant. (*/#/$p<0.05, **/##/$$p <0.01, ***/###/$$$p <0.005, ****/####/$$$$p <0.001, n.s., no signiﬁcance).

**Figure S11**

**
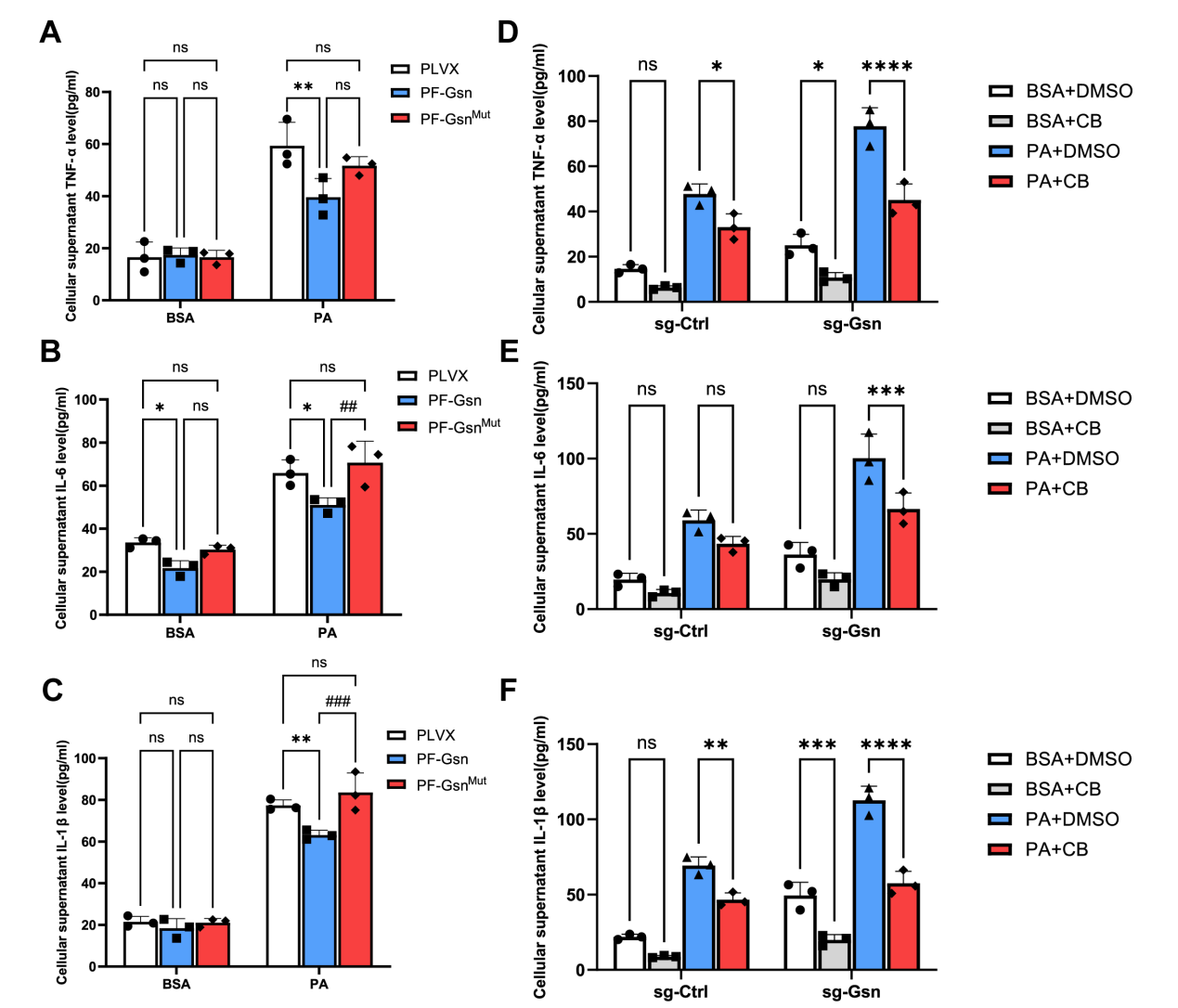
**

**Figure S11. ELISA results in AML12 cells under different conditions.**

A-C. ELISA results of the expression of specified inflammatory Cytokines (TNF-α, IL-1β, IL-6) in AML12 cells (PLVX, PF-*Gsn*, PF-*Gsn*^Mut^) treated with BSA or PA;D-F. ELISA results of the expression of specified inflammatory Cytokines (TNF-α, IL-1β, IL-6) in AML12 cells (sg-Ctrl, sg-*Gsn*) treated under different condition (BSA+DMSO, BSA+CB, PA+DMSO, PA+CB).Data were expressed as the means ± standard deviation (SD). Significant differences were determined using one-way ANOVA as appropriate. A 2-tailed p < 0.05 was considered statistically significant. (*p<0.05, **p <0.01, ***p <0.005, ****p <0.001, n.s., no signiﬁcance).

**Figure S12**

**
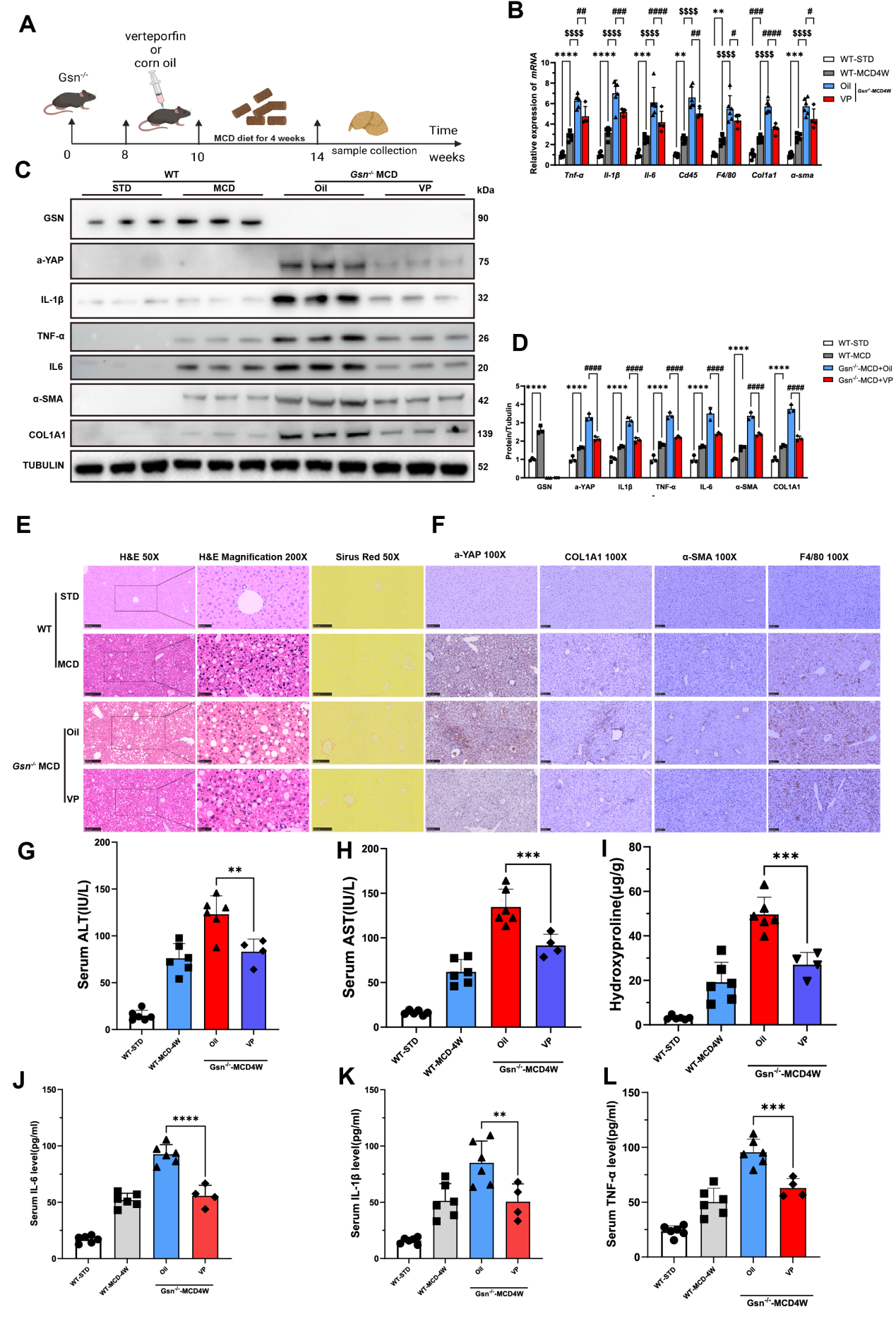
**

**Figure S12**. **In Vivo Verteporfin Injection Alleviates Inflammation and Fibrosis in the Liver Tissues of MASH-Model *Gsn*^-/-^ Mice** A. Experimental Design Workflow Diagram;

B. Quantitative PCR analyses were performed to assess the expression of inflammatory response markers and fibrosis-related markers in liver tissues of WT/*Gsn*^-/-^ mice for each experimental group (n=4-6/group);C-D. Western blot analyses were performed to assess the expression of inflammatory response markers and fibrosis-related markers in liver tissues of WT/*Gsn*^-/-^ mice for each experimental group (n=3/group);E. Representative images from hematoxylin and eosin (H&E) staining (50x, Scale bars :250 µm), H&E Magnification (200x, Scale bars :50 µm), sirius red staining (50x, Scale bars: 250 µm) were obtained for each experimental group (n=4-6/group);F.Representative immunohistochemical (IHC) staining images of a-YAP (100x, Scale bars: 100 µm), α-SMA (100x, Scale bars: 100 µm), COL1A1 (100x, Scale bars: 100 µm), F4/80 (100x, Scale bars: 100 µm) were obtained for each experimental group (n=4-6/group);G-H. Serum levels of ALT and AST in the specified groups of mice (n=4-6/group);I. Hepatic hydroxyproline levels in the liver tissues of the specified groups of mice (n=4-6/group);J-K. ELISA results for inflammatory cytokines (TNF-α, IL-1β, IL-6) in serum of the specified groups of mice (n=4-6/group);Data were expressed as the means ± standard deviation (SD). Significant differences were determined using one-way ANOVA as appropriate. A 2-tailed p < 0.05 was considered statistically significant. (*p<0.05, **p <0.01, ***p <0.005, ****p <0.001, n.s., no signiﬁcance).

**Figure S13**

**
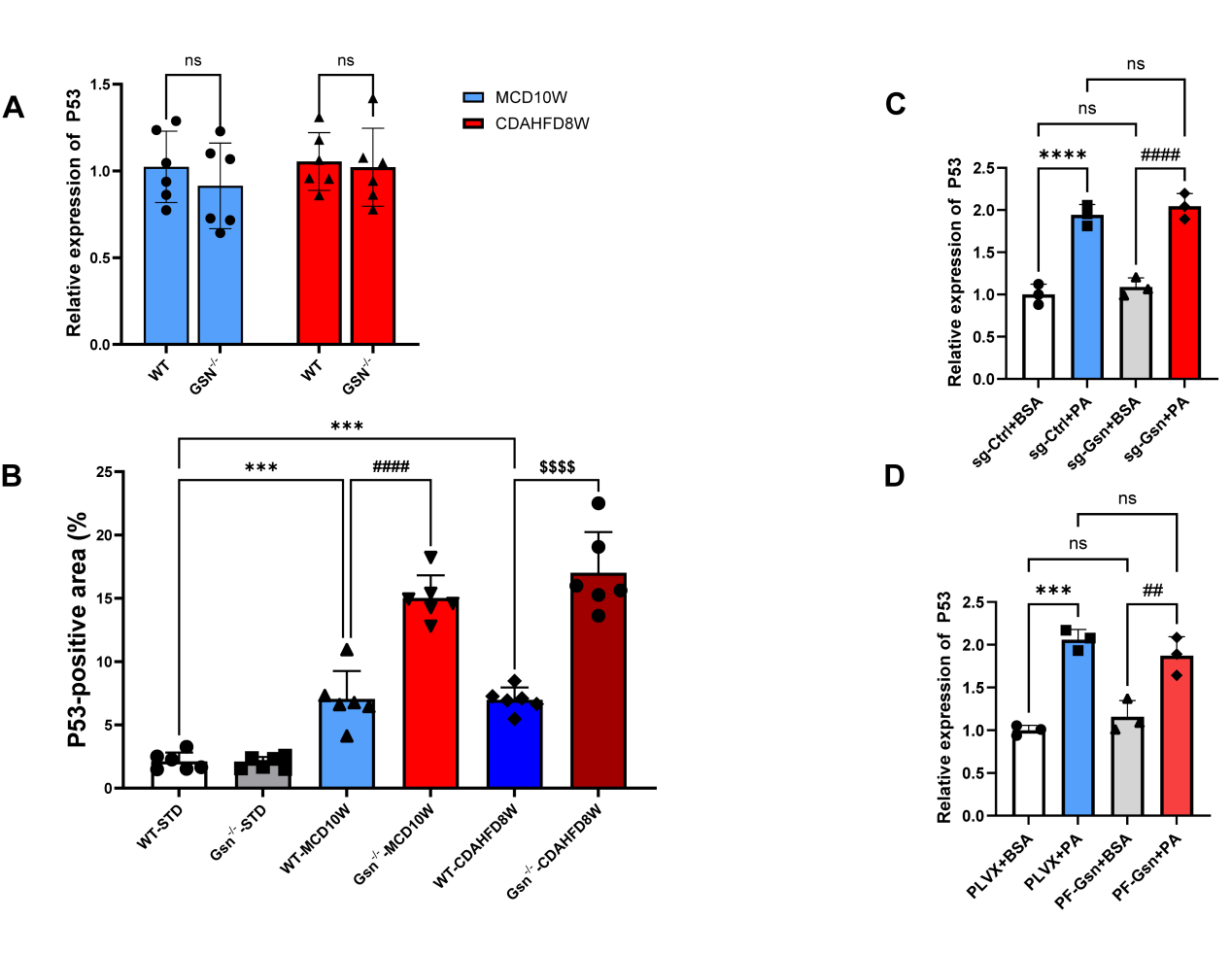
**

**Figure S13. The expression of P53 in liver tissues and AML12 cells under MASH conditions.**A.Q-PCR analysis results of the expression of *P53* in liver tissues of WT/Gsn^-/-^ mice fed with MCD or CDAHFD diets, (n=6/group);B.Quantitative data of immunohistochemistry (IHC) results from Figure 7E;C.Q-PCR analysis results of the expression of *P53* in AML12 cells of different groups (sg-Ctrl+BSA, sg-*Gsn*+BSA, sg-Ctrl+PA, sg-*Gsn*+PA), (n=3/group);D.Q-PCR analysis results of the expression of *P53* in AML12 cells of different groups (PLVX+BSA, PF-*Gsn*+BSA, PLVX+PA, PF-*Gsn*+PA), (n=3/group).Data were expressed as the means ± standard deviation (SD). Significant differences were determined using one-way ANOVA as appropriate. A 2-tailed p < 0.05 was considered statistically significant. (*p<0.05, **p <0.01, ***p <0.005, ****p <0.001, n.s., no signiﬁcance).

**Figure S14**

**
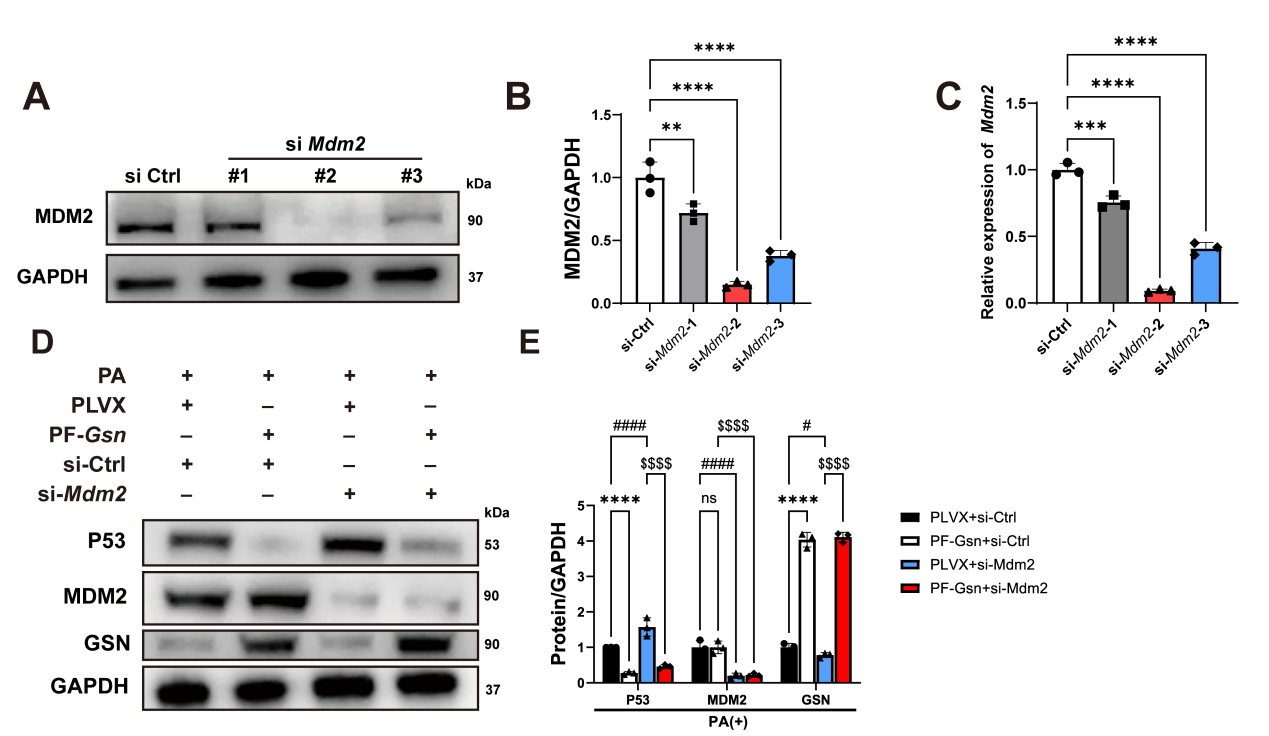
**

**Figure S14. MDM2 is a key molecule mediating GSN's role in promoting P53 ubiquitination.** A-B. Western blot analysis was used to assess the knockdown efficiency of three *Mdm2*-targeting siRNAs in AML12 cells;C. Quantitative PCR analyses were used to assess the knockdown efficiency of three *Mdm2*-targeting siRNAs in AML12 cells;D. The protein expression levels of GSN, P53, MDM2 in AML12 cells treated with PA across different experimental groups (si-Ctrl, si-*Gsn*) (n=3/group);E. Quantitative data of Western blot results from panel D;Data were expressed as the means ± standard deviation (SD). Significant differences were determined using one-way ANOVA as appropriate. A 2-tailed p < 0.05 was considered statistically significant. (*p<0.05, **p <0.01, ***p <0.005, ****p <0.001, n.s., no signiﬁcance).

**Figure S15**

**
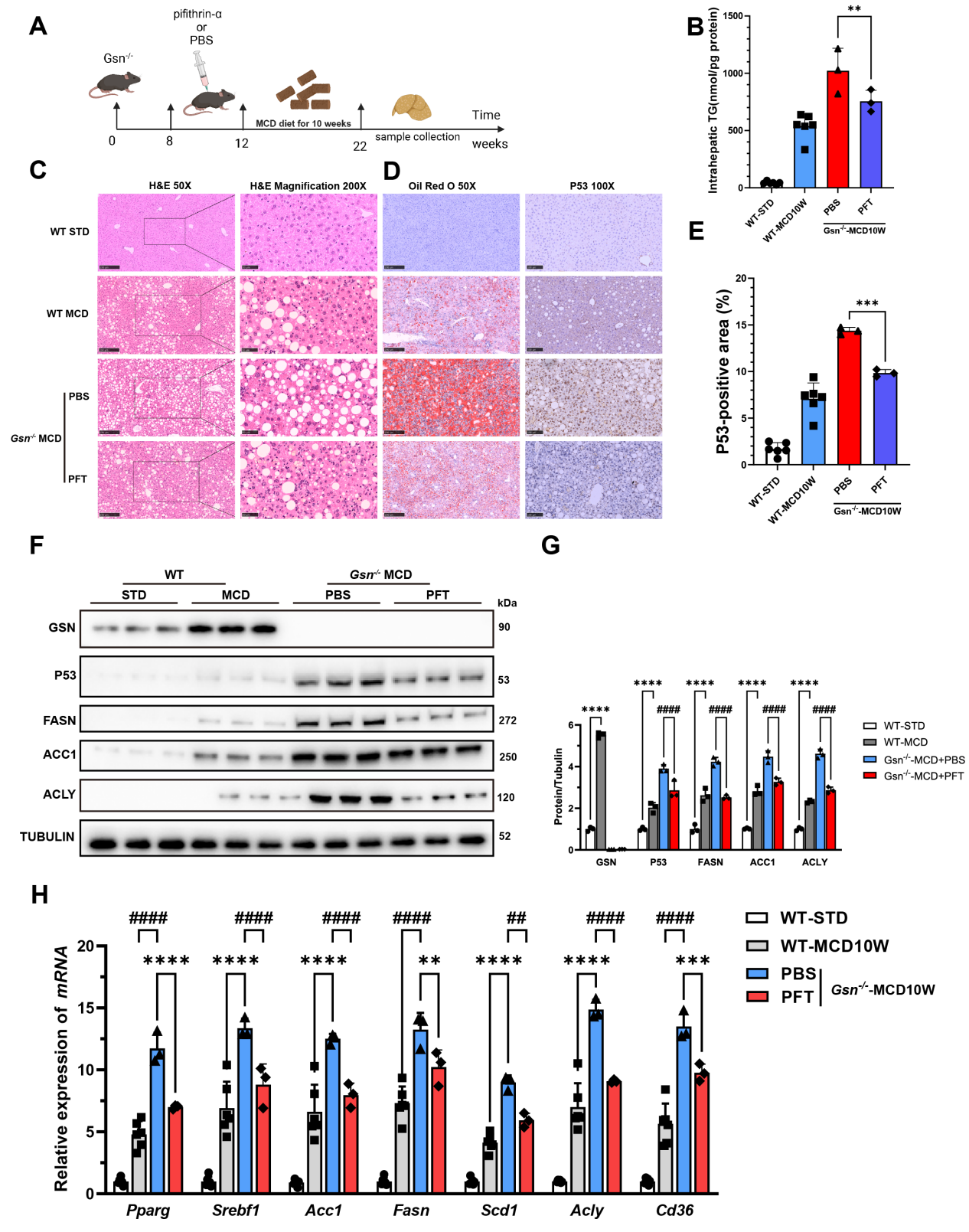
**

**Figure S15. Inhibition of P53 slowed the process of lipid accumulation in vivo.** A.Experimental Design Workflow Diagram;B.Hepatic triglyceride levels in the liver tissues of the specified groups of mice (n=3-6/group);C. Representative images from hematoxylin and eosin (H&E) staining (50x, Scale bars :250 µm), H&E Magnification (200x, Scale bars :50 µm), Oil red O staining (50x, Scale bars: 250 µm) were obtained for each experimental group (n=3-6/group);D.Representative immunohistochemical (IHC) staining images of P53 (100x, Scale bars: 100 µm) were obtained for each experimental group (n=3-6/group);E.Quantitative analysis of P53-positive areas in immunohistochemistry from panel D;F-G. Western blot analyses were performed to assess the expression of lipid metabolism markers in liver tissues of WT/*Gsn*^-/-^ mice for each experimental group (n=3/group);H. Quantitative PCR analyses were performed to assess the expression of lipid metabolism markers in liver tissues of WT/*Gsn*^-/-^ mice for each experimental group (n=3-6/group);Data were expressed as the means ± standard deviation (SD). Significant differences were determined using one-way ANOVA as appropriate. A 2-tailed p < 0.05 was considered statistically significant. (*p<0.05, **p <0.01, ***p <0.005, ****p <0.001, n.s., no signiﬁcance).

**Figure S16**

**
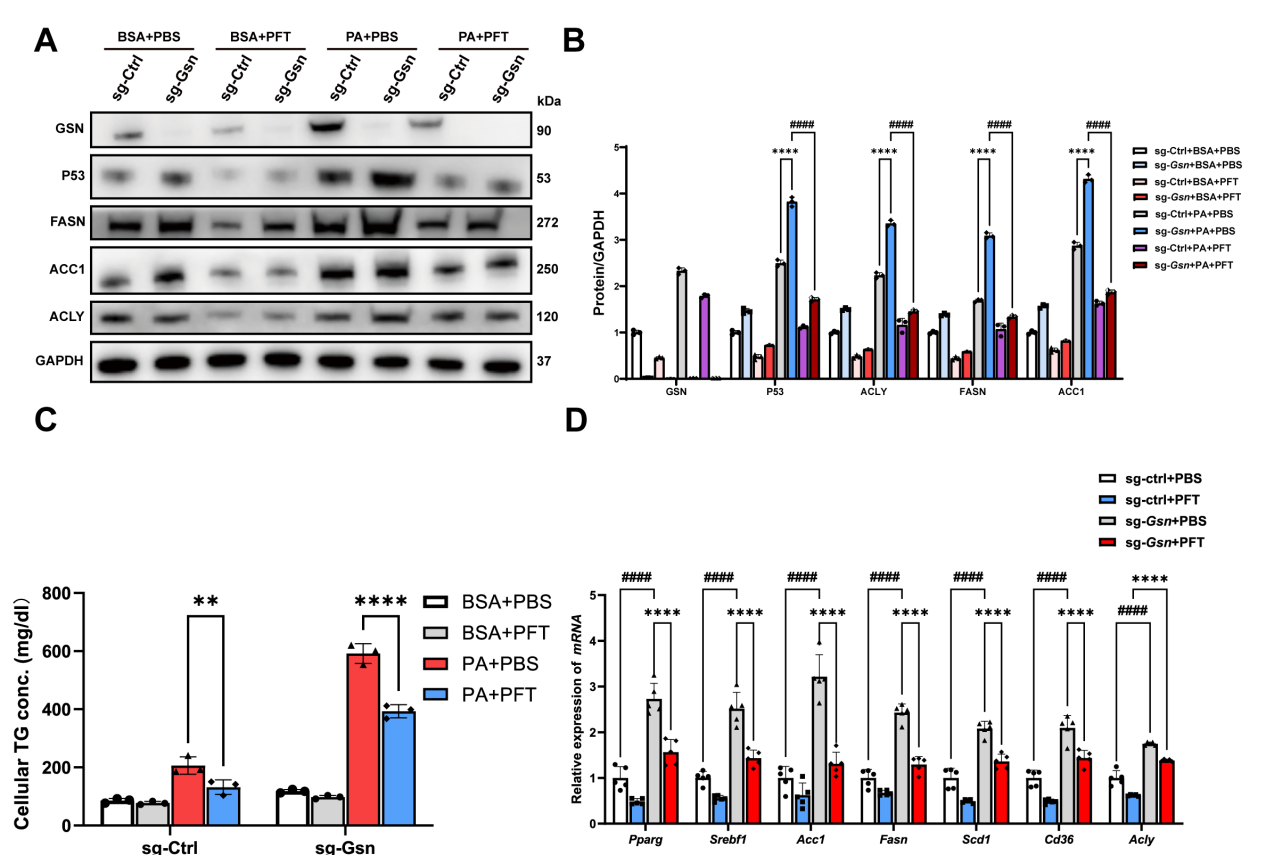
**

**Figure S16. Inhibition of P53 slowed the process of lipid accumulation in vitro**

A. The protein expression levels of GSN, P53, FASN, ACC1 and ACLY in AML12 cells treated with either BSA or PA across different experimental groups (n=3/group);B. Quantitative data of Western blot results from panel A;C. Triacylglycerol levels in AML12 (sg-Ctrl+PBS, sg-*Gsn*+PBS, sg-Ctrl+PFT, sg-*Gsn*+PFT) cells treated with BSA or PA;D. Quantitative PCR results of the mRNA expression of the specified molecules in AML12 (sg-Ctrl+PBS, sg-*Gsn*+PBS, sg-Ctrl+PFT, sg-*Gsn*+PFT) cells treated with BSA or PA.Data were expressed as the means ± standard deviation (SD). Significant differences were determined using one-way ANOVA as appropriate. A 2-tailed p < 0.05 was considered statistically significant. (*p<0.05, **p <0.01, ***p <0.005, ****p <0.001, n.s., no signiﬁcance).

**Supplementary Tables**

**Table S1. PCR sequencing and sequence alignment of *Gsn*^-/-^ mice**：

**WT；**

AAAGGTTCTGGAAGAGAGCATGCAGAGTCACTGGGGACTGAGAGATTTGCCACTGCTATCTGGGAGTGTCTATCCATAG

GGGCAGAGAGTGCCTTTGGAGTCCAGGGCTATCCCTCTATGTTGTCTCCCAAATGGGGGCCCCTGAAAGACAGCCTGCC

TCTTTGTGCCTTAGCTATCTCCCCAAAAGGGAGGGAGCTCTCTGCCTCCTCTGTCATATCACCAACTAGGAGAGCAGAA

AGGAAGACAGCCTGGCCTGGCCAGAAACTCCCAGAAAGGGGTTGATTCTCCTCTAGCACAGGGAGAGCTCGAAGCTGCC

CTCCCTCATGCCTGGGGTCTCTCCTTGCTGAGACCCACACTTCTGCTCAGGTTCTGTCCTGTCTCTCTGCAG**CCCAGCA**

**CTATGGTGGTGGAGCACCCCGAATTCCTGAAGGCAGGGAAGGAGCCTGGCCTGCAGATCTGGCGTGTGGAGAAGTTTG**

**ACCTGGTGCCTGTGCCCCCCAACCTCTATGGAGACTTCTTCACGGGTGATGCCTATGTCATCCTAAAGACTGTGCAGC**

**TGAGGAATGGGAATCTGCAGTATGACCTCCACTATTGGCTGG**GTGAGGGAGTGCTCCTGCTTAGCCCCTCCCCATCCC

CGACCCCCAACTGTATGGATATACCTGAACTGAGTACTCTCTACACCAGGCCTTTGTTAGTGGTTTCCATGACACCGCT

CTAGAGCCAGCCTGTACAGGTTCAGATCCCAGCTCTGCCCTTTGACCTGTGATCTACCCTTCTGCAGCTCACCAGTGGT

TTTTTCTATAGAATGGTCATGTTGACCGCTATGCTTCCTCAAGAGGACAAACAAGTTTTGTAGATGTCATGGAGACCCC

AAATCCCTCCCCCTACCACCACCCCTGTGCTTTCTTGGATCAGC

***Gsn*^-/-^**

AAAGGTTCTGGAAGAGAGCATGCAGAGTCACTGGGGACTGAGAGATTTGCCACTGCTATCTGGGAGTGTCTATCCATAG

GGGCAGAGAGTGCCTTTGGAGTCCAGGGCTATCCCTCTATGTTGTCTCCCAAATGGGGGCCCCTGAAAGACAGCCTGCC

TCTTTGTGCCTTAGCTATCTCCCCAAAAGGGAGGGAGCTCTCTGCCTCCTCTGTCATATCACCAACTAGGAGAGCAGAA

AGGAAGACAGCCTGGCCTGGCCAGAAACTCCCAGAAAGGGGTTGATTCTCCTCTAGCACAGGGAGAGCTCGAAGCTGCCCTCCCTCATGCCTGGGGTCTCTCCTTGCTGAGACCCACACTTCTGCTCAGGTTCTGTCCTGTCTCTCTGCAG**CCCAGCACTATGGTGGTGGAGCACCCCGAATTCCTGAAGGCAGGGAAGGAGCCTGGCCTGCAGATCTGGCGTGTGGAGAAGTTTGACCTGGTGCCTGTGCCCCCCAACCTCTATGGAGACTTCTTTTCACGGGTGATGCCTATGTCATCCTAAAGACTGTGCAGCTGAGGAATGGGAATCTGCAGTATGACCTCCACTATTGGCTGG**GTGAGGGAGTGCTCCTGCTTAGCCCCTCCCCATCCCCGACCCCCAACTGTATGATATACCTGAACTGAGTACTCTCTACACCAGGCCTTTGTTAGTGGTTTCCATGACACCGCTCTAGAGCCAGCCTGTACAGGTTCAGATCCCAGCTCTGCCCTTTGACCTGTGATCTACCCTTCTGCAGCTCACCAGTGGTTTTTTCTATAGAATGGTCATGTTGACCGCTATGCTTCCTCAAGAGGACAAACAAGTTTTGTAGATGTCATGGAGACCCCAAATCCCTCCCCCTACCACCACCCCTGTGCTTTCTTGGATCAGC

**Table S2. Construction strategy for the mutant *Gsn*.**

ATGGCTCCGCACCGCCCCGCGCCCGCGCTGCTTTGCGCGCTGTCCCTGGCGCTGTGCGCGCTGTCGCTGC
CCGTCCGCGCGGCCACTGCGTCGCGGGGGGCGTCCCAGGCGGGGGCGCCCCAGGGGCGGGTGCCCGAGGC
GCGGCCCAACAGCATGGTGGTGGAACACCCCGAGTTCCTCAAGGCAGGGAAGGAGCCTGGCCTGCAGATC
TGGCGTGTGGAGAAGTTCGATCTGGTGCCCGTGCCCACCAACCTTTATGGAGACTTCTTCACGGGCGACG
CCTACGTCATCCTGAAGACAGTGCAGCTGAGGAACGGAAATCTGCAGTATGACCTCCACTACTGGCTGGG
CAATGAGTGCAGCCAGGATGAGAGCGGG**GCGGCCGCC**ATCTTTACCGTGCAGCTGGATGACTACCTGAAC
GGCCGGGCCGTGCAGCACCGTGAGGTCCAGGGCTTCGAGTCGGCCACCTTCCTAGGCTACTTCAAGTCTG
GCCTGAAGTACAAGAAAGGAGGTGTGGCATCAGGATTCAAGCACGTGGTACCCAACGAGGTGGTGGTGCA
GAGACTCTTCCAGGTCAAAGGGCGGCGTGTGGTCCGTGCCACCGAGGTACCTGTGTCCTGGGAGAGCTTC
AACAATGGCGACTGCTTCATCCTGGACCTGGGCAACAACATCCACCAGTGGTGTGGTTCCAACAGCAATC
GGTATGAA**AGACTGAAG**GCCACACAGGTGTCCAAGGGCAT

mutant：

ATGGCTCCGCACCGCCCCGCGCCCGCGCTGCTTTGCGCGCTGTCCCTGGCGCTGTGCGCGCTGTCGCTGC
CCGTCCGCGCGGCCACTGCGTCGCGGGGGGCGTCCCAGGCGGGGGCGCCCCAGGGGCGGGTGCCCGAGGC
GCGGCCCAACAGCATGGTGGTGGAACACCCCGAGTTCCTCAAGGCAGGGAAGGAGCCTGGCCTGCAGATC
TGGCGTGTGGAGAAGTTCGATCTGGTGCCCGTGCCCACCAACCTTTATGGAGACTTCTTCACGGGCGACG
CCTACGTCATCCTGAAGACAGTGCAGCTGAGGAACGGAAATCTGCAGTATGACCTCCACTACTGGCTGGG
CAATGAGTGCAGCCAGGATGAGAGCGGG**GACGACGAC**ATCTTTACCGTGCAGCTGGATGACTACCTGAAC
GGCCGGGCCGTGCAGCACCGTGAGGTCCAGGGCTTCGAGTCGGCCACCTTCCTAGGCTACTTCAAGTCTG
GCCTGAAGTACAAGAAAGGAGGTGTGGCATCAGGATTCAAGCACGTGGTACCCAACGAGGTGGTGGTGCA
GAGACTCTTCCAGGTCAAAGGGCGGCGTGTGGTCCGTGCCACCGAGGTACCTGTGTCCTGGGAGAGCTTC
AACAATGGCGACTGCTTCATCCTGGACCTGGGCAACAACATCCACCAGTGGTGTGGTTCCAACAGCAATC
GGTATGAA**GCGGCCGCC**GCCACACAGGTGTCCAAGGGCAT

**Table S3. Sequences of the primers used for real-time PCR.**

| **Gene** | **Forward (5’ to 3’)** | **Reverse (3’ to 5’)** |
| --- | --- | --- |
| *Fasn*  mouse | CTGCGGAAACTTCAGGAAATG | GGTTCGGAATGCTATCCAGG |
| *Cd36* mouse | GACTGGGACCATTGGTGATGA | AAGGCCATCTCTACCATGCC |
| *Pparg* mouse | ATTCTGGCCCACCAACTTCGG | TGGAAGCCTGATGCTTTATCCCCA |
| *Srebf1* mouse | CACTTCTGGAGACATCGCAAAC | ATGGTAGACAACAGCCGCATC |
| *Fabp1* mouse | TGGTCCGCAATGAGTTCACCCT | CCAGCTTGACGACTGCCTTGACTT |
| *Acc1*  mouse | GGCCAGTGCTATGCTGAGAT | AGGGTCAAGTGCTGCTCCA |
| *Scd1*  mouse | TCTTCCTTATCATTGCCAACACCA | GCGTTGAGCACCAGAGTGTATCG |
| *Il-6*  mouse | TAGTCCTTCCTACCCCAATTTCC | TTGGTCCTTAGCCACTCCTTC |
| *Tnf-α* mouse | CATCTTCTCAAAATTCGAGTGACAA | TGGGAGTAGACAAGGTACAACCC |
| *Il-1β*  mouse | CCGTGGACCTTCCAGGATGA | GGGAACGTCACACACCAGCA |
| *Gsn*  mouse | ATGGCAGGAGACAGTGAAGC | TTTGATGGTGAGGATGCTGA |
| Yap  mouse | TGTCCCAGATGAACGTCACAGC | TGGTGGCTGTTTCACTGGAGCA |
| *P53*  mouse | TCTGACTGTACCACCATCCACCT | CCTTCTGTCTTCCAGTGTGATG |
| Cxcl10 mouse | CACACCCAGGATGCCAAATG | CTGGCCAGTCTGAGCTGTTG |
| *α-Sma*  mouse | CCAGAGCAAACATCCCCAAA | ACGAGTGGTCACCGGAGTAG |
| *Tgfb1*  mouse | CTCCTGGGAGAAGCTGAGAA | TTGAGGTCCACCACATGTTT |
| *Gapdh* mouse | GGTTGTCTCCTGCGACTTCA | TGGTCCAGGGTTTCTTACTCC |
| *Ctgf*  mouse | GACAGCCAGGAGTTCACATC | GAGGGTGCTTGTTGAGATGG |
| *Timp1*  mouse | CAGCTGACCTCAGATGAGG | GAGTGATGTCGCTTGATGGT |
| *Acly*  mouse | TGGAGCCCACAACACCATCAT | AAACCGATCCCCGATGGTGAG |
| *Cpt1a*  mouse | GGATGGCTATGGTCAAGGTC | GGCCTCACAGACTCCAGGTA |
| *Acadl*  mouse | TCATTGCCAAGGCGGTTGAT | GCCATGGACTCAGTCACATAC |
| *Hadh*  mouse | AAAGAGGTTGGCCTCAGCAG | ATCTCCGCAGGGTCATGTTC |
| *Acaa2*  mouse | CCATGGCAATGACTGCAGAG | GTAGCCAGCATCATTAGCAGC |
| *Acox1*  mouse | CCTGATTCAGCAAGGTACGG | TCGCAGACCCTGAAGAAATC |
| *Hsd17b4*  mouse | AGGAGACCTTGTGGACGAGT | GGAGTCGTAGAGCCGTGTAA |
| *Ech1*  mouse | TCATCACTCGATACCAGGAGAC | GGCACAGTACCGGATGTCA |
| *Ppara*  mouse | CAGTGGGGAGAGAGGACAGA | AGTTCGGGAACAAGACGTTG |
| *Atf3*  mouse | CTCTGCCATCGGATGTCCTC | GTTTCGACACTTGGCAGCAG |

**Table S4 The sequence of the overexpression plasmid**

| **Gene** | **Forward (5’ to 3’)** | **Reverse (3’ to 5’)** |
| --- | --- | --- |
| *Myc-Mdm2* | caagctgtgaccggcgcctacgaattcGCCACCATGGTGAGGAGCAG | CTCGGAGATCAGCTTCTGCTCCTCGAGGGGGAAATAAGTTAGCA CAAT |
| *Flag-Gsn* | CAAGCTGTGACCGGCACCTACGAATTCGCCACCATGGTGGGAGAGGCCAG | CTCGGAGATCAGCTTCTGCTCCTCGAGGGGGAAATAAGTTAGCA CAAT |
| *HA-P53* | aagctgtgaccggcgcctacgaattcGCCACCatggaggagccgcag | CCccATCGATggACCGGTcgGGATCCgtctgagtcaggcccttctg |

**Table S5. Primary and secondary antibodies.**

| **Antibody** | **Source** | **Identifier** |
| --- | --- | --- |
| PRIMARY | | |
| anti-GSN, WB, dil:1/1000;IHC,1:200;IF,:1:200 | Proteintech | Cat No : 11644-2-AP; RRID: AB_2295090 |
| anti-GSN, IP, dil:1/200 | Proteintech | Cat No : 11644-2-AP; RRID: AB_2295090 |
| Anti-P53, WB, dil:1/1000,;IHC,1:200 | Proteintech | Cat No : 10442-1-AP;  RRID:AB_2206609 |
| Anti-P53, IP, dil:1/200 | Proteintech | Cat No : 10442-1-AP  RRID:AB_2206609 |
| anti-MDM2, WB, dil:1/1000;IHC,1:200 | Abcam | ab259265  RRID: NA |
| anti-MDM2, IP, dil:1/200 | Abcam | ab259265  RRID: NA |
| anti-α-SMA, WB, dil:1/1000;IHC,1:200 | Abcam | ab7817  RRID: AB_330333 |
| anti-COL1A1, WB, dil:1/1000;IHC,1:200 | Proteintech | Cat No: 67288-1-Ig: AB_2882554 |
| anti-IL-6, WB, dil:1/1000 | Proteintech | Cat No : 21865-1-AP; RRID: AB_11142677 |
| anti-IL-1β, WB, dil:1/1000 | Proteintech | Cat No : 16806-1-AP; RRID: AB_10646432 |
| anti-TNF-α, WB, dil:1/1000 | Proteintech | Cat No : 17590-1-AP; RRID: AB_2271853 |
| anti-active YAP1, WB, dil:1/1000; IHC,1:200; IF, 1:200 | Abcam | ab205270  ; RRID: NA |
| anti-ACC1, WB, dil:1/1000 | Proteintech | Cat No : 21923-1-AP; RRID: AB_11042445 |
| anti-FASN, WB, dil:1/1000 | Proteintech | Cat No : 10624-2-AP; RRID: AB_2100801 |
| anti-ACLY, WB, dil:1/1000 | Proteintech | Cat No : 15421-1-AP; RRID: AB_2223741 |
| anti-HA, WB, dil:1/1000 | Sigma | Cat#H9658; RRID: AB_260092 |
| anti-FLAG, WB, dil:1/1000; IP, 2 μg | Sigma | Cat#F1804; RRID: AB_439712 |
| anti-MYC, WB, dil:1/1000; IP, 2 μg | Proteintech | Cat No : 60003-2-Ig; RRID: AB_2734122 |
| anti-F4/80, IHF, dil:1/500 | Proteintech | Cat#29414-1-AP; RRID: AB_2918300 |
| anti-CD45, IHF, dil:1/500 | Proteintech | Cat No : 20103-1-AP; RRID:AB_2716813 |
| anti-TUBULIN, WB, dil:1/1000 | Proteintech | Cat No : 10094-1-AP; RRID: AB_2210695 |
| anti-GAPDH, WB, dil:1/1000 | Proteintech | Cat#60004-1-Ig; RRID: AB_2107436 |
| anti-normal rabbit IgG, IP, 2 μg | Cell Signaling Technology | Cat#2729; RRID: AB_1031062 |
| anti-mouse IgG1 isotype control, IP, 2 μg | Cell Signaling Technology | Cat#5415; RRID: AB_10829607 |
| anti-ATF3, WB, dil:1/1000 | Abcam | ab254268 |
| SECONDARY | | |
| HRP-conjugated Affinipure Goat-mouse Ig (H + L), WB, dil:1/5000 | Cell Signaling Technology | Cat#SA00001-1; RRID: N/A |
| HRP-conjugated Affinipure Goat-rabbit Ig (H + L), WB, dil:1/5000 | Cell Signaling Technology | Cat#SA00001-2; RRID: N/A |
| Alexa Fluor 594 Affinipure Goat Anti-Rabbit IgG (H + L), IHF, dil:1/2000 | YEASEN Biotech | Cat# 33112ES60;  RRID:N/A |
| Alexa Fluor 488 Affinipure Goat Anti-Rabbit IgG (H + L), IHF, dil:1/2000 | YEASEN Biotech | Cat# 33116ES60;  RRID:AB_2801422 |
|  |  |  |
